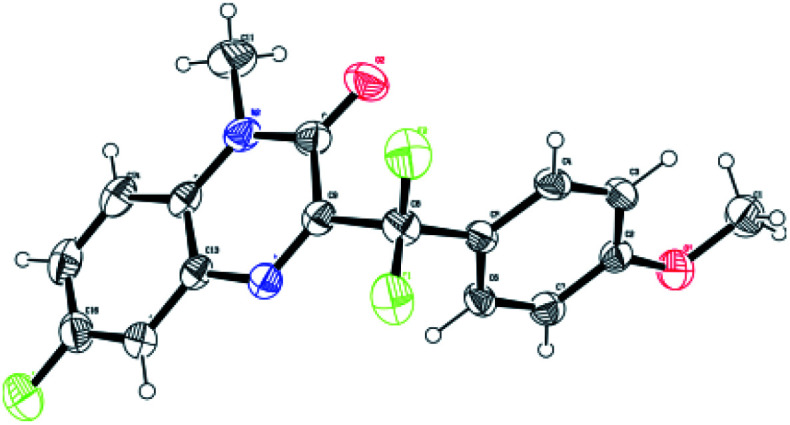# Photoinitiated decarboxylative C3-difluoroarylmethylation of quinoxalin-2(1*H*)-ones with potassium 2,2-difluoro-2-arylacetates in water†

**DOI:** 10.1039/d0ra02059a

**Published:** 2020-03-12

**Authors:** Yanhui Gao, Lulu Zhao, Tianyi Xiang, Pinhua Li, Lei Wang

**Affiliations:** Key Laboratory of Green and Precise Synthetic Chemistry and Applications, Ministry of Education, Department of Chemistry, Huaibei Normal University Huaibei Anhui 235000 P. R. China pphuali@126.com leiwang@chnu.edu.cn; Department of Chemistry, Advanced Research Institute, Taizhou University Taizhou Zhejiang 318000 P. R. China; College of Pharmacy, Shenyang Pharmaceutical University Shenyang 110016 P. R. China

## Abstract

An efficient and green strategy for the preparation of C3-difluoroarylmethylated quinoxalin-2(1*H*)-one *via* a visible-light-induced decarboxylative C3-difluoroarylmethylation of quinoxalin-2(1*H*)-one with potassium 2,2-difluoro-2-arylacetate in water at room temperature was developed. This photoinduced reaction generated the desired products in good yields under simple and mild conditions.

## Abstract 2

An efficient visible-light-induced decarboxylative C3-difluoroarylmethylation of quinoxalin-2(1*H*)-one with potassium 2,2-difluoro-2-arylacetate in water at room temperature was developed.

## Graphical abstract

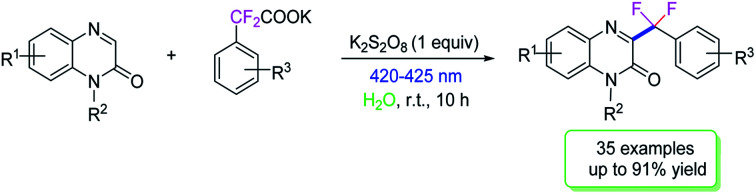

## Introduction

**Scheme 1:**
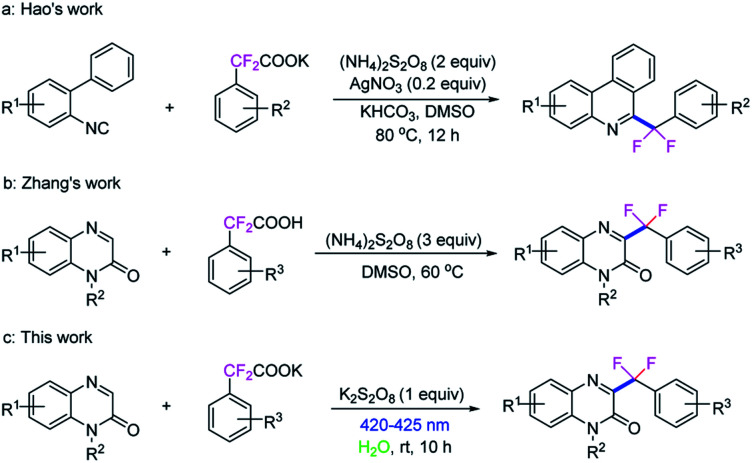
Decarboxylative difluoroarylmethylation reactions.

Organic compounds containing fluorine have been widely found in pharmaceuticals, agrochemicals and materials.^1^ The incorporation of fluorine atoms could remarkably change the physical and biological properties of its parent compounds, such as lipophilicity, stability, and bioavailability.^2^ Among various fluoroalkyl groups, the benzylic difluoromethylene group (ArCF_2_) has attracted much attention in medicinal chemistry, due to the fact that the ArCF_2_ moiety has unique stability, and isosteric properties as an ethereal oxygen atom or a carbonyl group.^3^ So, it is of great value for the construction of fluorinated molecules, especially in the designed structure of drugs. Traditionally, difluoromethylene groups are introduced into the molecular skeleton by a deoxyfluorination of aldehydes or ketones with aminosulfur trifluorides, XeF_2_, or F_2_.^4^ Most recently, transition metals including Cu-, Pd-, and Ni-catalyzed difluoroalkylation reactions have been developed.^5^ As a distinct type of difluorobenzylic compound, difluoroalkylated arenes are present in many bioactive compounds. Therefore, the exploration of practical and broadly applicable methods for the introduction of the ArCF_2_ group into target molecules is in high demand. α,α-Difluoroarylacetic acids and their salts are inexpensive, easy to store and simple to handle fluorine-containing regents, can be readily converted to a variety of useful ArCF_2_-containing compounds. Recently, the decarboxylative coupling of *gem*-difluoroarylacetic acids and their salts have been well established. For example, Wu's group reported a direct decarboxylative alkynylation of α,α-difluoroarylacetic acids under transition metal-free conditions.^6^ Hashmi's group developed a silver-catalyzed decarboxylative alkynylation of α,α-difluoroarylacetic acids with ethynyl-benziodoxolone reagents,^7^ and Hao's group disclosed a silver-catalyzed decarboxylative difluoroarylmethylation of difluoroacetates with isocyanides for constructing 6-*gem*-difluoromethylenated phenanthridines (Scheme 1a).^8^ Very recently, Wan and Hao's group demonstrated a palladium(ii)-catalyzed decarboxylative meta-selective C–H difluoromethylation of arenes from easily accessible difluoroacetic acids, and then a Ag-catalyzed minisci C–H difluoromethylarylation of N-heteroarenes was also developed by the group.^9^ Despite these achievements, it is still desirable to develop practical and mild synthetic methods for the preparation of CF_2_-containing scaffolds.

**Fig. 1:**
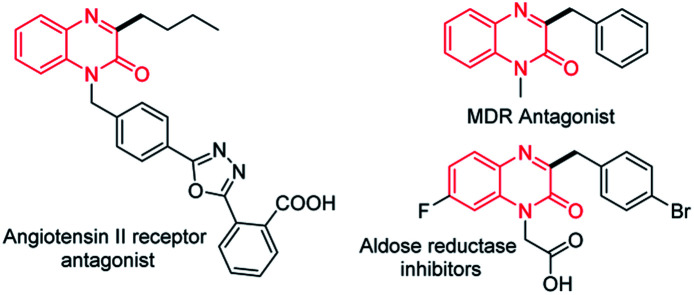
C3 benzyl/alkyl substituted bioactive quinoxalin-2(1*H*)-ones.

Quinoxalin-2(1*H*)-ones, especially the C3-functionalized derivatives are important moieties in pharmaceuticals and materials (Fig. 1).^10^ In the past few years, the C3-functionalizations of quinoxalin-2(1*H*)-ones have become a hot topics,^11^ including C3-arylation,^12^ C3-alkylation,^13^ C3-acylation,^14^ C3-amination,^15^ C3-phosphonation,^16^ C3-alkoxylation,^17^ C3-sulfenylation^18^ and C3-di/trifluoromethylation,^19^ have been extensive investigated. More recently, Zhang *et al.* reported a decarboxylative C3-difluoroarylmethylation of quinoxalin-2(1*H*)-ones with α,α-difluoroarylacetic acids in the presence of (NH_4_)_2_S_2_O_8_ (3.0 equiv.) in DMSO at 60 °C for 18 h (Scheme 1b),^20^ while an excessive dose of oxidant and high temperature was still need in this transformation.

As we all known, visible-light photocatalysis as a powerful tool for organic synthesis^21^ have met to the demands of reaction economy, operational simplicity and environmental friendliness. The organic transformations under visible-light irradiation in the absence of additional photocatalysts have received considerable attention, providing a challenging but meaningful direction for further photochemistry research. Because of our interest in visible-light-induced organic reactions without the photosensitizer,^22^ we here wish to describe a simple and efficient method for the direct C3-difluoroarylmethylation of quinoxalin-2(1*H*)-ones with potassium 2,2-difluoro-2-arylacetates *via* photochemical process without the additional photosensitizer in water under ambient conditions (Scheme 1c).

## Results and discussion

First, *N*-methyl-quinoxalin-2(1*H*)-one (1a) and potassium α,α-difluoro-2-(4-methoxyphenyl)acetate (2a) were used as the model substrates to optimize the reaction conditions, and the results were shown in Table 1. When the model reaction was conducted with 1.0 equivalent of K_2_S_2_O_8_ in DCE at room temperature under the irradiation of blue LED (420–425 nm) for 10 h, only trace amount of the desired product 3a was detected (Table 1, entry 1). To improve yield of the product, a number of solvents were examined. Organic solvents, such as DCE, DMSO, acetone and CH_3_CN show all negative effect to the reaction. To our delight, H_2_O exhibits excellent reactivity, delivering good yield of product 3a in 91% yield. However, co-solvents (DCE/H_2_O and CH_3_CN/H_2_O in 1 : 1 volume ratio) give poor reactivity (Table 1, entries 2–7). The structure of 3a was characterized by ^1^H, ^13^C and ^19^F NMR, and the structure of 3i was further confirmed by X-ray single crystal analysis.^24^ In the absence of visible-light irradiation, no desired product was formed (Table 1, entry 8). A number of oxidants were also tested for the model reaction, and the results indicated that (NH_4_)_2_S_2_O_8_ is another effective oxidant, while no reactivity of BPO, DCP, BI-OH, TBHP and DTBP, and less reactivity of BQ, H_2_O_2_, generating 3a in 54% and 47% yields, respectively (Table 1, entries 9–16). When the model reaction was performed in the presence of oxygen atmosphere without K_2_S_2_O_8_, only 31% yield of 3a was isolated (Table 1, entry 17). It is worth noting that the desired product 3a was also obtained in 90% yield when the reaction was performed under a nitrogen atmosphere, which indicates that oxygen is not required in this transformation (Table 1, entry 18). Subsequently, the wavelength of light source was investigated and blue LED (420–425 nm) was the best choice for the reaction. When the wavelength was less than 420–425 nm or more than 420–425 nm, the results exhibited the less reactivity (Table 1, entries 19–23). Moreover, when sodium α,α-difluoro-2-(4-methoxyphenyl)acetate and α,α-difluorophenylacetic acid were used as substrate, the product 3a was obtained in 89% and 86% yield, respectively (entries 24–25). The loading of oxidant, the ratio of 1a to 2a, as well as the reaction time were optimized, which are also summarized in Table 1 (entries 26–28).

**Table 1:** Optimization of the reaction conditions^a^

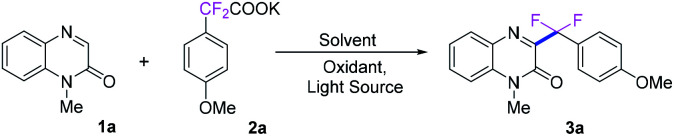				
Entry	Solvent	Oxidant	Light source	Yield^b^ (%)
1	DCE	K_2_S_2_O_8_	420–425 nm	Trace
2	DMSO	K_2_S_2_O_8_	420–425 nm	Trace
3	Acetone	K_2_S_2_O_8_	420–425 nm	NR
4	CH_3_CN	K_2_S_2_O_8_	420–425 nm	NR
5	H_2_O	K_2_S_2_O_8_	420–425 nm	91
6	DCE : H_2_O (1 : 1)	K_2_S_2_O_8_	420–425 nm	42
7	CH_3_CN : H_2_O (1 : 1)	K_2_S_2_O_8_	420–425 nm	<5
8	H_2_O	K_2_S_2_O_8_	In dark	0
9	H_2_O	BPO	420–425 nm	Trace
10	H_2_O	DCP	420–425 nm	Trace
11	H_2_O	BI-OH	420–425 nm	Trace
12	H_2_O	TBHP	420–425 nm	NR
13	H_2_O	DTBP	420–425 nm	NR
14	H_2_O	(NH_4_)_2_S_2_O_8_	420–425 nm	82
15	H_2_O	BQ	420–425 nm	54
16	H_2_O	H_2_O_2_	420–425 nm	47
17	H_2_O	O_2_	420–425 nm	31^c^
18	H_2_O	K_2_S_2_O_8_	420–425 nm	90^d^
19	H_2_O	K_2_S_2_O_8_	380–385 nm	75
20	H_2_O	K_2_S_2_O_8_	390–395 nm	74
21	H_2_O	K_2_S_2_O_8_	410–415 nm	84
22	H_2_O	K_2_S_2_O_8_	450–455 nm	86
23	H_2_O	K_2_S_2_O_8_	Sunlight	65
24	H_2_O	K_2_S_2_O_8_	420–425 nm	89^e^
25	H_2_O	K_2_S_2_O_8_	420–425 nm	86^f^
26	H_2_O	K_2_S_2_O_8_	420–425 nm	64^g^, 90^h^
27	H_2_O	K_2_S_2_O_8_	420–425 nm	61^i^, 91^j^
28	H_2_O	K_2_S_2_O_8_	420–425 nm	75^k^, 89^l^

a Reaction conditions: *N*-methyl-quinoxalin-2(1*H*)-one (1a, 0.10 mmol), potassium 2,2-difluoro-2-(4-methoxyphenyl)acetate (2a, 0.15 mmol), oxidant (1.0 equiv.), solvent (3.0 mL) at room temperature under light irradiation (1.5 W) in air for 10 h.

b Isolated yield. NR = no reaction.

c Oxygen balloon instead of K_2_S_2_O_8_.

d Nitrogen atmosphere.

e Sodium α,α-difluoro-2-(4-methoxyphenyl) acetate was instead of 2a.

f α,α-Difluorophenylacetic acid was instead of 2a.

g K_2_S_2_O_8_ (0.75 equiv.).

h K_2_S_2_O_8_ (1.5 equiv.).

i 2a (0.1 mmol, 1.0 equiv.).

j 2a (0.2 mmol, 2.0 equiv.).

k 8 h.

l 12 h.

**Scheme 2:**
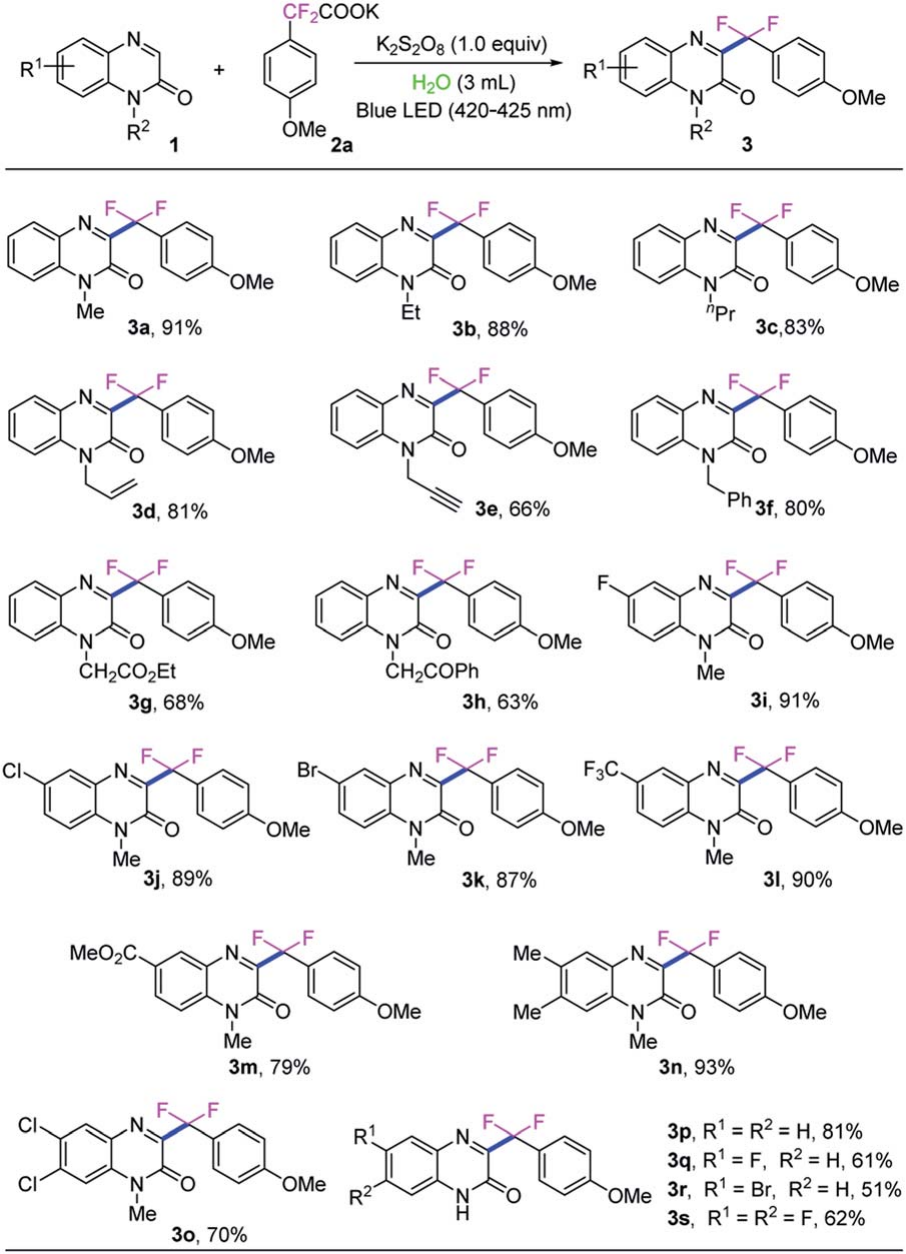
The scope of quinolin-2(1*H*)-ones [reaction conditions: quinoxalin-2(1*H*)-one (1, 0.10 mmol), potassium 2,2-difluoro-2-phenylacetate (2a, 0.15 mmol), K_2_S_2_O_8_ (1.0 equiv.), H_2_O (3.0 mL) at room temperature with blue LED (420–425 nm, 1.5 W) irradiation in air for 10 h; isolated yield of the product].

With the optimized reaction conditions in hand, we next investigated the generality of this direct C3-difluoroarylmethylation reaction. A variety of *N*-substituted quinoxalin-2(1*H*)-ones were subjected to the reaction, and the results are listed in Scheme 2. In general, all the selected quinoxalin-2(1*H*)-one derivatives could reacted with potassium 2,2-difluoro-2-(4-methoxyphenyl)acetate (2a) very smoothly under the standard conditions, indicating a broad tolerance of substituted groups including an electron-donating and an electron-withdrawing group on aromatic rings of quinoxalin-2(1*H*)-ones. Initial studies were focused on various *N*-protected quinoxalin-2(1*H*)-ones and the desired products 3a–3h were obtained in good to excellent yields. Next, a series of quinoxalin-2(1*H*)-ones bearing substituents on the benzene ring were also investigated under the optimal reaction conditions. In general, the C6-position substituted quinoxalin-2(1*H*)-ones bearing an electron-deficient group (F, Cl, Br, CF_3_, CO_2_CH_3_) could generate the desired products 3i–3m in good to excellent yields (79–91%). Moreover, the dimethyl-substituted substrate 1n, was compatible with the reaction as well, and provided the desired product 3n in 93% yield, while the dichloro-substituted substrate 1o furnished the product 3o in a lower yield of 70%. It should be noted that quinoxalin-2(1*H*)-ones without protecting group were also well matched with the transformation, while the expected products 3p–3s were obtained in middle yields because of the poor solubility of the starting materials and products in water and commonly used organic solvents.

**Scheme 3:**
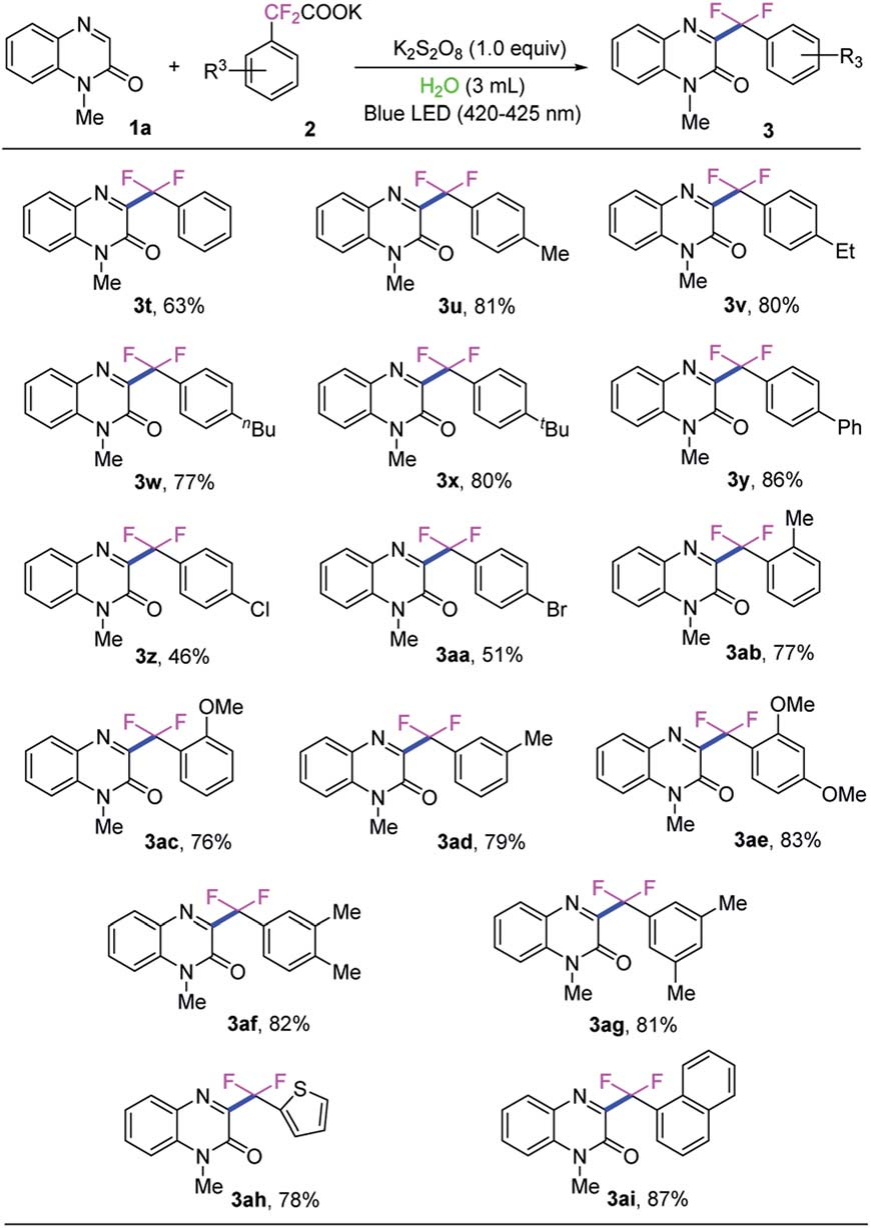
The scope of 2,2-difluoro-2-phenylacetate [reaction conditions: *N*-methyl-quinoxalin-2(1*H*)-one (1a, 0.10 mmol), 2,2-difluoro-2-phenylacetate (2, 0.15 mmol), K_2_S_2_O_8_ (1.0 equiv.), H_2_O (3.0 mL) at room temperature with blue LED (420–425 nm, 1.5 W) irradiation in air for 10 h; isolated yield of the product].

Subsequently, the universality of potassium difluoroarylacetates were further explored, as shown in Scheme 3. Various *para*-substituted potassium α,α-difluoroarylacetates were used as difluoroarylmethylation reagent to react with *N*-methyl-quinoxalin-2(1*H*)-one (1a) to afford the corresponding difluoroarylmethylated quinoxalin-ones 3t–3aa in moderate to high yields. Generally, potassium α,α-difluoroarylacetates with electron-donating groups (3u–3y) gave higher yields than those with electron-withdrawing groups (3z–3aa). Compared with the corresponding 4-substituted potassium α,α-difluoroarylacetates. 2-Methyl, 2-methoxyl and 3-methyl substitutions in the arene ring of potassium α,α-difluoroarylacetates could provide the corresponding products in slightly lower yields. Moreover, the di-substituted substrates were also compatible with the reaction as well, and provided the desired product 3ae–3ag in good yields. To our delight, when the heterocyclic potassium difluoroarylacetate was employed, the transformation could also proceed smoothly and the corresponding product 3ah in 78% yield. In particular, naphthyl-based substrate was also examined and showed good reactivity in the reaction, providing 3ai in 87% yield.

**Scheme 4:**
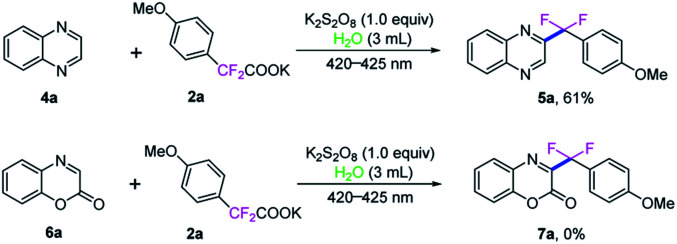
Decarboxylative difluoroarylmethylation of 4a and 6a.

It is important to note that quinoxaline (4a) could also been involved in this direct C3-difluoroarylmethylation reaction, which reacted with potassium 2,2-difluoro-2-(4-methoxyphenyl)acetate (2a) under the standard conditions, providing the corresponding product 5a in 61% yield. However, when 2*H*-benzo[*b*][1,4]oxazin-2-one (6a) was employed in this transformation, no desired product 7a was detected (Scheme 4).

**Scheme 5:**
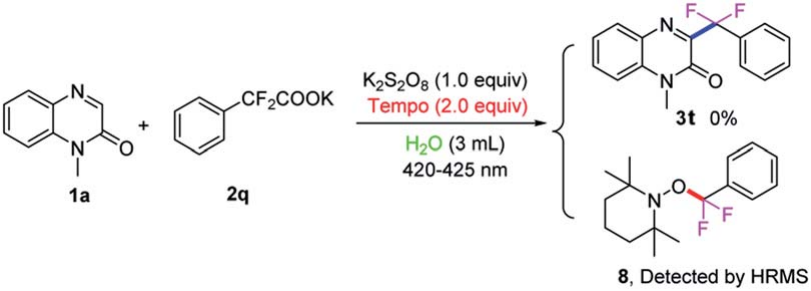
The control experiment.

To further clarify the mechanism of this transformation, the control experiment was conducted, as shown in Scheme 5. When the model reaction was carried out in the presence of radical scavenger 2,2,6,6-tetramethyl-1-piperidinyloxy (TEMPO, 2.5 equiv.) under the standard conditions, no desired product was found, suggesting that a radical process might involve in the reaction. An aryl difluoromethyl radical 
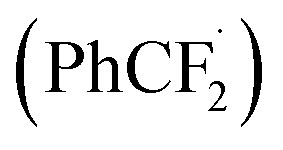
 was trapped with TEMPO under standard reaction conditions to generate the corresponding adduct 8, which was detected by HRMS analysis.

**Scheme 6:**
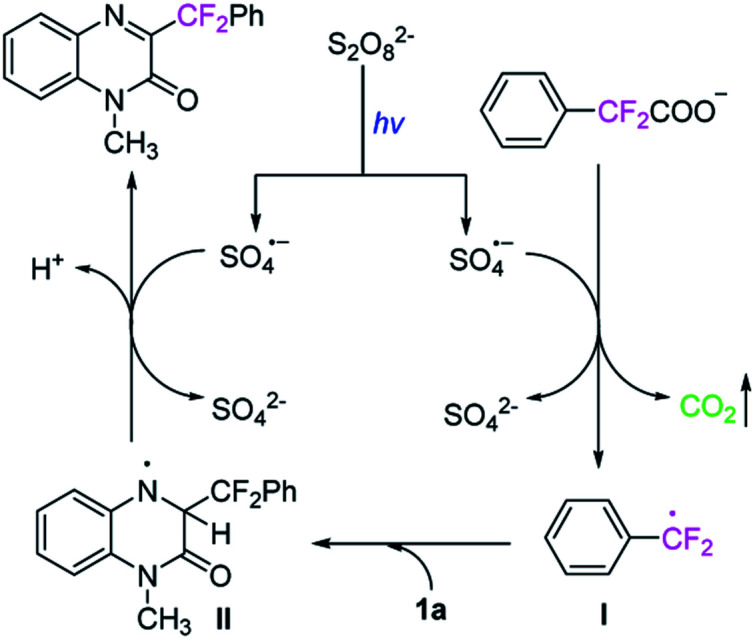
The proposed mechanism.

On the basis of above experimental results and relevant literature,^23^ a plausible mechanism is proposed in Scheme 6. The radical anion SO_4_^−^˙ was firstly generated from K_2_S_2_O_8_ under visible-light irradiation. With the assistance of radical anion SO_4_^−^˙, the potassium 2,2-difluoro-2-phenylacetate 2t undergoes a decarboxylation process to generate a radical intermediate I, releasing carbon dioxide. Then the radical intermediate 
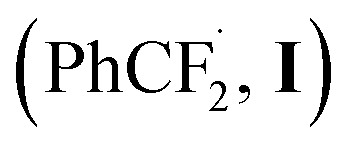
 attacks quinoxalin-2(1*H*)-one 1a at C3-position to generate the radical intermediate II, which is further undergoes single-electron oxidation by loss of H^+^ to afford the product 3t.

## Conclusions

In summary, we have developed an efficient and environment-friendly synthetic protocol for the preparation of C3-difluoroarylmethylated quinoxalin-2(1*H*)-one *via* a visible-light-induced decarboxylative difluoroarylmethylation of quinoxalin-2(1*H*)-one with potassium 2,2-difluoro-2-arylacetate in water under simple and mild conditions. The reaction proceeds smoothly at room temperature afford the corresponding products in moderate to good yields with a broad substituent group tolerance. Further application of this photo-generated difluoroarylmethyl radical to other organic transformations and a detailed mechanistic study are underway in our laboratory.

## Experimental section

### General remarks

The ^1^H NMR, ^13^C NMR and ^19^F NMR spectra were recorded on a 400 MHz or a 600 MHz Bruker FT-NMR spectrometer (400/100/376 MHz or 600/150/564 MHz, respectively). All chemical shifts are given as *δ* value (ppm) with reference to tetramethylsilane (TMS) as an internal standard. The peak patterns are indicated as follows: s, singlet; d, doublet; t, triplet; m, multiplet; q, quartet. The coupling constants, *J*, are reported in hertz (Hz). High resolution mass spectroscopy data of the product were collected on an Agilent Technologies 6540 UHD Accurate-Mass Q-TOF LC/MS (ESI). Melting points (uncorrected) were obtained on WRS-1B digital melting point apparatus. The quinoxalin-2(1*H*)-ones and potassium 2,2-difluoro-2-(4-methoxyphenyl)acetates were prepared according to the reported literature.^8,19*a*^ All the solvents and commercially available reagents were purchased from commercial suppliers. Products were purified by flash chromatography on 200–300 mesh silica gels, SiO_2_.

### Typical procedure for the photoinitiated decarboxylative C3-difluoroarylmethylation

A 5 mL oven-dried reaction vessel equipped with a magnetic stirrer bar was charged with *N*-methyl-quinoxalin-2(1*H*)-one (1a, 0.10 mmol), potassium 2,2-difluoro-2-(4-methoxyphenyl)acetate (2a, 0.15 mmol), K_2_S_2_O_8_ (0.10 mmol) and H_2_O (3.0 mL). The reaction vessel was exposed to a blue LED (420–425 nm, 1.5 W) irradiation at room temperature in air with stirring for 10 h. After completion of the reaction, the mixture was extracted with ethyl acetate and concentrated to yield the crude product, which was further purified by flash chromatography (silica gel, petroleum ether/ethyl acetate = 20 : 1 to 9 : 1) to give the desired product 3a.

### Characterization data for products

#### 3-(Difluoro(4-methoxyphenyl)methyl)-1-methylquinoxalin-2(1*H*)-one (3a)

Yellow solid. Mp 181.4–182.7 °C. ^1^H NMR (400 MHz, CDCl_3_) *δ*: 8.02–8.00 (m, 1H), 7.67–7.65 (m, 2H), 7.63–7.61 (m, 1H), 7.42–7.38 (m, 1H), 7.32–7.30 (m, 1H), 6.92 (d, *J* = 8.8 Hz, 2H), 3.80 (s, 3H), 3.62 (s, 3H); ^13^C NMR (100 MHz, CDCl_3_) *δ*: 160.9, 152.0, 150.7 (t, *J* = 29.2 Hz), 134.1, 132.1, 131.2, 127.4 (t, *J* = 5.8 Hz), 126.9 (t, *J* = 26.6 Hz), 124.0, 117.5 (t, *J* = 245.4 Hz), 113.7, 113.5, 55.2, 28.8; ^19^F NMR (376 MHz, CDCl_3_) *δ*: −98.33. HRMS (ESI) ([M + Na]^+^) calcd for [C_17_H_14_F_2_N_2_NaO_2_]^+^: 339.0916, found: 339.0915.

#### 3-(Difluoro(4-methoxyphenyl)methyl)-1-ethylquinoxalin-2(1*H*)-one (3b)

Yellow solid. Mp 202.1–202.7 °C. ^1^H NMR (400 MHz, CDCl_3_) *δ*: 8.04–8.02 (m, 1H), 7.68–7.61 (m, 3H), 7.41–7.37 (m, 1H), 7.36–7.33 (m, 1H), 6.94–6.92 (m, 2H), 4.26 (t, *J* = 7.2 Hz, 2H), 3.81 (s, 3H), 1.33 (t, *J* = 7.2 Hz, 3H); ^13^C NMR (100 MHz, CDCl_3_) *δ*: 160.9, 151.5, 150.6, 133.2, 132.0, 131.7, 131.6, 127.5 (t, *J* = 5.8 Hz), 127.1, 126.8, 123.8, 117.5 (t, *J* = 245.2 Hz), 113.6, 55.2, 37.3, 12.3; ^19^F NMR (376 MHz, CDCl_3_) *δ*: −98.03. HRMS (ESI) ([M + Na]^+^) calcd for [C_18_H_16_F_2_N_2_NaO_2_]^+^: 353.1072, found: 353.1070.

#### 3-(Difluoro(4-methoxyphenyl)methyl)-1-propylquinoxalin-2(1*H*)-one (3c)

Yellow solid. Mp 170.4–170.6 °C. ^1^H NMR (600 MHz, CDCl_3_) *δ*: 8.02–8.00 (m, 1H), 7.66 (d, *J* = 9.0 Hz, 2H), 7.62–7.60 (m, 1H), 7.38–7.36 (m, 1H), 7.31–7.30 (m, 1H), 6.92–6.91 (m, 2H), 4.14–4.12 (m, 2H), 3.80–3.79 (m, 3H), 1.76–1.70 (m, 2H), 0.99 (t, *J* = 7.2 Hz, 3H); ^13^C NMR (150 MHz, CDCl_3_) *δ*: 160.8, 151.6, 150.5 (t, *J* = 28.2 Hz), 133.3, 131.9, 131.5, 131.4, 127.3 (t, *J* = 5.4 Hz), 127.0 (t, *J* = 26.9 Hz), 123.7, 117.5 (t, *J* = 245.1 Hz), 113.7, 113.5, 55.1, 43.6, 20.4, 11.1; ^19^F NMR (564 MHz, CDCl_3_) *δ*: −97.93. HRMS (ESI) ([M + Na]^+^) calcd for [C_19_H_18_F_2_N_2_NaO_2_]^+^: 367.1229, found: 367.1223.

#### 1-Allyl-3-(difluoro(4-methoxyphenyl)methyl)quinoxalin-2(1*H*)-one (3d)

Yellow solid. Mp 117.7–118.9 °C. ^1^H NMR (400 MHz, CDCl_3_) *δ*: 8.02–8.00 (m, 1H), 7.68–7.65 (m, 2H), 7.61–7.56 (m, 1H), 7.39–7.35 (m, 1H), 7.31–7.29 (m, 1H), 6.91 (d, *J* = 8.8 Hz, 2H), 5.90–5.81 (m, 1H), 5.24–5.22 (m, 1H), 5.16–5.11 (m, 1H), 4.83–4.81 (m, 2H), 3.78 (s, 3H); ^13^C NMR (100 MHz, CDCl_3_) *δ*: 160.8, 151.4, 150.5 (t, *J* = 28.9 Hz), 133.3, 131.9, 131.4, 131.2, 130.1, 127.4 (t, *J* = 5.7 Hz), 126.9 (t, *J* = 27.0 Hz), 123.9, 118.3, 117.5 (t, *J* = 245.5 Hz), 114.2, 113.5, 55.1, 44.2; ^19^F NMR (376 MHz, CDCl_3_) *δ*: −97.82. HRMS (ESI) ([M + Na]^+^) calcd for [C_19_H_16_F_2_N_2_NaO_2_]^+^: 365.1072, found: 365.1077.

#### 3-(Difluoro(4-methoxyphenyl)methyl)-1-(prop-2-yn-1-yl)quinoxalin-2(1*H*)-one (3e)

Yellow solid. Mp 165.5–166.7 °C. ^1^H NMR (600 MHz, CDCl_3_) *δ*: 8.02 (d, *J* = 7.8 Hz, 1H), 7.67–7.65 (m, 3H), 7.49–7.47 (m, 1H), 7.42 (t, *J* = 7.8 Hz, 1H), 6.92–6.91 (m, 2H), 4.97–4.96 (m, 2H), 3.79 (s, 3H); ^13^C NMR (150 MHz, CDCl_3_) *δ*: 160.9, 150.9, 150.5 (t, *J* = 29.3 Hz), 132.6, 132.1, 131.5, 131.3, 127.5 (t, *J* = 5.6 Hz), 126.7 (t, *J* = 26.9 Hz), 124.4, 117.4 (t, *J* = 245.4 Hz), 114.3, 113.6, 76.2, 73.6, 55.2, 31.2; ^19^F NMR (564 MHz, CDCl_3_) *δ*: −97.93. HRMS (ESI) ([M + Na]^+^) calcd for [C_19_H_14_F_2_N_2_NaO_2_]^+^: 363.0916, found: 363.0911.

#### 1-Benzyl-3-(difluoro(4-methoxyphenyl)methyl)quinoxalin-2(1*H*)-one (3f)

Yellow solid. Mp 147.8–150.1 °C. ^1^H NMR (400 MHz, CDCl_3_) *δ*: 8.02–8.00 (m, 1H), 7.70–7.68 (m, 2H), 7.51–7.46 (m, 1H), 7.35–7.31 (m, 1H), 7.28–7.21 (m, 4H), 7.16–7.15 (m, 2H), 6.93 (d, *J* = 8.8 Hz, 2H), 5.40 (s, 2H), 3.79 (s, 3H); ^13^C NMR (100 MHz, CDCl_3_) *δ*: 160.9, 152.1, 150.8 (t, *J* = 29.0 Hz), 134.7, 133.5, 132.0, 131.6, 131.3, 128.9, 127.7, 127.5 (t, *J* = 5.6 Hz), 127.0 (t, *J* = 26.9 Hz), 126.8, 124.0, 117.6 (t, *J* = 245.5 Hz), 114.5, 113.6, 55.2, 45.6; ^19^F NMR (376 MHz, CDCl_3_) *δ*: −97.76. HRMS (ESI) ([M + Na]^+^) calcd for [C_23_H_18_F_2_N_2_NaO_2_]^+^: 415.1229, found: 415.1232.

#### Ethyl 2-(3-(difluoro(4-methoxyphenyl)methyl)-2-oxoquinoxalin-1(2*H*)-yl)acetate (3g)

Yellow solid. Mp 119.1–120.2 °C. ^1^H NMR (400 MHz, CDCl_3_) *δ*: 8.00 (d, *J* = 8.0 Hz, 1H), 7.63 (d, *J* = 8.4 Hz, 2H), 7.57 (t, *J* = 7.6 Hz, 1H), 7.38 (t, *J* = 8.4 Hz, 1H), 7.08 (d, *J* = 8.4 Hz, 1H), 6.90 (d, *J* = 8.4 Hz, 2H), 4.94 (s, 2H), 4.18, (q, *J* = 7.2 Hz, 2H), 3.77 (s, 3H), 1.21 (t, *J* = 7.2 Hz, 3H); ^13^C NMR (100 MHz, CDCl_3_) *δ*: 166.4, 160.8, 151.5, 150.3 (t, *J* = 28.9 Hz), 133.2, 132.1, 131.4, 131.3, 127.3 (t, *J* = 5.6 Hz), 126.7 (t, *J* = 26.4 Hz), 124.2, 117.3 (t, *J* = 245.2 Hz), 113.5, 113.2, 62.0, 55.1, 43.1, 13.8; ^19^F NMR (376 MHz, CDCl_3_) *δ*: −97.76. HRMS (ESI) ([M + Na]^+^) calcd for [C_20_H_18_F_2_N_2_NaO_4_]^+^: 411.1127, found: 411.1125.

#### 1-Benzoyl-3-(difluoro(4-methoxyphenyl)methyl)quinoxalin-2(1*H*)-one (3h)

Yellow solid. Mp 185.6–186.3 °C. ^1^H NMR (400 MHz, CDCl_3_) *δ*: 8.06–8.04 (m, 1H), 8.00–7.98 (m, 2H), 7.66–7.62 (m, 3H), 7.52–7.48 (m, 3H), 7.40–7.36 (m, 1H), 6.97–6.95 (m, 1H), 6.92–6.90 (m, 2H), 5.67 (s, 2H), 3.79 (s, 3H); ^13^C NMR (100 MHz, CDCl_3_) *δ*: 190.5, 160.9, 151.8, 150.4, 134.4, 134.2, 133.7, 132.1, 131.6, 131.5, 129.0, 128.1, 127.4 (t, *J* = 5.6 Hz), 126.9, 124.2, 117.4 (t, *J* = 245.3 Hz), 113.7, 113.6, 55.2, 48.2; ^19^F NMR (376 MHz, CDCl_3_) *δ*: −97.83. HRMS (ESI) ([M + Na]^+^) calcd for [C_24_H_18_F_2_N_2_NaO_3_]^+^: 443.1178, found: 443.1174.

#### 3-(Difluoro(4-methoxyphenyl)methyl)-6-fluoro-1-methylquinoxalin-2(1*H*)-one (3i)

Yellow solid. Mp 191.1–192.4 °C. ^1^H NMR (400 MHz, CDCl_3_) *δ*: 7.73–7.70 (m, 1H), 7.65 (d, *J* = 8.8 Hz, 2H), 7.42–7.37 (m, 1H), 7.31–7.26 (m, 1H), 6.92 (m, 2H), 3.80 (s, 3H), 3.63 (s, 3H); ^13^C NMR (100 MHz, CDCl_3_) *δ*: 161.0, 159.9, 157.4, 152.0 (t, *J* = 29.2 Hz), 151.6, 131.9 (d, *J* = 13.2 Hz), 127.5 (t, *J* = 5.6 Hz), 126.6 (t, *J* = 26.9 Hz), 120.0 (d, *J* = 24.0 Hz), 117.4 (t, *J* = 245.8 Hz), 116.5 (d, *J* = 22.5 Hz), 114.9 (d, *J* = 8.6 Hz), 113.6, 55.2, 29.1; ^19^F NMR (376 MHz, CDCl_3_) *δ*: −98.33, −117.88. HRMS (ESI) ([M + Na]^+^) calcd for [C_17_H_13_F_3_N_2_NaO_2_]^+^: 357.0821, found: 357.0822.

#### 6-Chloro-3-(difluoro(4-methoxyphenyl)methyl)-1-methylquinoxalin-2(1*H*)-one (3j)

Yellow solid. Mp 254.3–255.6 °C. ^1^H NMR (600 MHz, CDCl_3_) *δ*: 8.02 (d, *J* = 2.4 Hz, 1H), 7.64 (d, *J* = 9.0 Hz, 2H), 7.59–7.57 (m, 1H), 7.26–7.25 (m, 1H), 6.93–6.92 (m, 2H), 3.81 (s, 3H), 3.62 (s, 3H); ^13^C NMR (150 MHz, CDCl_3_) *δ*: 161.0, 152.0 (t, *J* = 29.3 Hz), 151.6, 132.9, 132.1, 131.9, 130.5, 129.4, 127.5 (t, *J* = 5.4 Hz), 126.6 (t, *J* = 26.7 Hz), 117.4 (t, *J* = 245.9 Hz), 114.9, 113.6, 55.3, 29.1; ^19^F NMR (564 MHz, CDCl_3_) *δ*: −98.71. HRMS (ESI) ([M + Na]^+^) calcd for [C_17_H_13_ClF_2_N_2_NaO_2_]^+^: 373.0526, found: 373.0528.

#### 6-Bromo-3-(difluoro(4-methoxyphenyl)methyl)-1-methylquinoxalin-2(1*H*)-one (3k)

Yellow solid. Mp 204.7–205.4 °C. ^1^H NMR (400 MHz, CDCl_3_) *δ*: 8.15–8.14 (m, 1H), 7.71–7.68 (m, 1H), 7.63 (d, *J* = 8.8 Hz, 2H), 7.20–7.18 (m, 1H), 6.91 (d, *J* = 8.8 Hz, 2H), 3.80 (s, 3H), 3.60 (s, 3H); ^13^C NMR (100 MHz, CDCl_3_) *δ*: 161.0, 151.8 (t, *J* = 39.3 Hz), 151.6, 134.7, 133.5, 133.3, 132.0, 127.5 (t, *J* = 5.6 Hz), 126.5 (t, *J* = 26.9 Hz), 117.3 (t, *J* = 245.8 Hz), 115.2, 113.6, 55.2, 29.0; ^19^F NMR (376 MHz, CDCl_3_) *δ*: −98.62. HRMS (ESI) ([M + Na]^+^) calcd for [C_17_H_13_BrF_2_N_2_NaO_2_]^+^: 417.0021, found: 417.0020.

#### 3-(Difluoro(4-methoxyphenyl)methyl)-1-methyl-6-(trifluoromethyl)quinoxalin-2(1*H*)-one (3l)

Yellow solid. Mp 164.8–167.2 °C. ^1^H NMR (600 MHz, CDCl_3_) *δ*: 8.302–8.300 (m, 1H), 7.85–7.83 (m, 1H), 7.64 (d, *J* = 8.4 Hz, 2H), 7.43–7.42 (m, 1H), 6.92–6.91 (m, 2H), 3.80 (s, 3H), 3.65 (s, 3H); ^13^C NMR (150 MHz, CDCl_3_) *δ*: 161.1, 152.3 (t, *J* = 29.6 Hz), 151.7, 136.4, 130.6, 128.7 (q, *J* = 3.3 Hz), 127.5 (t, *J* = 5.4 Hz), 126.4 (t, *J* = 33.6 Hz), 126.2 (q, *J* = 26.7 Hz), 124.3, 122.5, 117.3 (t, *J* = 246.0 Hz), 114.5, 113.6, 55.2, 29.1; ^19^F NMR (564 MHz, CDCl_3_) *δ*: −62.13, −98.78. HRMS (ESI) ([M + Na]^+^) calcd for [C_18_H_13_F_5_N_2_NaO_2_]^+^: 407.0789, found: 407.0793.

#### Methyl 3-(difluoro(4-methoxyphenyl)methyl)-1-methyl-2-oxo-1,2-dihydroquinoxaline-6-carboxylate (3m)

Yellow solid. Mp 178.7–180.4 °C. ^1^H NMR (600 MHz, CDCl_3_) *δ*: 8.67–8.66 (m, 1H), 8.26–8.24 (m, 1H), 7.65–7.64 (m, 2H), 7.36–7.35 (m, 1H), 6.92–6.91 (m, 2H), 3.96 (s, 3H), 3.80 (s, 3H), 3.65 (s, 3H); ^13^C NMR (150 MHz, CDCl_3_) *δ*: 165.5, 161.0, 151.5 (t, *J* = 29.3 Hz), 137.3, 132.9, 132.5, 130.6, 127.5 (t, *J* = 5.4 Hz), 126.5 (t, *J* = 26.7 Hz), 125.9, 117.3 (t, *J* = 245.9 Hz), 113.8, 113.6, 55.2, 52.4, 29.1; ^19^F NMR (564 MHz, CDCl_3_) *δ*: −98.63. HRMS (ESI) ([M + H]^+^) calcd for [C_19_H_17_F_2_N_2_O_4_]^+^: 375.1151, found: 375.1152.

#### 3-(Difluoro(4-methoxyphenyl)methyl)-1,6,7-trimethylquinoxalin-2(1*H*)-one (3n)

Yellow solid. Mp 164.8–167.2 °C. ^1^H NMR (400 MHz, CDCl_3_) *δ*: 7.77 (s, 1H), 7.65 (d, *J* = 8.8 Hz, 2H), 7.08 (s, 1H), 6.92 (d, *J* = 8.4 Hz, 2H), 3.81–3.80 (m, 3H), 3.61–3.60 (m, 3H), 2.43 (s, 3H), 2.37 (s, 3H); ^13^C NMR (100 MHz, CDCl_3_) *δ*: 160.8, 152.2, 149.3, 148.9, 142.4, 133.1, 132.3, 131.2, 129.8, 127.4 (t, *J* = 5.7 Hz), 127.0, 117.6 (t, *J* = 244.8 Hz), 114.2, 113.5, 55.2, 28.8, 20.7, 19.1; ^19^F NMR (376 MHz, CDCl_3_) *δ*: −102.79. HRMS (ESI) ([M + Na]^+^) calcd for [C_19_H_18_F_2_N_2_NaO_2_]^+^: 367.1229, found: 367.1224.

#### 6,7-Dichloro-3-(difluoro(4-methoxyphenyl)methyl)-1-methylquinoxalin-2(1*H*)-one (3o)

Yellow solid. Mp 191.6–192.7 °C. ^1^H NMR (400 MHz, CDCl_3_) *δ*: 8.07 (s, 1H), 7.61 (d, *J* = 8.4 Hz, 2H), 7.40 (s, 1H), 6.91 (d, *J* = 8.4 Hz, 2H), 3.80 (s, 3H), 3.57 (s, 3H); ^13^C NMR (100 MHz, CDCl_3_) *δ*: 161.0, 151.9 (t, *J* = 29.4 Hz), 151.3, 136.3, 133.4, 131.9, 130.3, 127.9, 127.5 (t, *J* = 5.7 Hz), 126.3 (t, *J* = 26.7 Hz), 117.2 (t, *J* = 246.2 Hz), 115.2, 113.6, 55.2, 29.1; ^19^F NMR (376 MHz, CDCl_3_) *δ*: −98.07. HRMS (ESI) ([M + Na]^+^) calcd for [C_17_H_12_Cl_2_F_2_N_2_NaO_2_]^+^: 407.0136, found: 407.0137.

#### 3-(Difluoro(4-methoxyphenyl)methyl)quinoxalin-2(1*H*)-one (3p)

Yellow solid. Mp 174.3–175.6 °C. ^1^H NMR (400 MHz, *d*_6_-DMSO) *δ*: 12.72 (s, 1H), 7.90 (d, *J* = 8.0 Hz, 1H), 7.65–7.61 (m, 1H), 7.55–7.53 (m, 2H), 7.38–7.34 (m, 2H), 7.01 (d, *J* = 8.8 Hz, 2H), 3.78 (s, 3H); ^13^C NMR (100 MHz, *d*_6_-DMSO) *δ*: 160.6, 151.9, 133.1, 132.1, 130.3, 129.5, 127.2 (t, *J* = 5.2 Hz), 126.6, 123.7, 117.7 (t, *J* = 243.5 Hz), 113.7, 55.2; ^19^F NMR (376 MHz, *d*_6_-DMSO) *δ*: −95.09. HRMS (ESI) ([M + H]^+^) calcd for [C_16_H_13_F_2_N_2_O_2_]^+^: 303.0940, found: 303.0945.

#### 3-(Difluoro(4-methoxyphenyl)methyl)-6-fluoroquinoxalin-2(1*H*)-one (3q)

Yellow solid. Mp 190.1–191.8 °C. ^1^H NMR (600 MHz, *d*_6_-DMSO) *δ*: 12.81 (s, 1H), 7.81–7.79 (m, 1H), 7.60–7.57 (m, 1H), 7.54 (d, *J* = 9.0 Hz, 2H), 7.39–7.37 (m, 1H), 7.02 (d, *J* = 9.0 Hz, 2H), 3.79 (s, 3H); ^13^C NMR (150 MHz, *d*_6_-DMSO) *δ*: 161.2, 159.2, 157.6, 152.7 (t, *J* = 28.5 Hz), 152.1, 131.0 (d, *J* = 12.0 Hz), 130.6, 127.7 (t, *J* = 4.5 Hz), 126.9 (t, *J* = 27.0 Hz), 120.9 (d, *J* = 25.5 Hz), 118.1 (t, *J* = 243.0 Hz), 117.5 (d, *J* = 9.0 Hz), 115.0 (d, *J* = 22.5 Hz), 114.3, 55.8; ^19^F NMR (564 MHz, *d*_6_-DMSO) *δ*: −95.19, −118.50. HRMS (ESI) ([M + H]^+^) calcd for [C_16_H_11_F_3_N_2_O_2_]^+^: 320.0733, found: 320.0776.

#### 6-Bromo-3-(difluoro(4-methoxyphenyl)methyl)quinoxalin-2(1*H*)-one (3r)

Yellow solid. Mp 200.5–201.9 °C. ^1^H NMR (600 MHz, *d*_6_-DMSO) *δ*: 12.86 (s, 1H), 8.131–8.129 (m, 1H), 7.81–7.94 (m, 1H), 7.54 (d, *J* = 9.0 Hz, 2H), 7.30–7.29 (m, 1H), 7.02 (d, *J* = 8.4 Hz, 2H), 3.79 (s, 3H); ^13^C NMR (150 MHz, *d*_6_-DMSO) *δ*: 161.2, 162.6 (t, *J* = 28.5 Hz), 162.2, 135.2, 133.0, 131.9, 131.7, 127.7 (t, *J* = 4.5 Hz), 126.8 (t, *J* = 27.0 Hz), 118.03 (t, *J* = 243.0 Hz), 118.0, 115.5, 114.3, 55.8; ^19^F NMR (564 MHz, *d*_6_-DMSO) *δ*: −95.23. HRMS (ESI) ([M + H]^+^) calcd for [C_16_H_11_BrF_2_N_2_O_2_]^+^: 379.9972, found: 379.9971.

#### 3-(Difluoro(4-methoxyphenyl)methyl)-6,7-difluoroquinoxalin-2(1*H*)-one (3s)

Yellow solid. Mp 187.3–187.9 °C. ^1^H NMR (600 MHz, *d*_6_-DMSO) *δ*: 12.87 (s, 1H), 8.12–8.10 (m, 1H), 7.53 (d, *J* = 8.4 Hz, 2H), 7.29–7.26 (m, 1H), 7.02 (d, *J* = 9.0 Hz, 2H), 3.79 (s, 3H); ^13^C NMR (150 MHz, *d*_6_-DMSO) *δ*: 161.2, 152.1, 151.9 (t, *J* = 25.5 Hz), 147.3 (d, *J* = 13.5 Hz), 145.7 (d, *J* = 13.5 Hz), 131.5 (d, *J* = 10.5 Hz), 127.7 (t, *J* = 4.5 Hz), 127.2 (d, *J* = 9.0 Hz), 126.8 (t, *J* = 31.5 Hz), 118.0 (t, *J* = 243.0 Hz), 117.8 (d, *J* = 18.0 Hz), 114.3, 103.8 (d, *J* = 21.0 Hz), 55.8; ^19^F NMR (564 MHz, *d*_6_-DMSO) *δ*: −95.19, −129.94 (d, *J* = 23.0 Hz); −143.37 (d, *J* = 23.0 Hz). HRMS (ESI) ([M + H]^+^) calcd for [C_16_H_10_F_4_N_2_O_2_]^+^: 338.0678, found: 338.0674.

#### 3-(Difluoro(phenyl)methyl)-1-methylquinoxalin-2(1*H*)-one (3t)

Yellow solid. Mp 161.1–161.9 °C. ^1^H NMR (600 MHz, CDCl_3_) *δ*: 8.05–8.03 (m, 1H), 7.75–7.73 (m, 2H), 7.67–7.64 (m, 1H), 7.43–7.42 (m, 3H), 7.41–7.40 (m, 1H), 7.33 (d, *J* = 8.4 Hz, 2H), 3.64 (s, 3H); ^13^C NMR (150 MHz, CDCl_3_) *δ*: 152.1, 150.6, 134.9 (t, *J* = 26.3 Hz), 134.2, 132.2, 131.5, 131.4, 130.2, 128.2, 125.9 (t, *J* = 5.6 Hz), 124.1, 117.4 (t, *J* = 245.9 Hz), 113.7, 28.9; ^19^F NMR (564 MHz, CDCl_3_) *δ*: −99.63. HRMS (ESI) ([M + Na]^+^) calcd for [C_16_H_12_F_2_N_2_NaO]^+^: 309.0810, found: 309.0814.

#### 3-(Difluoro(*p*-tolyl)methyl)-1-methylquinoxalin-2(1*H*)-one (3u)

Yellow solid. Mp 171.4–172.8 °C. ^1^H NMR (400 MHz, CDCl_3_) *δ*: 8.05–8.02 (m, 1H), 7.66–7.63 (m, 2H), 7.61 (s, 1H), 7.43–7.39 (m, 1H), 7.33–7.31 (m, 1H), 7.24–7.22 (m, 2H), 3.63 (s, 3H), 2.36 (s, 3H); ^13^C NMR (100 MHz, CDCl_3_) *δ*: 152.0, 150.7 (t, *J* = 28.2 Hz), 140.3, 134.2, 132.1, 132.0 (t, *J* = 26.4 Hz), 131.4 (double), 128.9, 125.8 (t, *J* = 5.6 Hz), 124.0, 117.5 (t, *J* = 245.2 Hz), 113.7, 28.9, 21.3; ^19^F NMR (100 MHz, CDCl_3_) *δ*: −99.21. HRMS (ESI) ([M + H]^+^) calcd for [C_17_H_14_F_2_N_2_NaO_2_]^+^: 339.0916, found: 339.0915.

#### 2-((4-Ethylphenyl)difluoromethyl)-1-methylquinoxalin-2(1*H*)-one (3v)

Yellow solid. Mp 171.4–172.8 °C. ^1^H NMR (400 MHz, CDCl_3_) *δ*: 8.05–8.03 (m, 1H), 7.66–7.62 (m, 3H), 7.43–7.39 (m, 1H), 7.33–7.31 (m, 1H), 7.26–7.24 (m, 2H), 3.64 (s, 3H), 2.66 (t, *J* = 7.6 Hz, 2H), 1.23 (t, *J* = 7.6 Hz, 3H); ^13^C NMR (100 MHz, CDCl_3_) *δ*: 152.1, 150.7 (t, *J* = 28.3 Hz), 146.5, 134.2, 132.2 (t, *J* = 26.3 Hz), 132.1, 131.5, 131.4, 127.7, 125.9 (t, *J* = 5.7 Hz), 124.0, 117.5 (t, *J* = 245.5 Hz), 113.7, 28.9, 28.7, 15.2; ^19^F NMR (376 MHz, CDCl_3_) *δ*: −99.13. HRMS (ESI) ([M + H]^+^) calcd for [C_18_H_17_F_2_N_2_O]^+^: 315.1303, found: 315.1304.

#### 3-((4-Butylphenyl)difluoromethyl)-1-methylquinoxalin-2(1*H*)-one (3w)

Yellow solid. Mp 106.7–107.4 °C. ^1^H NMR (600 MHz, CDCl_3_) *δ*: 8.04–8.02 (m, 1H), 7.65–7.63 (m, 3H), 7.42–7.39 (m, 1H), 7.32–7.30 (m, 1H), 7.23 (d, *J* = 7.8 Hz, 2H), 3.63 (s, 3H), 2.61 (t, *J* = 7.8 Hz, 2H), 1.61–1.56 (m, 2H), 1.37–1.31 (m, 2H), 0.91 (t, *J* = 8.4 Hz, 3H); ^13^C NMR (150 MHz, CDCl_3_) *δ*: 152.0, 150.7 (t, *J* = 28.2 Hz), 145.2, 134.2, 132.1 (t, *J* = 26.7 Hz), 132.08, 131.4, 131.3, 128.3, 125.8 (t, *J* = 5.4 Hz), 124.0, 117.5 (t, *J* = 245.6 Hz), 113.7, 35.4, 33.3, 28.9, 22.3, 11.9; ^19^F NMR (564 MHz, CDCl_3_) *δ*: −99.05. HRMS (ESI) ([M + H]^+^) calcd for [C_20_H_21_F_2_N_2_O]^+^: 343.1616, found: 343.1617.

#### ((4-(*tert*-Butyl)phenyl)difluoromethyl)-1-methylquinoxalin-2(1*H*)-one (3x)

Yellow solid. Mp 129.9–130.7 °C. ^1^H NMR (600 MHz, CDCl_3_) *δ*: 8.04–8.02 (m, 1H), 7.69–7.67 (m, 2H), 7.64–7.61 (m, 1H), 7.45–7.43 (m, 2H), 7.41–7.39 (m, 1H), 7.31 (d, *J* = 8.4 Hz, 1H), 3.63 (s, 3H), 1.30 (s, 9H); ^13^C NMR (150 MHz, CDCl_3_) *δ*: 153.3, 152.0, 150.7 (t, *J* = 28.2 Hz), 134.2, 132.1, 131.7 (t, *J* = 26.9 Hz), 131.5, 131.4, 125.7 (t, *J* = 5.6 Hz), 125.2, 124.0, 117.5 (t, *J* = 245.7 Hz), 113.7, 34.7, 31.2, 28.9; ^19^F NMR (564 MHz, CDCl_3_) *δ*: −99.08. HRMS (ESI) ([M + H]^+^) calcd for [C_20_H_21_F_2_N_2_O]^+^: 343.1616, found: 343.1615.

#### 3-([1,1′-Biphenyl]-4-yldifluoromethyl)-1-methylquinoxalin-2(1*H*)-one (3y)

Yellow solid. Mp 143.0–144.3 °C. ^1^H NMR (400 MHz, CDCl_3_) *δ*: 8.03–8.01 (m, 1H), 7.82–7.80 (m, 2H), 7.64–7.59 (m, 3H), 7.57–7.55 (m, 2H), 7.42–7.37 (m, 3H), 7.34–7.28 (m, 2H), 3.61 (s, 3H); ^13^C NMR (100 MHz, CDCl_3_) *δ*: 152.0, 150.4 (t, *J* = 28.3 Hz), 143.0, 140.2, 134.1, 133.7 (t, *J* = 26.8 Hz), 132.2, 131.4, 131.3, 128.7, 127.7, 127.0, 126.4 (t, *J* = 5.5 Hz), 124.0, 117.4 (t, *J* = 245.7 Hz), 113.7, 28.5; ^19^F NMR (376 MHz, CDCl_3_) *δ*: −99.24. HRMS (ESI) ([M + Na]^+^) calcd for [C_22_H_16_F_2_N_2_NaO]^+^: 385.1123, found: 385.1126.

#### 3-((4-Chlorophenyl)difluoromethyl)-1-methylquinoxalin-2(1*H*)-one (3z)

Yellow solid. Mp 231.1–231.4 °C. ^1^H NMR (600 MHz, CDCl_3_) *δ*: 8.03–8.02 (m, 1H), 7.69–7.67 (m, 2H), 7.66–7.65 (m, 1H), 7.44–7.41 (m, 1H), 7.40–7.39 (m, 2H), 7.34 (d, *J* = 8.4 Hz, 1H), 3.65 (s, 3H); ^13^C NMR (150 MHz, CDCl_3_) *δ*: 152.0, 150.1 (t, *J* = 28.1 Hz), 136.4, 134.2, 133.4 (t, *J* = 27.0 Hz), 132.4, 131.4 (double), 128.5, 127.5 (t, *J* = 5.6 Hz), 124.2, 117.0 (t, *J* = 246.0 Hz), 113.8, 29.0; ^19^F NMR (564 MHz, CDCl_3_) *δ*: −99.69. HRMS (ESI) ([M + Na]^+^) calcd for [C_16_H_11_ClF_2_N_2_NaO]^+^: 343.0420, found: 343.0423.

#### 3-((4-Bromophenyl)difluoromethyl)-1-methylquinoxalin-2(1*H*)-one (3aa)

Yellow solid. Mp 185.8–187.2 °C. ^1^H NMR (600 MHz, CDCl_3_) *δ*: 8.04–8.02 (m, 1H), 7.68–7.65 (m, 1H), 7.62–7.61 (m, 2H), 7.57–7.55 (m, 2H), 7.44–7.42 (m, 1H), 7.35–7.34 (m, 1H), 3.65 (s, 3H); ^13^C NMR (150 MHz, CDCl_3_) *δ*: 152.0, 150.2 (t, *J* = 28.1 Hz), 134.2, 133.9, 132.4, 131.5 (double), 131.4, 130.9, 128.8, 127.8 (t, *J* = 5.6 Hz), 124.8, 117.1 (t, *J* = 246.0 Hz), 113.8, 29.0; ^19^F NMR (564 MHz, CDCl_3_) *δ*: −99.92. HRMS (ESI) ([M + Na]^+^) calcd for [C_16_H_11_BrF_2_N_2_NaO]^+^: 386.9915, found: 386.9918.

#### 3-(Difluoro(*o*-tolyl)methyl)-1-methylquinoxalin-2(1*H*)-one (3ab)

Yellow solid. Mp 154.2–155.3 °C. ^1^H NMR (600 MHz, CDCl_3_) *δ*: 8.01–7.99 (m, 1H), 7.86–7.85 (m, 1H), 7.66–7.64 (m, 1H), 7.42–7.39 (m, 1H), 7.34–7.29 (m, 3H), 7.17–7.16 (m, 1H), 3.64 (s, 3H), 2.38 (s, 3H); ^13^C NMR (150 MHz, CDCl_3_) *δ*: 152.1, 150.2 (t, *J* = 28.4 Hz), 136.2 (t, *J* = 3.0 Hz), 134.2, 132.7 (t, *J* = 24.2 Hz), 132.2, 131.5, 131.4, 131.2, 130.1, 127.5 (t, *J* = 8.1 Hz), 125.5, 124.0, 118.2 (t, *J* = 245.6 Hz), 113.7, 28.9, 20.2; ^19^F NMR (564 MHz, CDCl_3_) *δ*: −97.61. HRMS (ESI) ([M + H]^+^) calcd for [C_17_H_15_F_2_N_2_O]^+^: 301.1147, found: 301.1146.

#### 3-(Difluoro(2-methoxyphenyl)methyl)-1-methylquinoxalin-2(1*H*)-one (3ac)

Yellow solid. Mp 151.4–152.7 °C. ^1^H NMR (600 MHz, CDCl_3_) *δ*: 8.03–8.01 (m, 1H), 7.88–7.87 (m, 1H), 7.64–7.61 (m, 1H), 7.41–7.37 (m, 2H), 7.32 (d, *J* = 8.4 Hz, 1H), 7.10 (t, *J* = 7.8 Hz, 1H), 6.86 (d, *J* = 8.4 Hz, 1H), 3.60 (s, 3H), 3.56 (s, 3H); ^13^C NMR (150 MHz, CDCl_3_) *δ*: 156.4 (t, *J* = 4.7 Hz), 152.2, 150.7 (t, *J* = 26.7 Hz), 133.9, 131.7, 131.5, 131.4, 131.0, 127.1 (t, *J* = 7.4 Hz), 123.8, 123.7 (t, *J* = 24.8 Hz), 120.6, 116.0 (t, *J* = 242.6 Hz), 113.6, 113.4, 55.7, 28.7; ^19^F NMR (564 MHz, CDCl_3_) *δ*: −98.27. HRMS (ESI) ([M + H]^+^) calcd for [C_17_H_15_F_2_N_2_O_2_]^+^: 317.1096, found: 317.1097.

#### 3-(Difluoro(*m*-tolyl)methyl)-1-methylquinoxalin-2(1*H*)-one (3ad)

Yellow solid. Mp 143.4–144.3 °C. ^1^H NMR (600 MHz, CDCl_3_) *δ*: 8.03–8.01 (m, 1H), 7.64–7.61 (m, 1H), 7.54–7.52 (m, 2H), 7.41–7.38 (m, 1H), 7.31–7.29 (m, 2H), 7.23–7.22 (m, 1H), 3.61 (s, 3H), 2.36 (s, 3H); ^13^C NMR (150 MHz, CDCl_3_) *δ*: 151.9, 150.5 (t, *J* = 28.1 Hz), 138.0, 134.7 (t, *J* = 26.1 Hz), 134.1, 132.1, 131.3, 131.2, 130.9, 128.1, 126.2 (t, *J* = 5.4 Hz), 124.0, 123.0 (t, *J* = 5.6 Hz), 117.3 (t, *J* = 245.6 Hz), 113.7, 28.8, 21.3; ^19^F NMR (564 MHz, CDCl_3_) *δ*: −99.20. HRMS (ESI) ([M + H]^+^) calcd for [C_17_H_15_F_2_N_2_O]^+^: 301.1147, found: 301.1149.

#### 3-((2,4-Dimethoxyphenyl)difluoromethyl)-1-methylquinoxalin-2(1*H*)-one (3ae)

Yellow solid. Mp 191.3–192.4 °C. ^1^H NMR (600 MHz, CDCl_3_) *δ*: 8.03–8.02 (m, 1H), 7.80–7.79 (m, 1H), 7.64–7.61 (m, 1H), 7.41–7.38 (m, 1H), 7.33–7.32 (m, 1H), 6.61 (dd, *J*_1_ = 9.0 Hz, *J*_2_ = 2.4 Hz, 1H), 6.40–6.39 (m, 1H), 3.80 (s, 3H), 3.62 (s, 3H), 3.54 (s, 3H); ^13^C NMR (150 MHz, CDCl_3_) *δ*: 162.3, 157.7 (t, *J* = 4.7 Hz), 152.2, 150.8 (t, *J* = 27.0 Hz), 133.9, 131.6, 131.4, 131.0, 128.3 (t, *J* = 7.2 Hz), 123.8, 116.2 (t, *J* = 25.0 Hz), 116.19 (t, *J* = 242.0 Hz), 113.6, 104.4, 98.8, 55.6, 55.2, 28.7; ^19^F NMR (564 MHz, CDCl_3_) *δ*: −97.04. HRMS (ESI) ([M + Na]^+^) calcd for [C_18_H_16_F_2_N_2_NaO_3_]^+^: 369.1021, found: 369.1025.

#### 3-((3,4-Dimethylphenyl)difluoromethyl)-1-methylquinoxalin-2(1*H*)-one (3af)

Yellow solid. Mp 206.7–207.1 °C. ^1^H NMR (600 MHz, CDCl_3_) *δ*: 8.02–8.01 (m, 1H), 7.63–7.60 (m, 1H), 7.46–7.45 (m, 2H), 7.40–7.37 (m, 1H), 7.30–7.28 (m, 1H), 7.17–7.16 (m, 1H), 3.60 (s, 3H), 2.25 (s, 3H), 2.24 (s, 3H); ^13^C NMR (150 MHz, CDCl_3_) *δ*: 151.9, 150.6 (t, *J* = 28.2 Hz), 138.9, 136.5, 134.1, 132.2 (t, *J* = 26.1 Hz), 132.0, 131.3, 131.2, 129.4, 126.6 (t, *J* = 5.4 Hz), 123.9, 123.2 (t, *J* = 5.6 Hz), 117.4 (t, *J* = 245.4 Hz), 113.7, 28.8, 19.7, 19.6; ^19^F NMR (564 MHz, CDCl_3_) *δ*: −98.82. HRMS (ESI) ([M + H]^+^) calcd for [C_18_H_17_F_2_N_2_O]^+^: 315.1303, found: 315.1304.

#### 3-((3,5-Dimethylphenyl)difluoromethyl)-1-methylquinoxalin-2(1*H*)-one (3ag)

Yellow solid. Mp 186.7–188.4 °C. ^1^H NMR (600 MHz, CDCl_3_) *δ*: 8.06–8.05 (m, 1H), 7.66–7.64 (m, 1H), 7.43–7.41 (m, 1H), 7.34–7.32 (m, 3H), 7.05 (s, 1H), 3.64 (s, 3H), 2.33 (s, 6H); ^13^C NMR (150 MHz, CDCl_3_) *δ*: 152.1, 150.7 (t, *J* = 28.1 Hz), 137.9, 134.7 (t, *J* = 25.7 Hz), 134.2, 132.1, 131.9, 131.4 (double), 124.0, 123.4 (t, *J* = 5.6 Hz), 117.44, 117.4 (t, *J* = 245.0 Hz), 113.7, 28.9, 21.3; ^19^F NMR (564 MHz, CDCl_3_) *δ*: −99.00. HRMS (ESI) ([M + Na]^+^) calcd for [C_18_H_16_F_2_N_2_NaO]^+^: 337.1123, found: 337.1127.

#### 3-(Difluoro(thiophen-2-yl)methyl)-1-methylquinoxalin-2(1*H*)-one (3ah)

Yellow solid. Mp 121.4–122.8 °C. ^1^H NMR (400 MHz, CDCl_3_) *δ*: 8.01–7.99 (m, 1H), 7.79–7.78 (m, 1H), 7.67–7.63 (m, 1H), 7.42–7.37 (m, 2H), 7.35–7.31 (m, 2H), 3.67 (s, 3H); ^13^C NMR (100 MHz, CDCl_3_) *δ*: 152.0, 150.2 (t, *J* = 28.1 Hz), 136.2 (t, *J* = 29.4 Hz), 134.2, 132.2, 131.4, 131.3, 126.0, 125.9 (t, *J* = 6.6 Hz), 125.8, 124.1, 115.9 (t, *J* = 244.3 Hz), 113.7, 29.0; ^19^F NMR (376 MHz, CDCl_3_) *δ*: −94.82. HRMS (ESI) ([M + Na]^+^) calcd for [C_14_H_10_F_2_N_2_NaOS]^+^: 315.0374, found: 315.0371.

#### 3-(Difluoro(naphthalen-1-yl)methyl)-1-methylquinoxalin-2(1*H*)-one (3ai)

Yellow solid. Mp 148.7–149.5 °C. ^1^H NMR (600 MHz, CDCl_3_) *δ*: 8.36–8.35 (m, 1H), 8.23–8.22 (m, 1H), 8.09–8.07 (m, 1H), 7.94–7.93 (m, 1H), 7.85–7.83 (m, 1H), 7.63–7.59 (m, 2H), 7.43–7.39 (m, 3H), 7.27–7.25 (m, 1H), 3.55 (s, 3H); ^13^C NMR (150 MHz, CDCl_3_) *δ*: 152.0, 150.5 (t, *J* = 28.1 Hz), 134.3, 133.8, 132.3, 131.4, 131.3 (double), 129.8 (t, *J* = 24.6 Hz), 129.5, 128.8, 126.7, 126.6 (t, *J* = 8.8 Hz), 125.6, 125.0, 124.7, 124.0, 118.1 (t, *J* = 246.8 Hz), 113.7, 28.8; ^19^F NMR (564 MHz, CDCl_3_) *δ*: −96.21. HRMS (ESI) ([M + Na]^+^) calcd for C_20_H_14_F_2_N_2_NaO: 359.0966, found: 359.0967.

#### 2-(Difluoro(4-methoxyphenyl)methyl)quinoxaline (5a)

Yellow solid. Mp 121.5–123.8 °C. ^1^H NMR (600 MHz, CDCl_3_) *δ*: 9.19 (s, 1H), 8.17–8.15 (m, 2H), 7.85–7.81 (m, 2H), 7.59 (d, *J* = 9.0 Hz, 2H), 6.96 (d, *J* = 8.4 Hz, 2H), 3.83 (s, 3H); ^13^C NMR (150 MHz, CDCl_3_) *δ*: 161.1, 150.3 (t, *J* = 31.5 Hz), 142.7, 142.0 (t, *J* = 4.5 Hz), 141.1, 131.2, 130.8, 129.9, 129.3, 127.8 (t, *J* = 27.3 Hz), 127.5 (t, *J* = 5.6 Hz), 118.6 (t, *J* = 242.7 Hz), 113.9, 55.4; ^19^F NMR (564 MHz, CDCl_3_) *δ*: −93.48. HRMS (ESI) ([M + Na]^+^) calcd for [C_16_H_12_F_2_N_2_NaO]^+^: 309.0810, found: 309.0809.

## Conflicts of interest

There are no conflicts to declare

## Supplementary Material

RA-010-D0RA02059A-s001

RA-010-D0RA02059A-s002
