# Supplementary material for: Photoinitiated decarboxylative C3-difluoroarylmethylation of quinoxalin-2(1H)-ones with potassium 2,2-difluoro-2-arylacetates in water
Source: RSC Adv. 2020 Mar 12;10(18):10559–68. doi: 10.1039/d0ra02059a (PMC9050393; doi:10.1039/d0ra02059a)
Supplement: RA-010-D0RA02059A-s001 [file RA-010-D0RA02059A-s001.pdf]

Electronic Supplementary Material (ESI) for RSC Advances.
This journal is © The Royal Society of Chemistry 2020

Supporting Information

Photoinitiated Decarboxylative C3-difluoroarylmethylation of Quinoxalin-2(1H)-ones with Potassium 2,2-Difluoro-2-arylacetates in water

Yanhui Gao,[a] Lulu Zhao,[a] Tianyi Xiang,[c] Pinhua Li*,[a] and Lei Wang*,[a,b]

[a] Key Laboratory of Green and Precise Synthetic Chemistry and Applications, Ministry of Education; Department of chemistry, Huaibei Normal University, Huaibei, Anhui 235000, P. R. China; E-mail: pphuali@126.com; leiwang@chnu.edu.cn

[b] Advanced Research Institute and Department of Chemistry, Taizhou University, Taizhou, Zhejiang, 318000, P. R. China

[c] College of Pharmacy, Shenyang Pharmaceutical University, Shenyang, 110016, P. R. China

Table of Contents for Supporting Information

1. General considerations……………………………..…………………..……..………2
2. Representative procedure for the model reaction……..……….......…………….…2
3. Mechanism investigation............…………………...……….…...…………….……..3
4. X-ray single crystal structure of **3i**………..………………...……..... …...………...…3
5. ^{1}H, ^{13}C and ^{19}F NMR spectra of the products…..…..….……………..……...…...4

1. General considerations

All ^{1}H NMR and ^{13}C NMR spectra were recorded on 400 MHz or 600 MHz Bruker FT-NMR spectrometer (400 MHz or 100 MHz and 600 MHz or 150 MHz, respectively), and ^{19}F NMR spectra were recorded on a 400 MHz Bruker FT-NMR spectrometer (376 MHz). All chemical shifts are given as δ value (ppm) with reference to tetramethyl silane (TMS) as an internal standard. The peak patterns are indicated as follows: s, singlet; d, doublet; t, triplet; m, multiplet; q, quartet. The coupling constants, *J*, are reported in Hertz (Hz). High resolution mass spectroscopy data of the product were collected on an Agilent Technologies 6540 UHD Accurate-Mass Q-TOF LC/MS (ESI). Melting points (uncorrected) were obtained on WRS-1B digital melting point apparatus. The procedures for the synthesis of quinoxalin-2(1*H*)-ones are according to the reported method (See: S. Liu, Y. Huang, F.-L. Qing and X.-H. Xu. *Org. Lett.*, 2018, **20**, 5497). The preparation of potassium 2,2-difluoro-2-(4-methoxyphenyl)acetats is according to the reported procedure (See: W. Wan, G. Ma, J. Li, Y. Chen, Q. Hu, M. Li, H. Jiang, H. Deng and J. Hao. *Chem. Commun.*, 2016, **52**, 1598).

2. Representative procedure for the model reaction

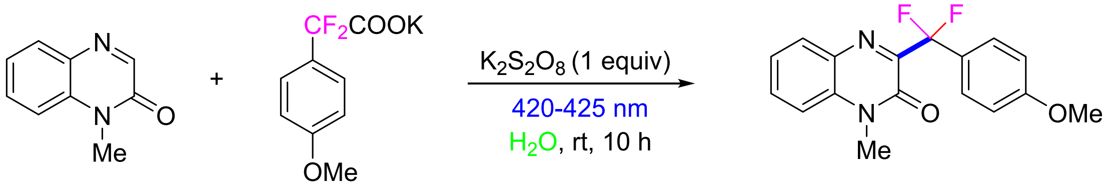

A 5 mL oven-dried reaction vessel equipped with a magnetic stirrer bar was charged with *N*-methyl-quinoxalin-2(1*H*)-one (**1a**, 0.10 mmol), potassium 2,2-difluoro-2-(4-methoxyphenyl)acetate (**2a**, 0.20 mmol), $K_2S_2O_8$ (0.10 mmol) and H_2O (3.0 mL). The reaction vessel was exposed to a blue LED (420–425 nm, 1.5 W) irradiation at room temperature in air with stirring for 10 h. After completion of the reaction, the mixture was extracted with ethyl acetate and concentrated to yield the crude product, which was further purified by flash chromatography (silica gel, petroleum ether/ethyl acetate = 20:1 to 9:1) to give the desired product **3a**.

3. Mechanism investigation

A 5 mL oven-dried reaction vessel equipped with a magnetic stirrer bar was charged with *N*-methylquinoxalin-2(1*H*)-one (**1a**, 0.10 mmol), potassium 2,2-difluoro-2-phenylacetate (**2t**, 0.20 mmol), $K_2S_2O_8$ (1.0 eq.), TEMPO (2.5 eq.) and H_2O (3.0 mL), the reaction vessel was exposed to blue LED (420-425 nm, 1.5 W) irradiation in air at room temperature with stirring for 10 h. The reaction was completely inhibited, and an adduct (**8**) of TEMPO with a free radical was detected by HPLC/HRMS analysis of reaction mixture (Figure S1).

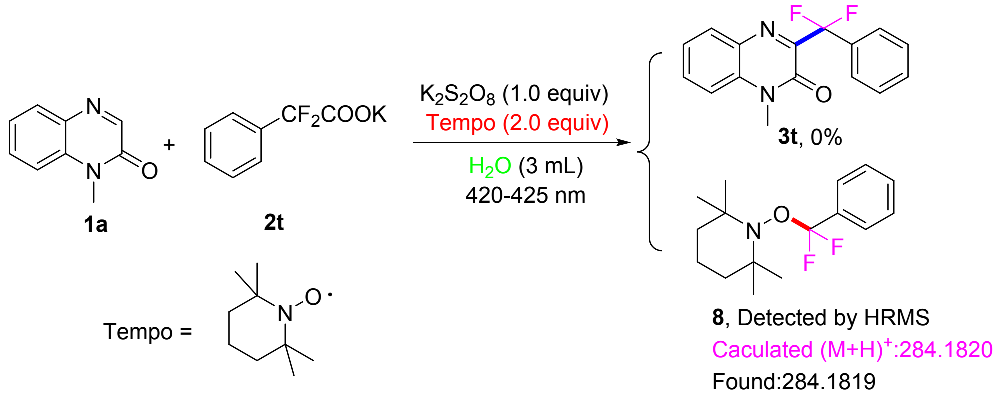

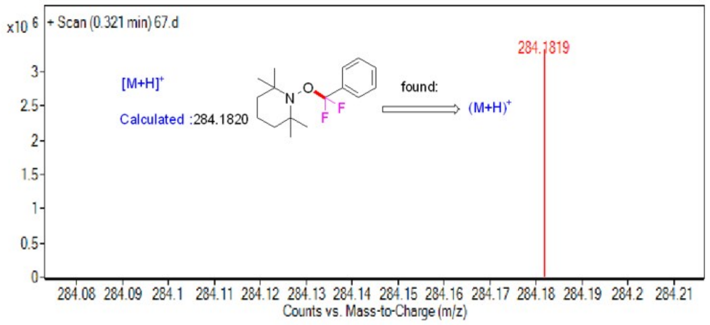

Figure S1. Analysis of reaction mixture by HRMS analysis

4. X-ray single crystal structure 3i (CCDC Number: 1961424)

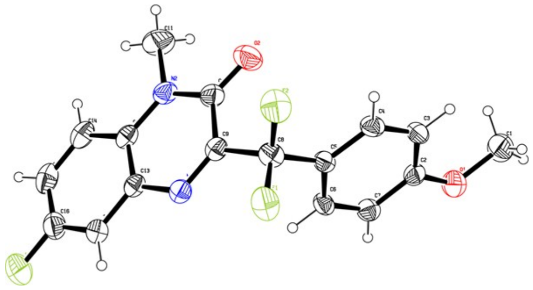

5. ^{1}H, ^{13}C and ^{19}F NMR spectra of the products

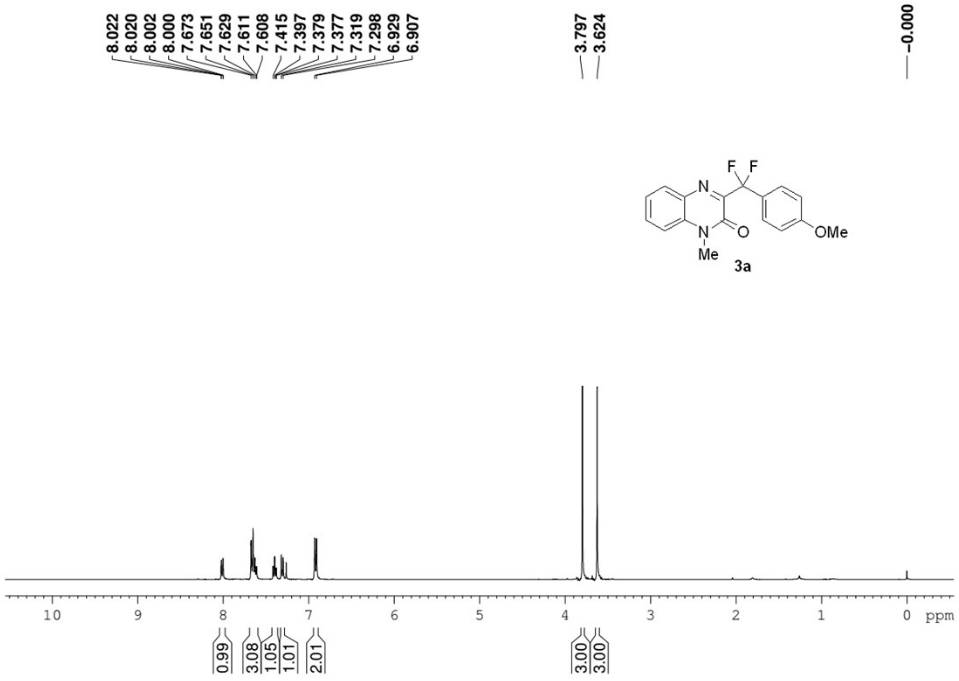

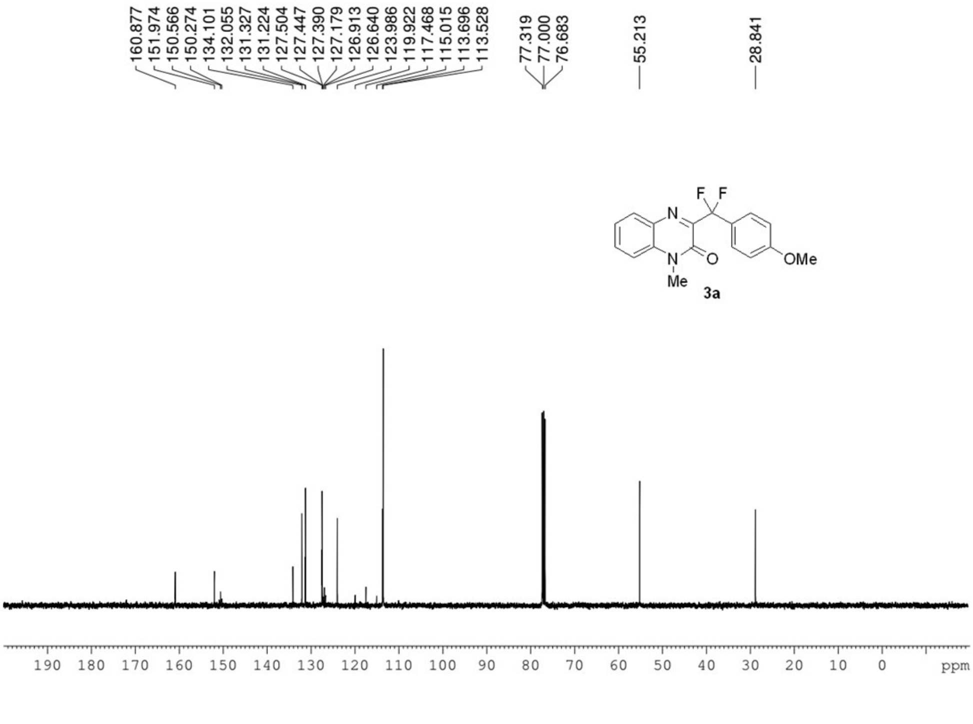

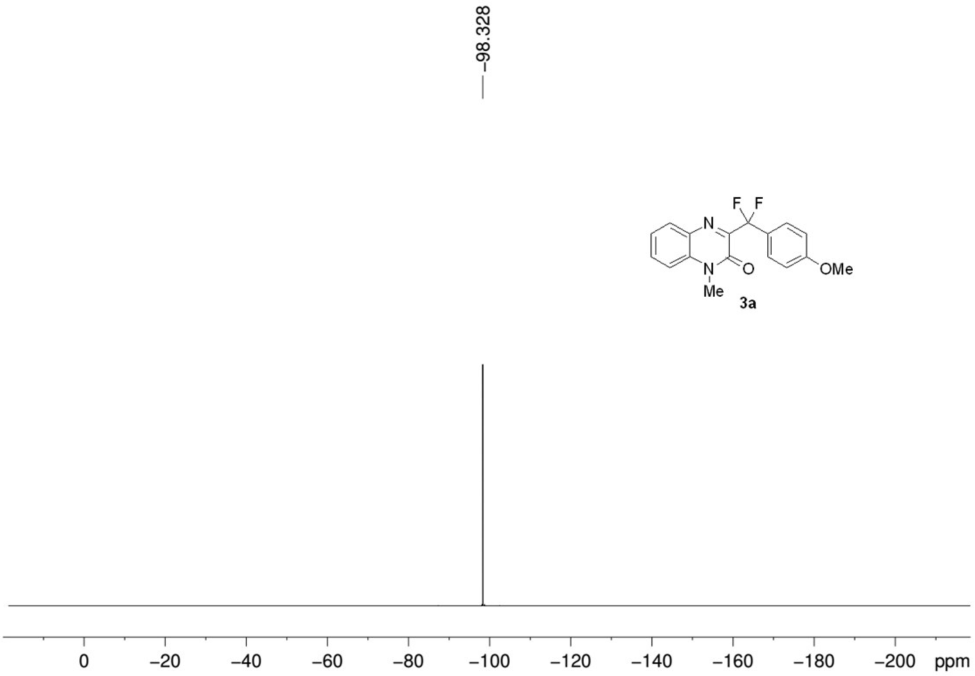

−98.328
F F
N
N
O
OMe
Me
3a
0 −20 −40 −60 −80 −100 −120 −140 −160 −180 −200 ppm

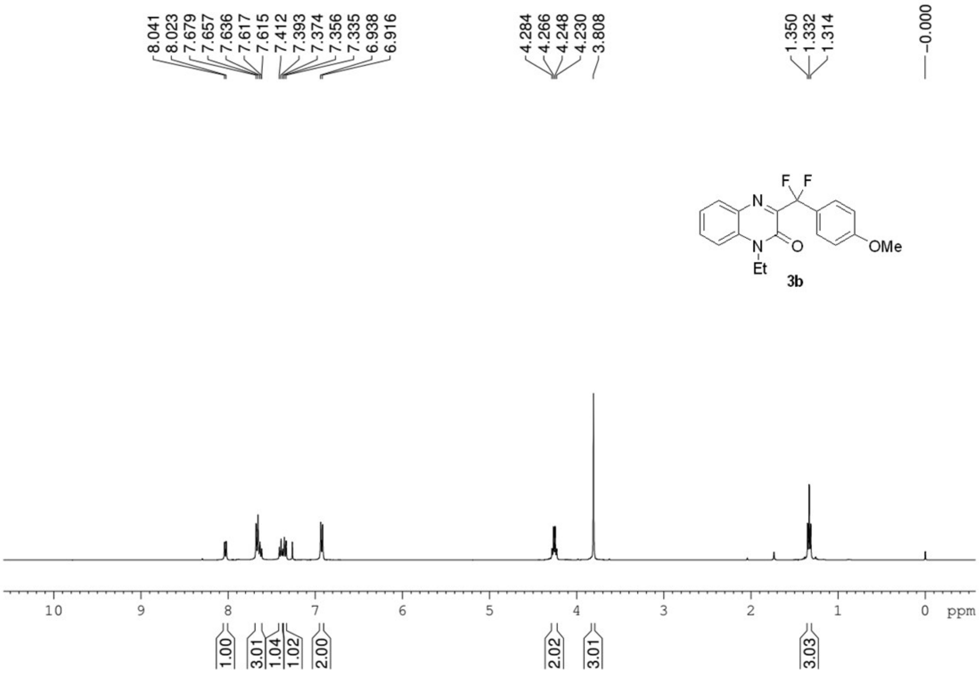

8.041 8.023 7.679 7.657 7.636 7.617 7.615 7.412 7.393 7.374 7.356 7.335 6.938 6.916
4.284 4.266 4.248 4.230 3.808
1.350 1.332 1.314
−0.000
F F
N
N
O
OMe
Et
3b
10 9 8 7 6 5 4 3 2 1 0 ppm
1.00 3.01 1.04 1.02 2.00 2.02 3.01 3.03

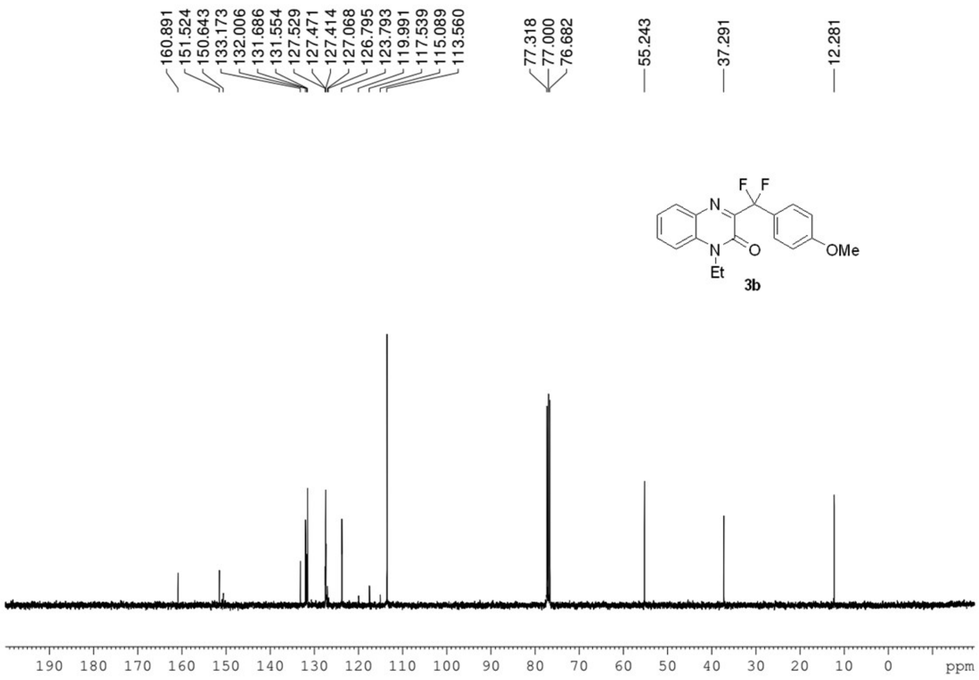
160.891
151.524
150.643
133.173
132.006
131.686
131.554
127.529
127.471
127.414
127.068
126.795
123.793
119.991
117.539
115.089
113.560
77.318
77.000
76.682
55.243
37.291
12.281
OMe
3b
190 180 170 160 150 140 130 120 110 100 90 80 70 60 50 40 30 20 10 0 ppm

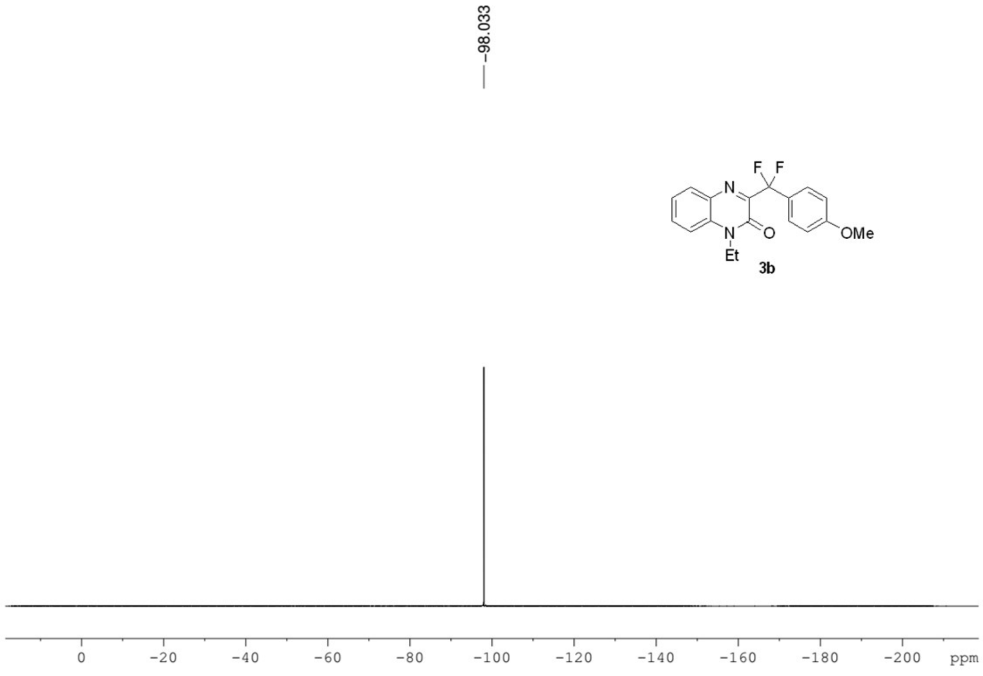
-98.033
OMe
3b
0 -20 -40 -60 -80 -100 -120 -140 -160 -180 -200 ppm

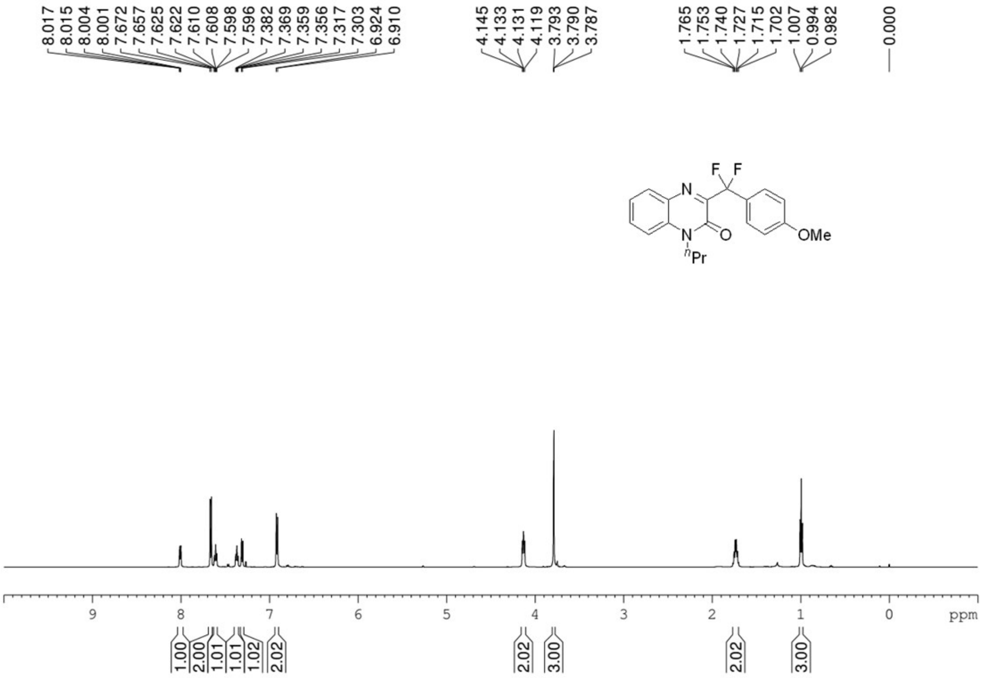
8.017 8.015 8.004 8.001 7.672 7.657 7.625 7.622 7.610 7.608 7.598 7.596 7.382 7.369 7.359 7.356 7.317 7.303 6.924 6.910
4.145 4.133 4.131 4.119 3.793 3.790 3.787
1.765 1.753 1.740 1.727 1.715 1.702 1.007 0.994 0.982
0.000
F F
N
N
O
OMe
^{n}Pr
9 8 7 6 5 4 3 2 1 0 ppm
1.00 2.00 1.01 1.01 1.02 2.02 2.02 3.00 2.02 3.00

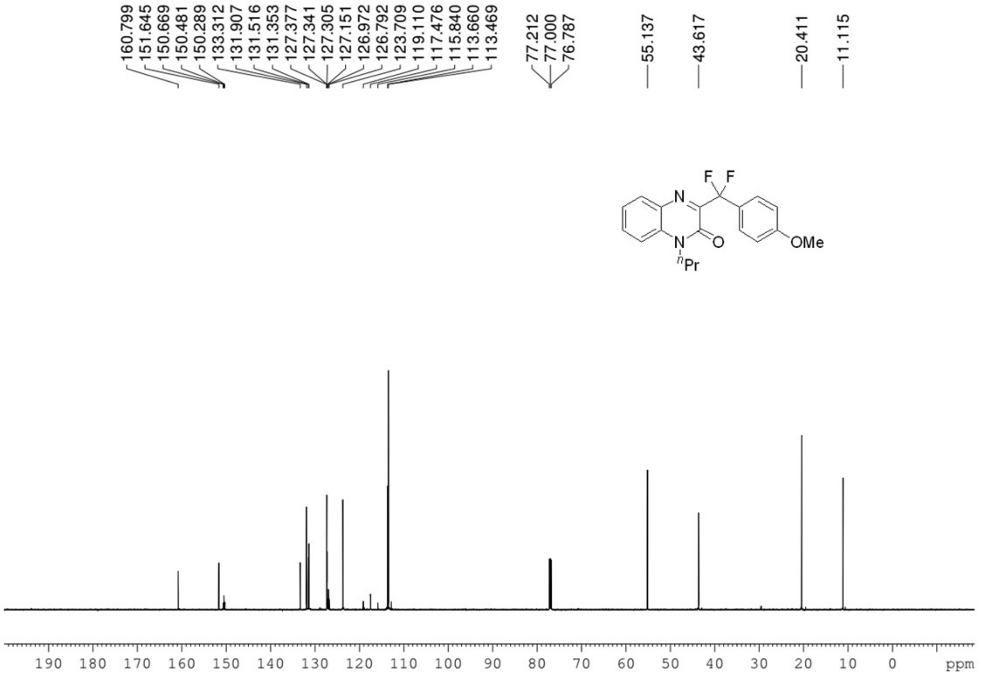
160.799 151.645 150.669 150.481 150.289 133.312 131.907 131.516 131.353 127.377 127.341 127.305 127.151 126.972 126.792 123.709 119.110 117.476 115.840 113.660 113.469
77.212 77.000 76.787
55.137
43.617
20.411
11.115
F F
N
N
O
OMe
^{n}Pr
190 180 170 160 150 140 130 120 110 100 90 80 70 60 50 40 30 20 10 0 ppm

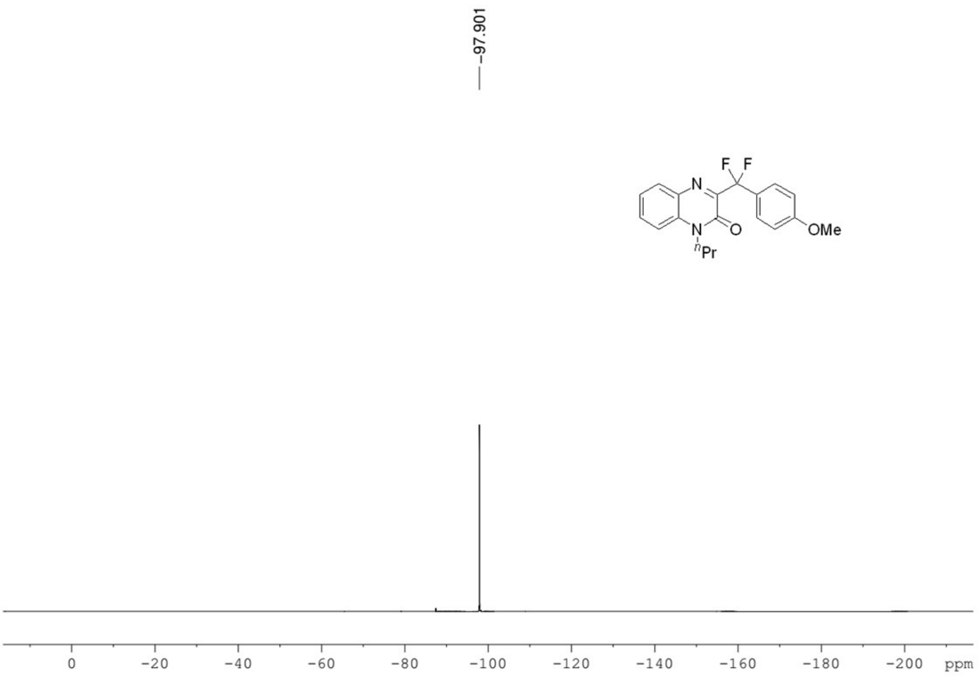

-97.901
F F
N
N
O
nPr
OMe
0 -20 -40 -60 -80 -100 -120 -140 -160 -180 -200 ppm

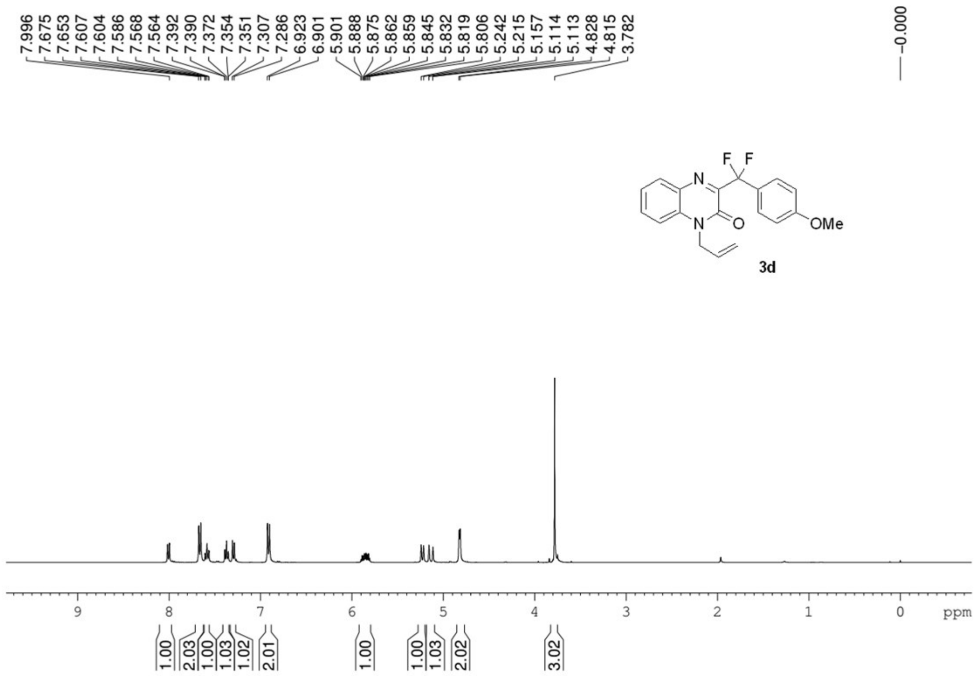

7.996 7.675 7.653 7.607 7.604 7.586 7.568 7.564 7.392 7.390 7.372 7.354 7.351 7.307 7.286 6.923 6.901 5.901 5.888 5.875 5.862 5.859 5.845 5.832 5.819 5.806 5.242 5.215 5.157 5.114 5.113 4.828 4.815 3.782
-0.000
F F
N
N
O
OMe
3d
9 8 7 6 5 4 3 2 1 0 ppm
1.00 2.03 1.00 1.03 1.02 2.01 1.00 1.00 1.03 2.02 3.02

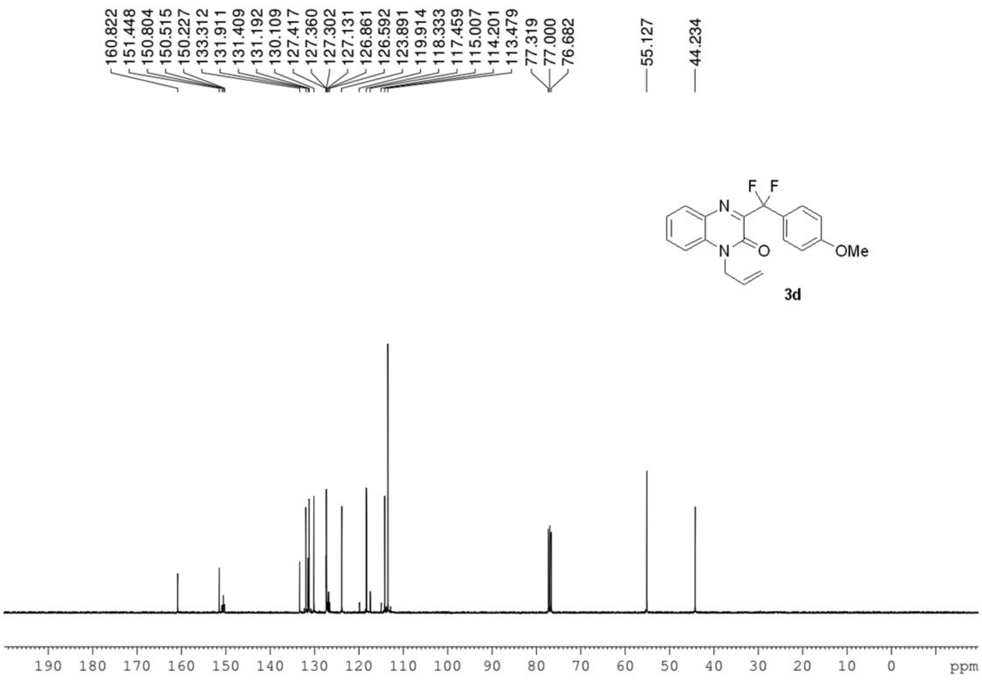
160.822
151.448
150.804
150.515
150.227
133.312
131.911
131.409
131.192
130.109
127.417
127.360
127.302
127.131
126.861
126.592
123.891
119.914
118.333
117.459
115.007
114.201
113.479
77.319
77.000
76.682
55.127
44.234
F F
N
N
O
OMe
3d
190 180 170 160 150 140 130 120 110 100 90 80 70 60 50 40 30 20 10 0 ppm

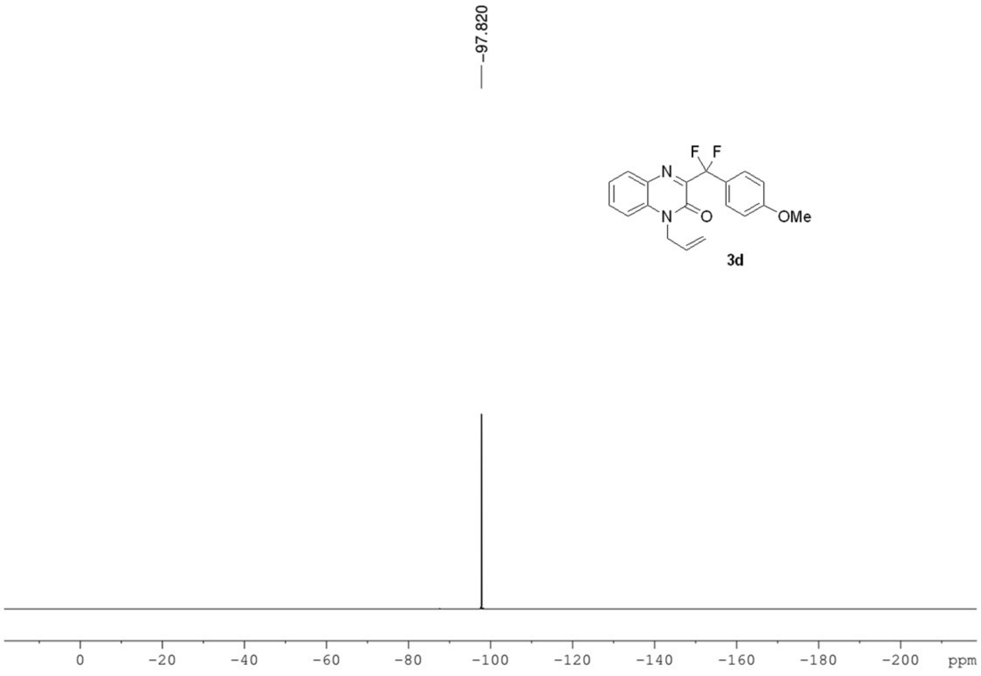
-97.820
F F
N
N
O
OMe
3d
0 -20 -40 -60 -80 -100 -120 -140 -160 -180 -200 ppm

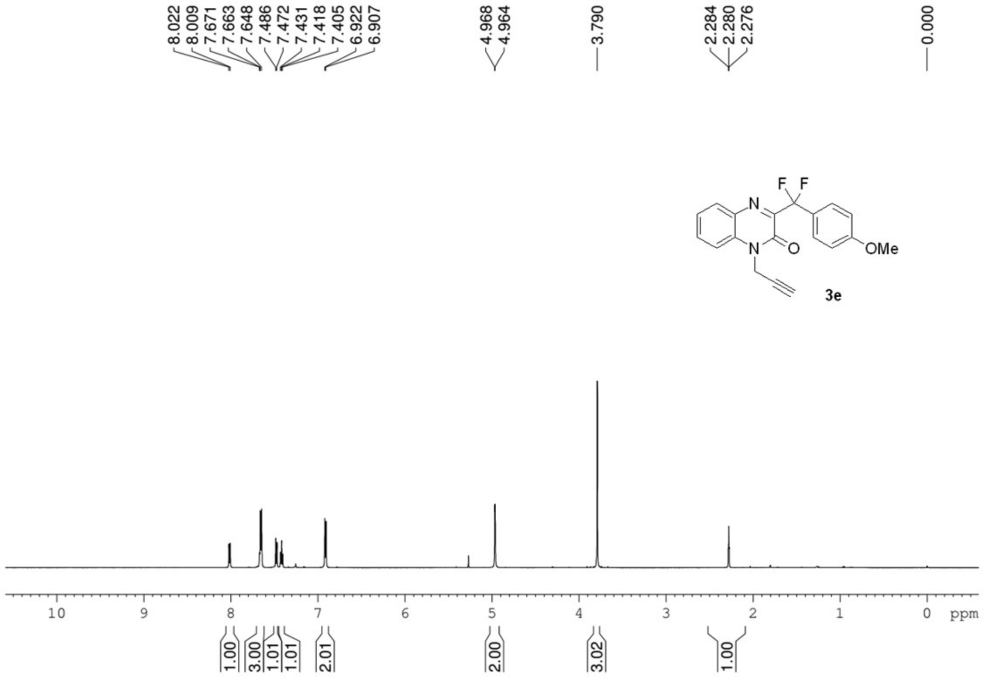

8.022
8.009
7.671
7.663
7.648
7.486
7.472
7.431
7.418
7.405
6.922
6.907
4.968
4.964
3.790
2.284
2.280
2.276
0.000
F
F
N
N
O
OMe
3e
10
9
8
7
6
5
4
3
2
1
0
ppm
1.00
3.00
1.01
1.01
2.01
2.00
3.02
1.00

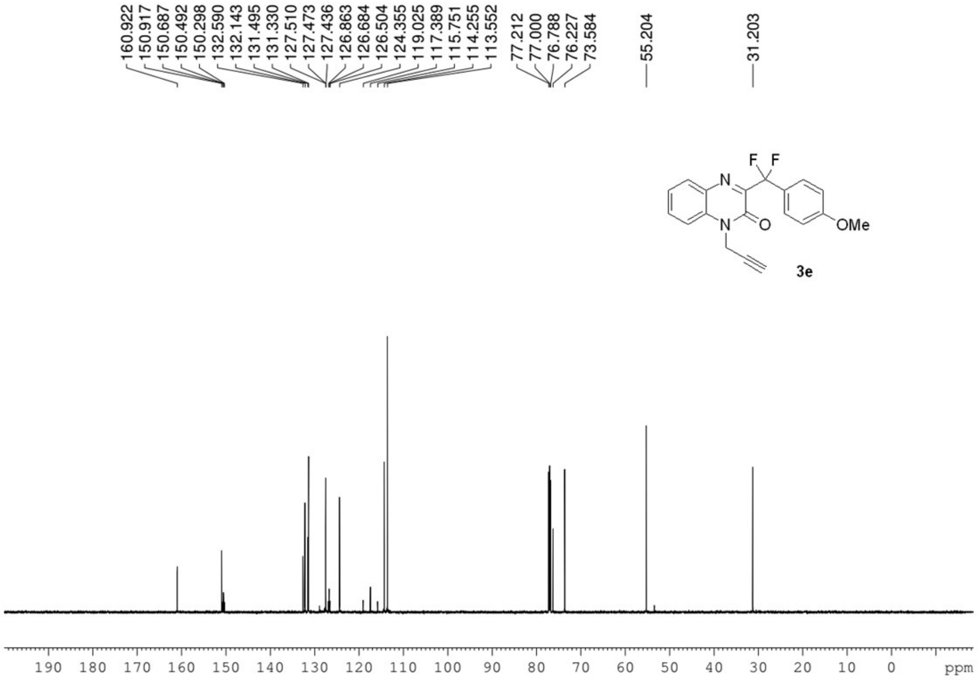

160.922
150.917
150.687
150.492
150.298
132.590
132.143
131.495
131.330
127.510
127.473
127.436
126.863
126.684
126.504
124.355
119.025
117.389
115.751
114.255
113.552
77.212
77.000
76.788
76.227
73.584
55.204
31.203
F
F
N
N
O
OMe
3e
190
180
170
160
150
140
130
120
110
100
90
80
70
60
50
40
30
20
10
0
ppm

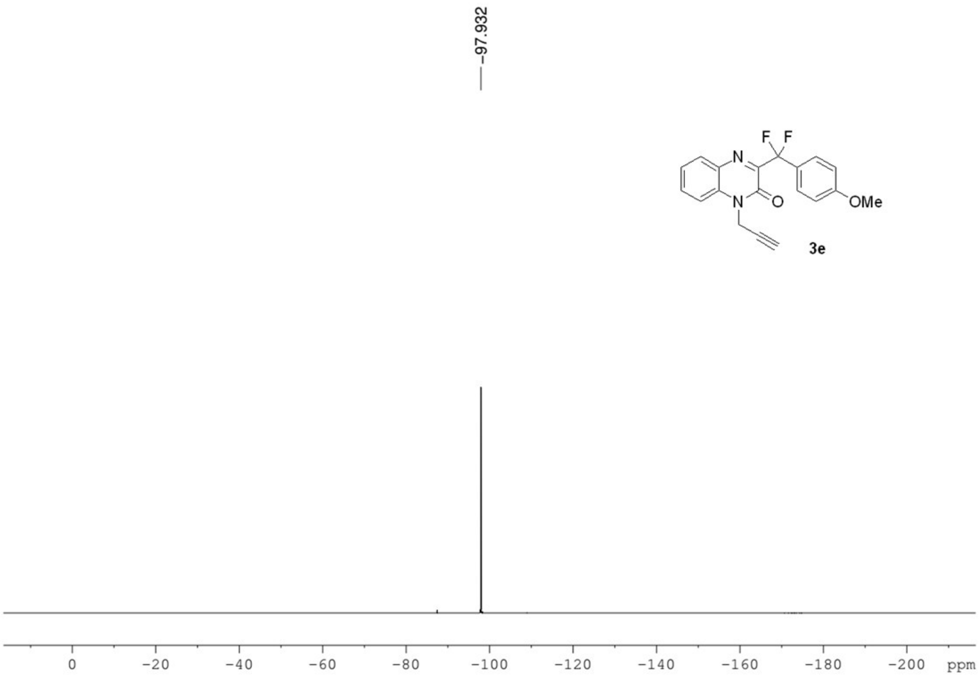
-97.932
F F
N
O
N
OMe
3e
0 -20 -40 -60 -80 -100 -120 -140 -160 -180 -200 ppm

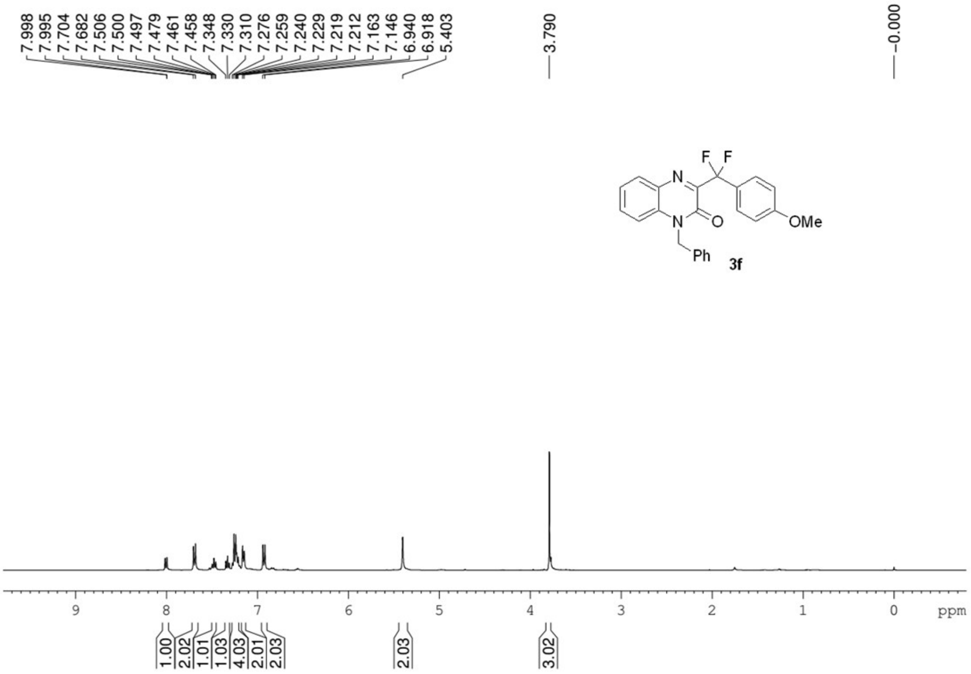
7.998 7.995 7.704 7.682 7.506 7.500 7.497 7.479 7.461 7.458 7.348 7.330 7.310 7.276 7.259 7.240 7.229 7.219 7.212 7.163 7.146 6.940 6.918 5.403
3.790
0.000
F F
N
O
N
OMe
Ph
3f
9 8 7 6 5 4 3 2 1 0 ppm
1.00 2.02 1.01 1.03 4.03 2.01 2.03
2.03
3.02

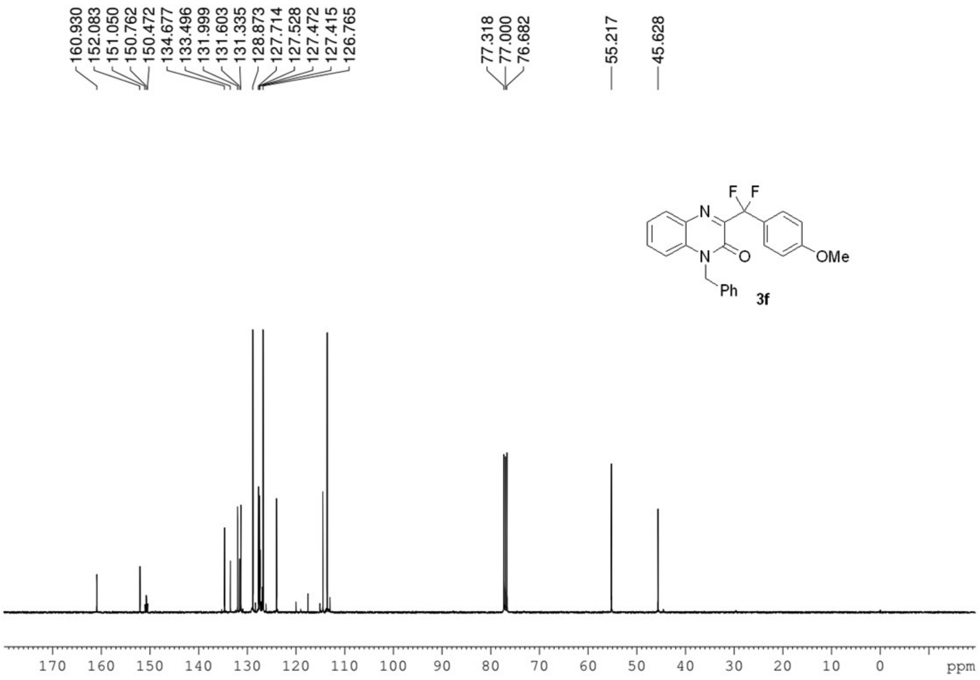
160.930
152.083
151.050
150.762
150.472
134.677
133.496
131.999
131.603
131.335
128.873
127.714
127.528
127.472
127.415
126.765
77.318
77.000
76.682
55.217
45.628
F F
N
N
O
OMe
Ph
3f
ppm

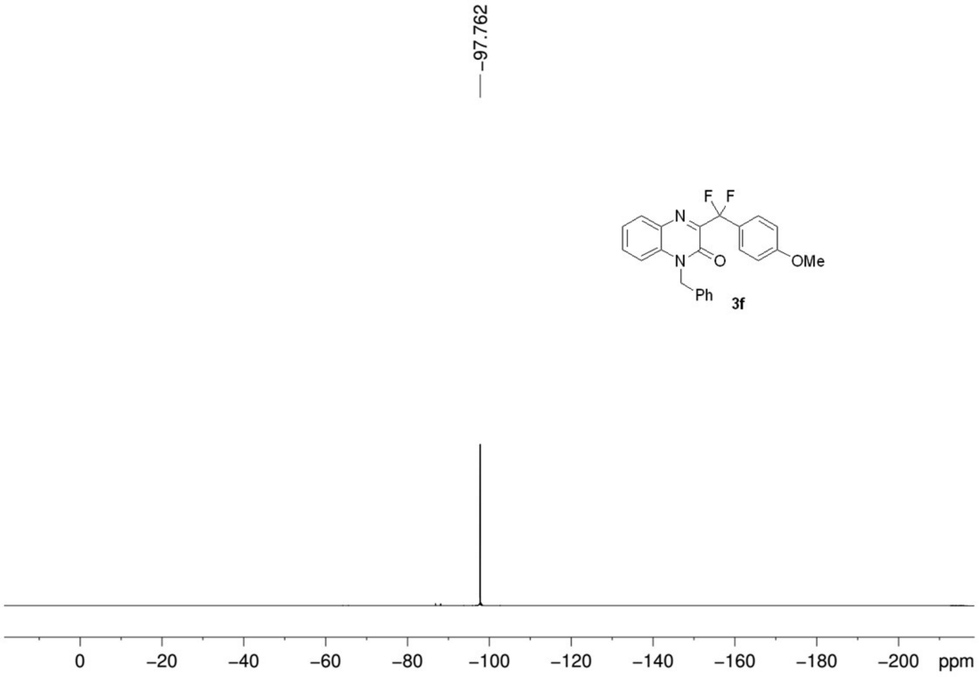
-97.762
F F
N
N
O
OMe
Ph
3f
ppm

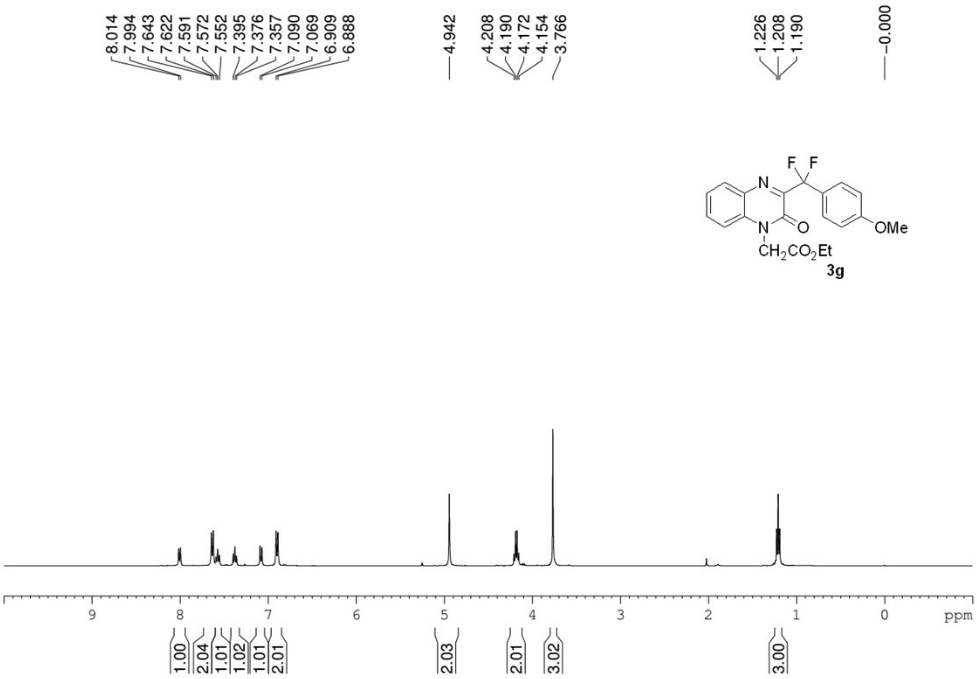

8.014
7.994
7.643
7.622
7.591
7.572
7.552
7.395
7.376
7.357
7.090
7.069
6.909
6.888
4.942
4.208
4.190
4.172
4.154
3.766
1.226
1.208
1.190
-0.000
F F
N
N
O
OMe
CH2CO2Et
3g
9 8 7 6 5 4 3 2 1 0 ppm
1.00
2.04
1.01
1.02
1.01
2.01
2.03
2.01
3.02
3.00

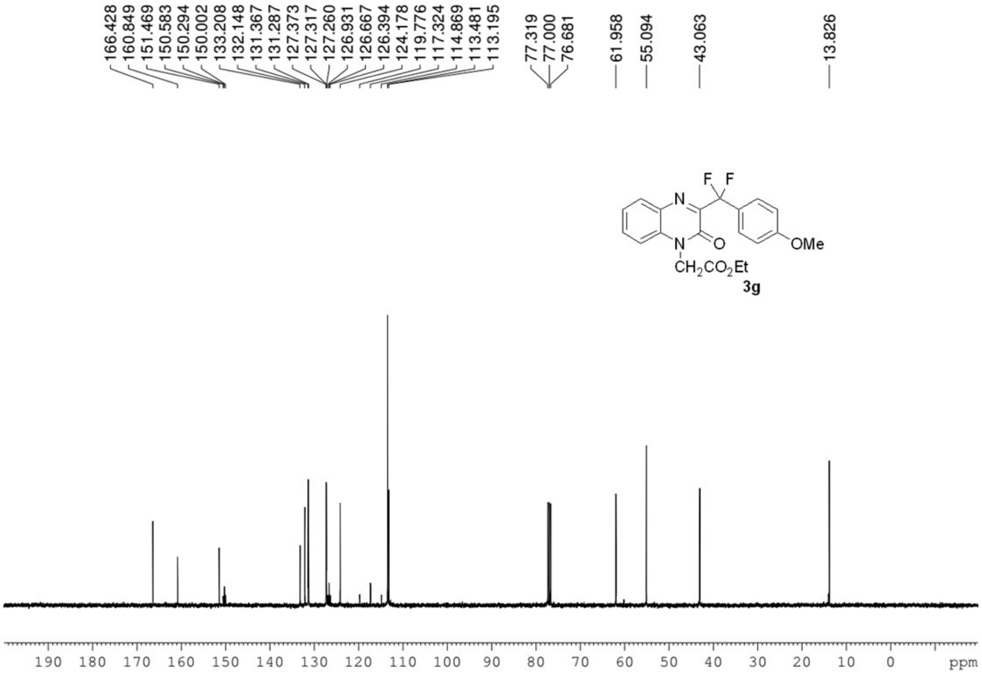

166.428
160.849
151.469
150.583
150.294
150.002
133.208
132.148
131.367
131.287
127.373
127.317
127.260
126.931
126.667
126.394
124.178
119.776
117.324
114.869
113.481
113.195
77.319
77.000
76.681
61.958
55.094
43.063
13.826
F F
N
N
O
OMe
CH2CO2Et
3g
190 180 170 160 150 140 130 120 110 100 90 80 70 60 50 40 30 20 10 0 ppm

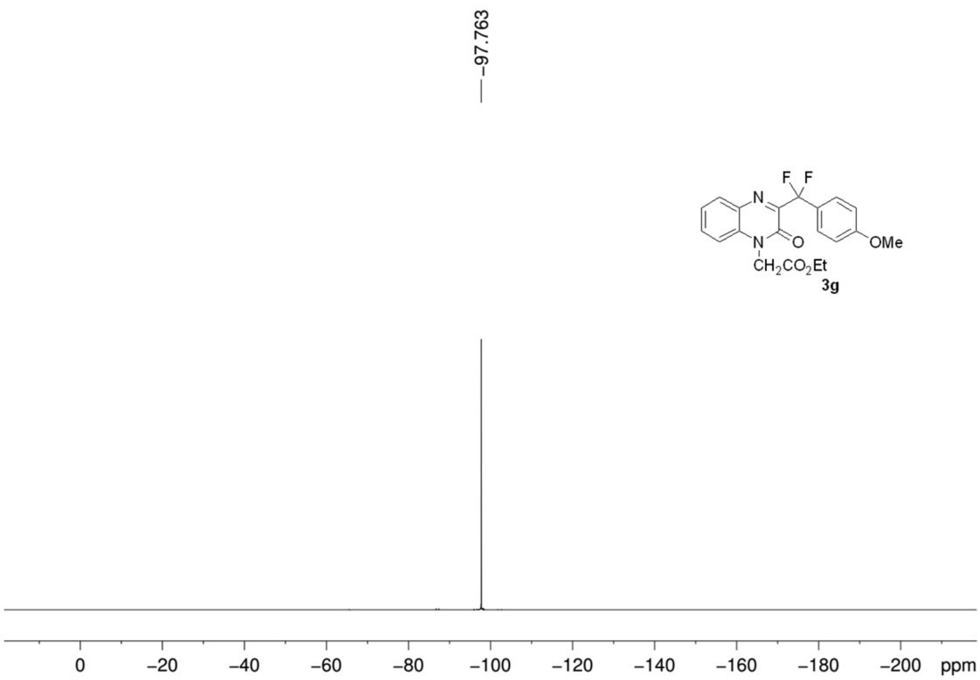

-97.763
F F
N
O
N
OMe
CH_2CO_2Et
3g
0 −20 −40 −60 −80 −100 −120 −140 −160 −180 −200 ppm

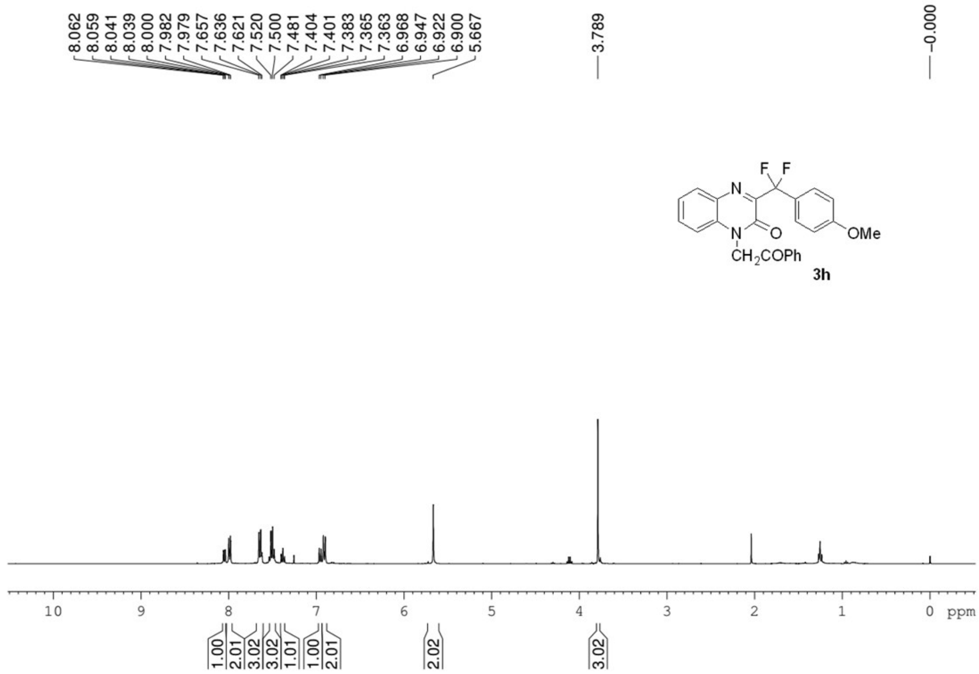

8.062 8.059 8.041 8.039 8.000 7.982 7.979 7.657 7.636 7.621 7.520 7.500 7.481 7.404 7.401 7.383 7.365 7.363 6.968 6.947 6.922 6.900 5.667
3.789
-0.000
F F
N
O
N
OMe
CH_2COPh
3h
10 9 8 7 6 5 4 3 2 1 0 ppm
1.00 2.01 3.02 3.02 1.01 1.00 2.01 2.02 3.02

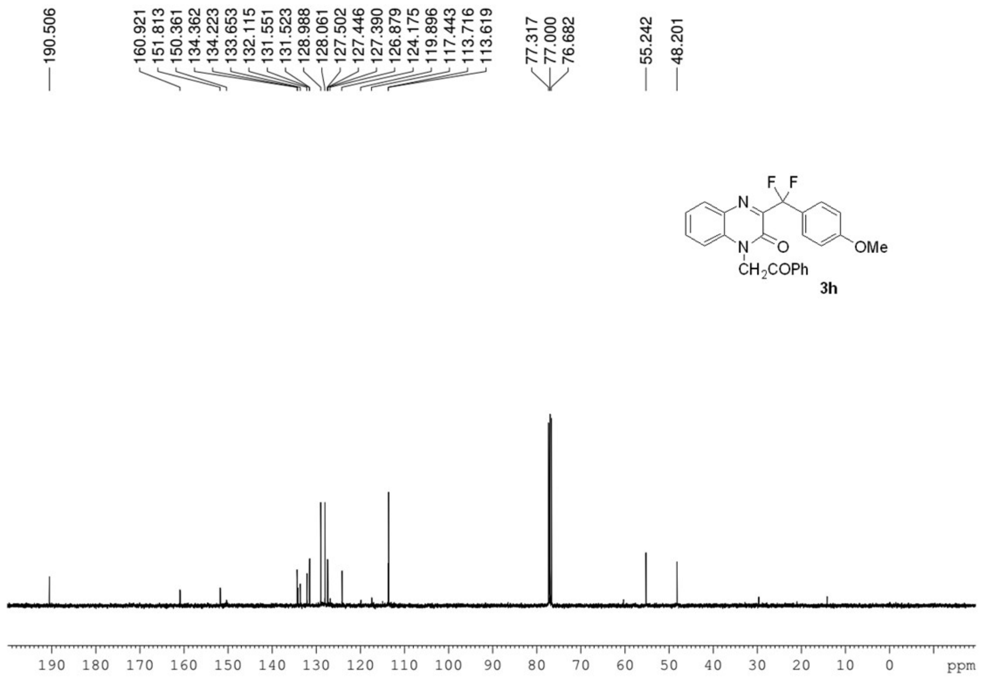
190.506
160.921
151.813
150.361
134.362
134.223
133.653
132.115
131.551
131.523
128.988
128.061
127.502
127.446
127.390
126.879
124.175
119.896
117.443
113.716
113.619
77.317
77.000
76.682
55.242
48.201
OMe
CH2COPh
3h
ppm

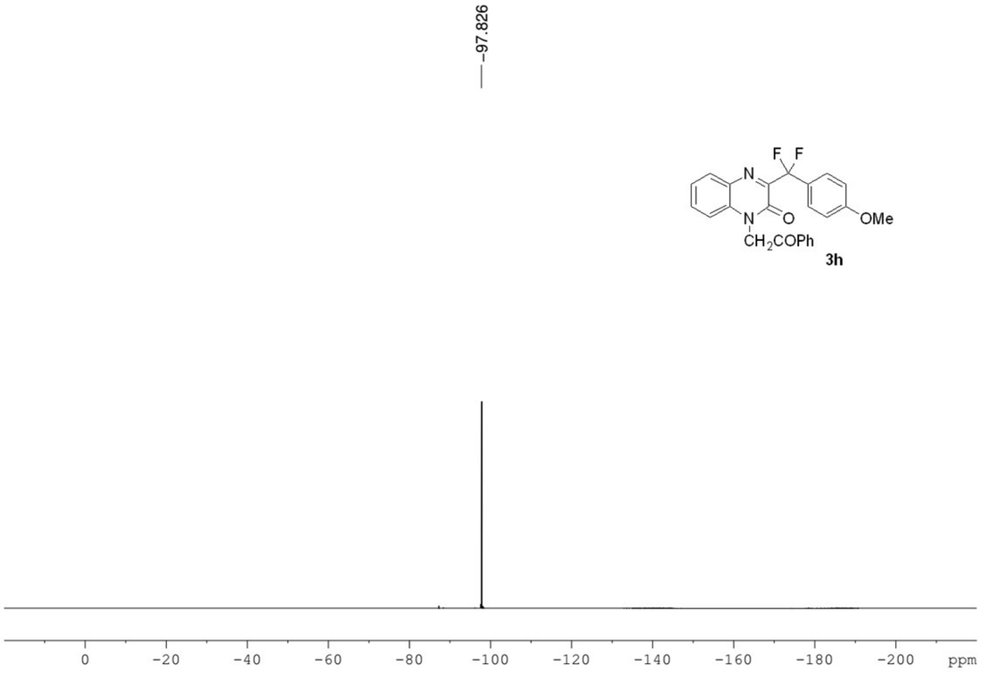
-97.826
OMe
CH2COPh
3h
ppm

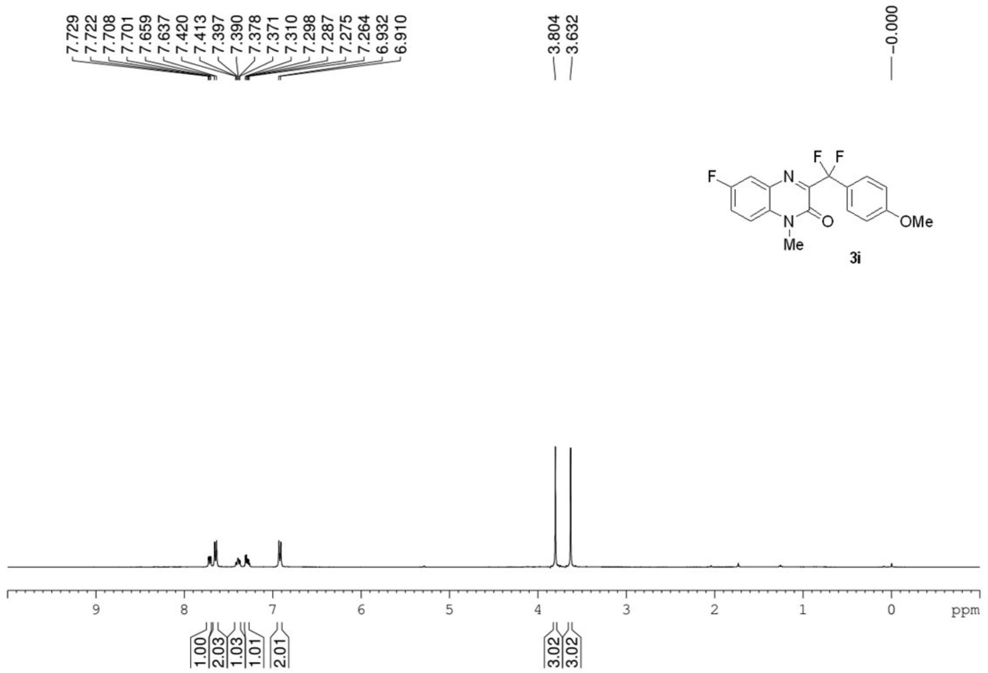
7.729
7.722
7.708
7.701
7.659
7.637
7.420
7.413
7.397
7.390
7.378
7.371
7.310
7.298
7.287
7.275
7.264
6.932
6.910
3.804
3.632
0.000
F
N
F F
N
O
OMe
Me
3i
9 8 7 6 5 4 3 2 1 0 ppm
1.00
2.03
1.03
1.01
2.01
3.02
3.02

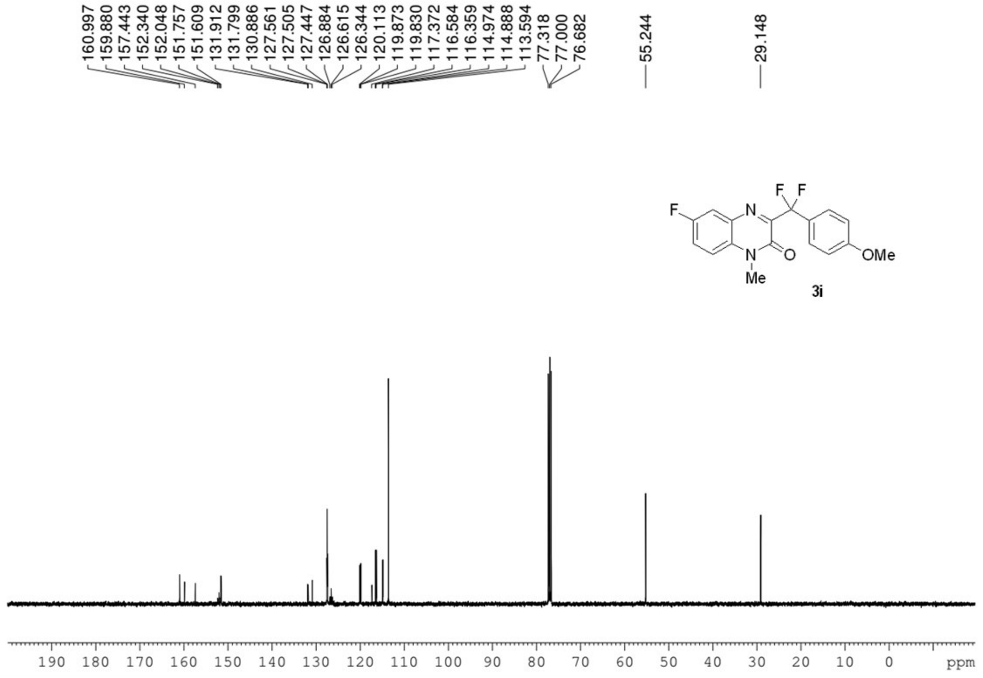
160.997
159.880
157.443
152.340
152.048
151.757
151.609
131.912
131.799
130.886
127.561
127.505
127.447
126.884
126.615
126.344
120.113
119.873
119.830
117.372
116.584
116.359
114.974
114.888
113.594
77.318
77.000
76.682
55.244
29.148
F
N
F F
N
O
OMe
Me
3i
190 180 170 160 150 140 130 120 110 100 90 80 70 60 50 40 30 20 10 0 ppm

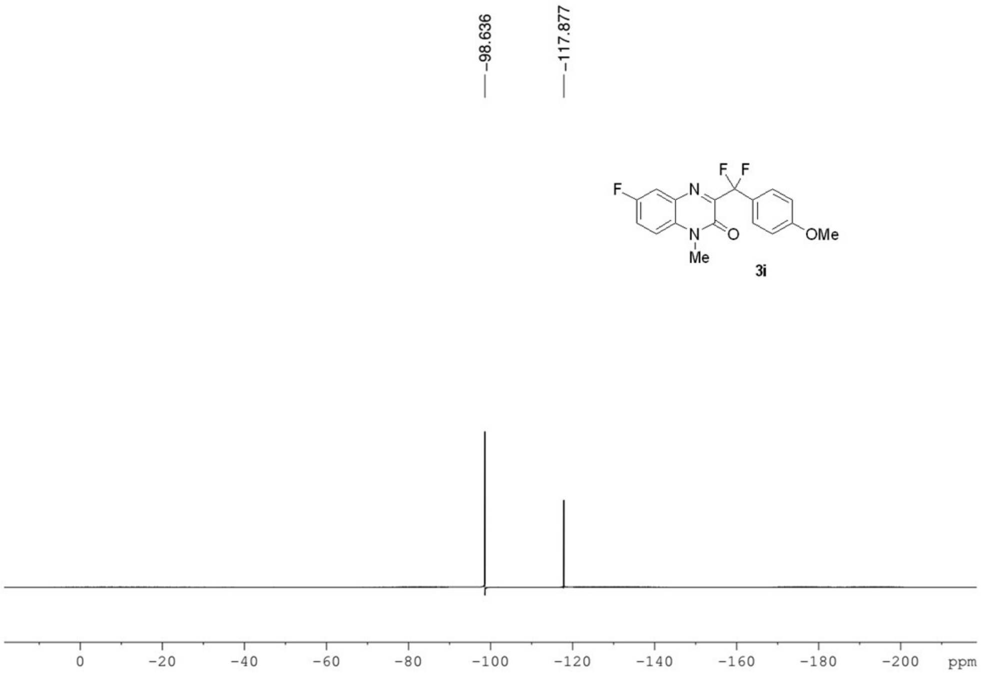

-98.636
-117.877
F
N
F F
O
N
Me
OMe
3i
0 −20 −40 −60 −80 −100 −120 −140 −160 −180 −200 ppm

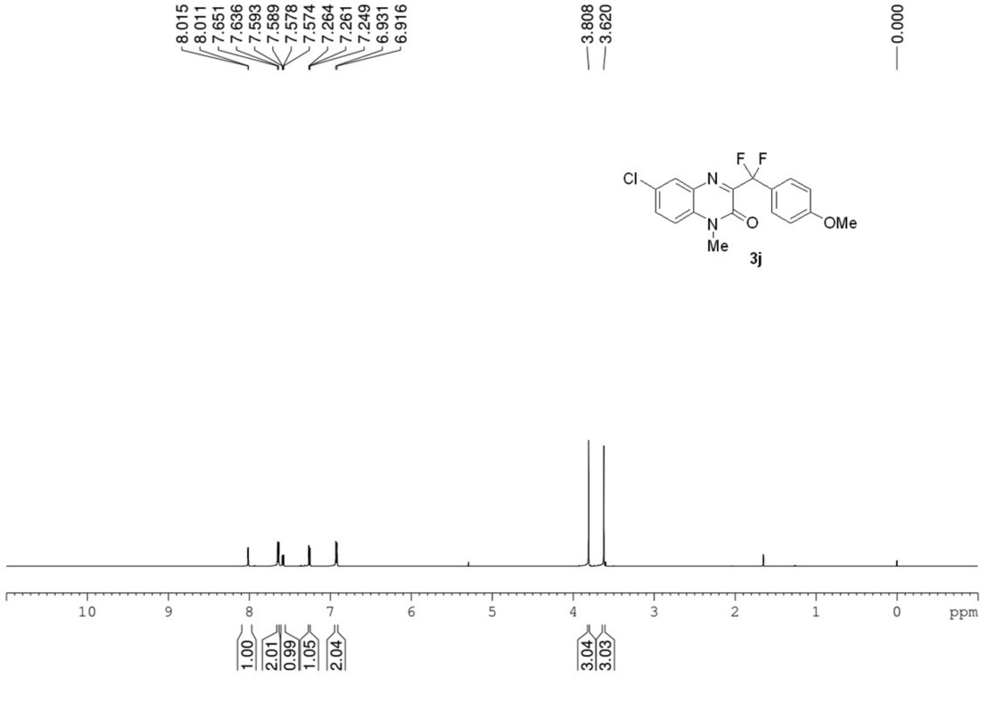

8.015
8.011
7.651
7.636
7.593
7.589
7.578
7.574
7.264
7.261
7.249
6.931
6.916
3.808
3.620
0.000
Cl
N
F F
O
N
Me
OMe
3j
10 9 8 7 6 5 4 3 2 1 0 ppm
1.00
2.01
0.99
1.05
2.04
3.04
3.03

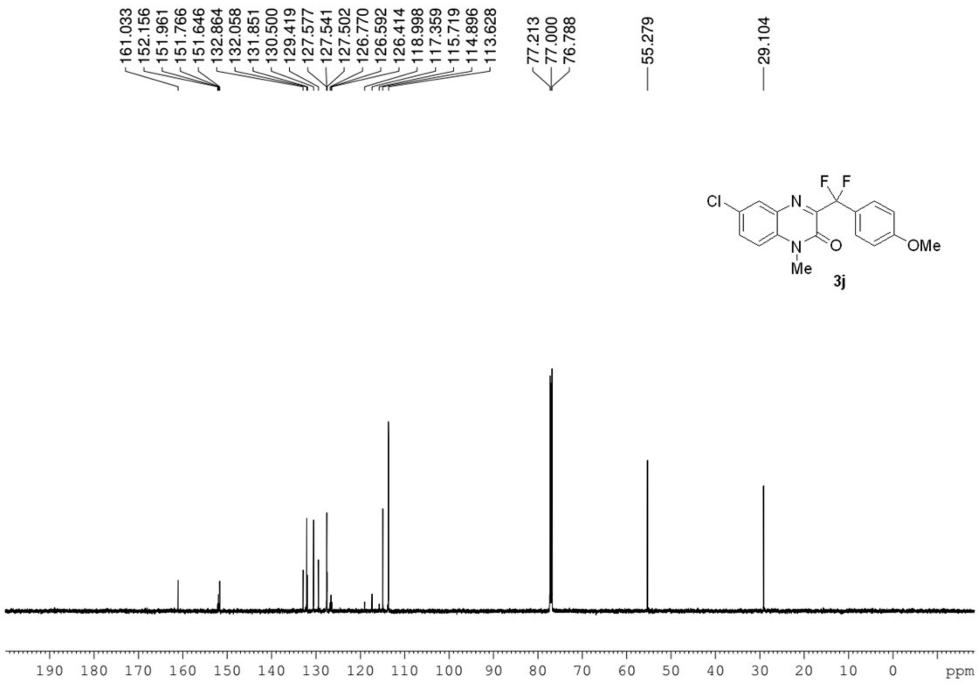
161.033
152.156
151.961
151.766
151.646
132.864
132.058
131.851
130.500
129.419
127.577
127.541
127.502
126.770
126.592
126.414
118.998
117.359
115.719
114.896
113.628
77.213
77.000
76.788
55.279
29.104
Cl
N
F
F
N
O
OMe
Me
3j
190 180 170 160 150 140 130 120 110 100 90 80 70 60 50 40 30 20 10 0 ppm

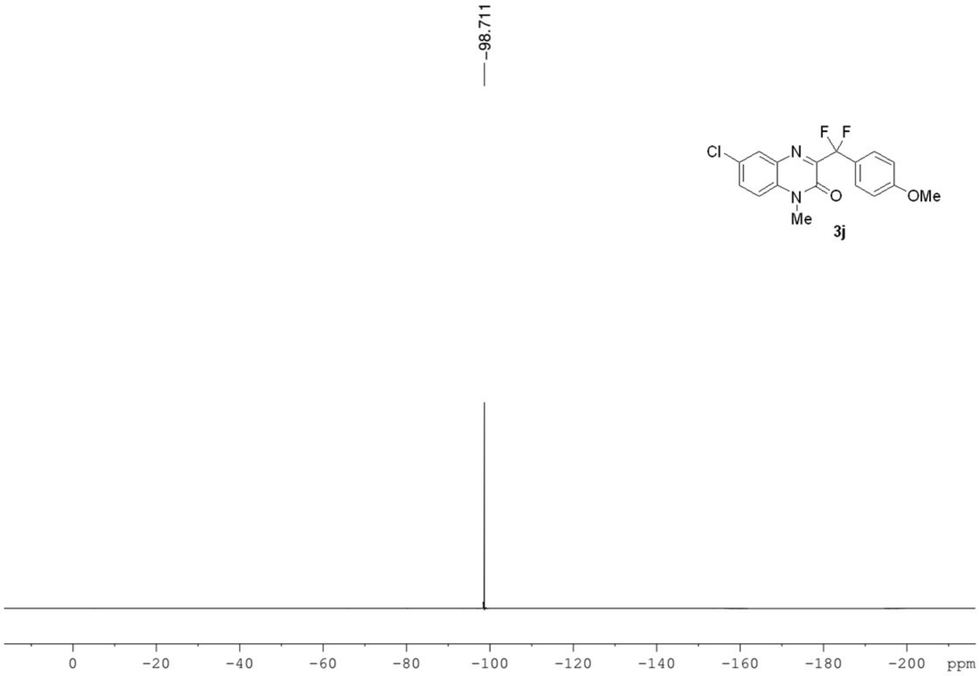
-98.711
Cl
N
F
F
N
O
OMe
Me
3j
0 -20 -40 -60 -80 -100 -120 -140 -160 -180 -200 ppm

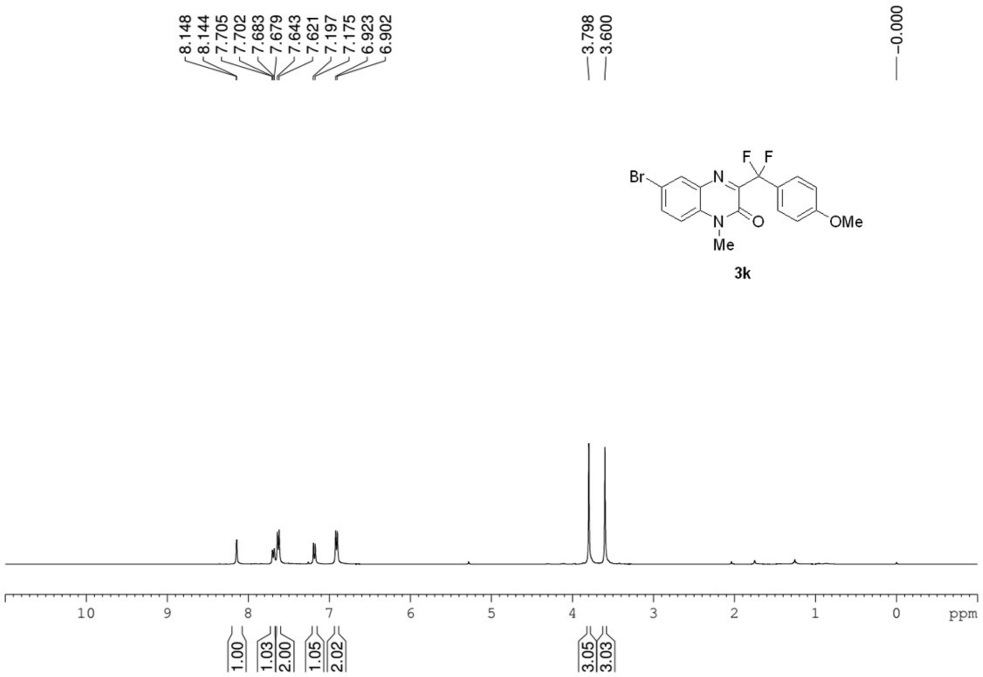
8.148
8.144
7.705
7.702
7.683
7.679
7.643
7.621
7.197
7.175
6.923
6.902
3.798
3.600
0.000
Br
N
F F
N
O
OMe
Me
3k
10 9 8 7 6 5 4 3 2 1 0 ppm
1.00
1.03
2.00
1.05
2.02
3.05
3.03

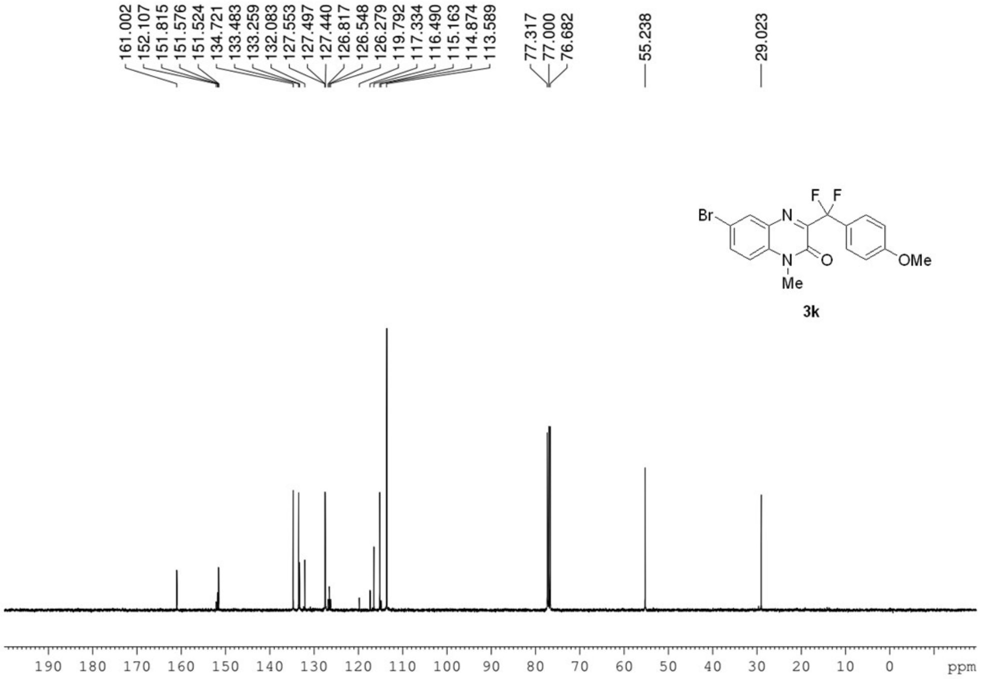
161.002
152.107
151.815
151.576
151.524
134.721
133.483
133.259
132.083
127.553
127.497
127.440
126.817
126.548
126.279
119.792
117.334
116.490
115.163
114.874
113.589
77.317
77.000
76.682
55.238
29.023
Br
N
F F
N
O
OMe
Me
3k
190 180 170 160 150 140 130 120 110 100 90 80 70 60 50 40 30 20 10 0 ppm

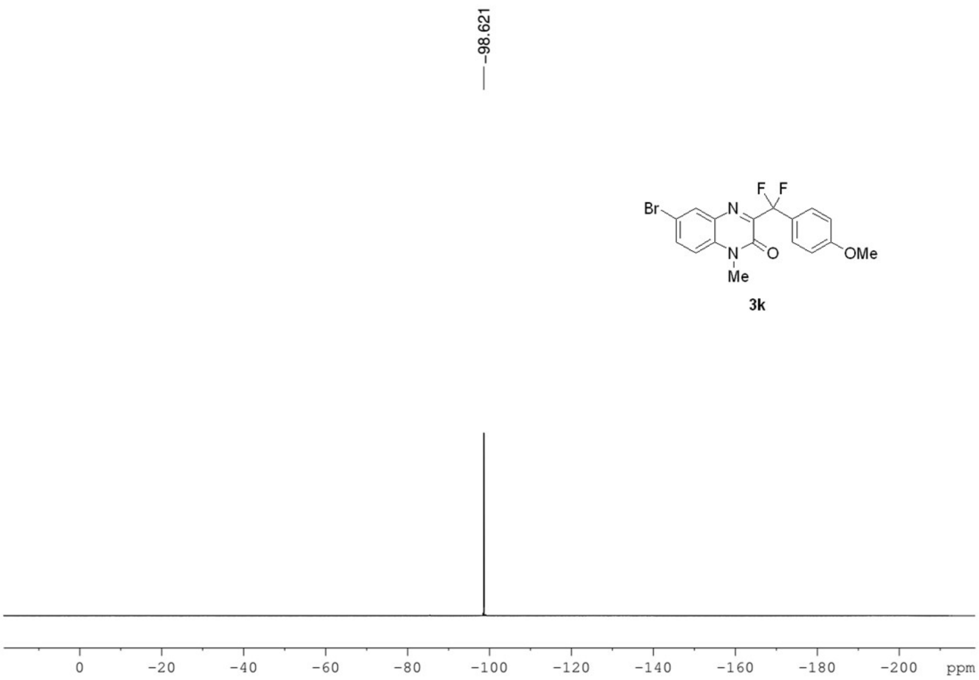

-98.621
Br
F F
N
OMe
N O
Me
3k
0 -20 -40 -60 -80 -100 -120 -140 -160 -180 -200 ppm

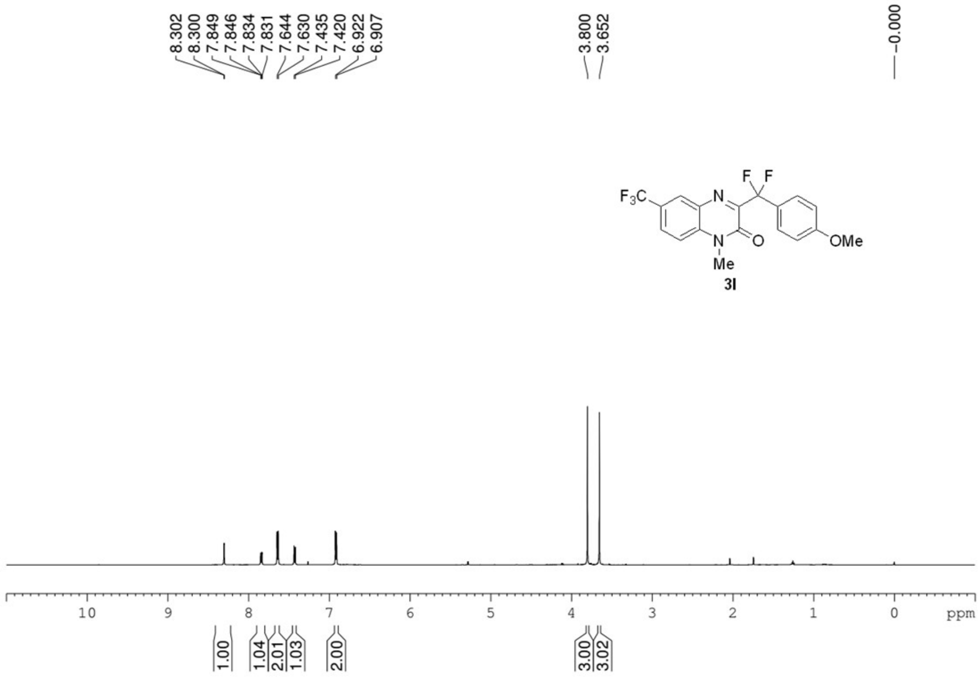

8.302 8.300 7.849 7.846 7.834 7.831 7.644 7.630 7.435 7.420 6.922 6.907
3.800 3.652
-0.000
F_3C
F F
N
OMe
N O
Me
3l
10 9 8 7 6 5 4 3 2 1 0 ppm
1.00 1.04 2.01 1.03 2.00 3.00 3.02

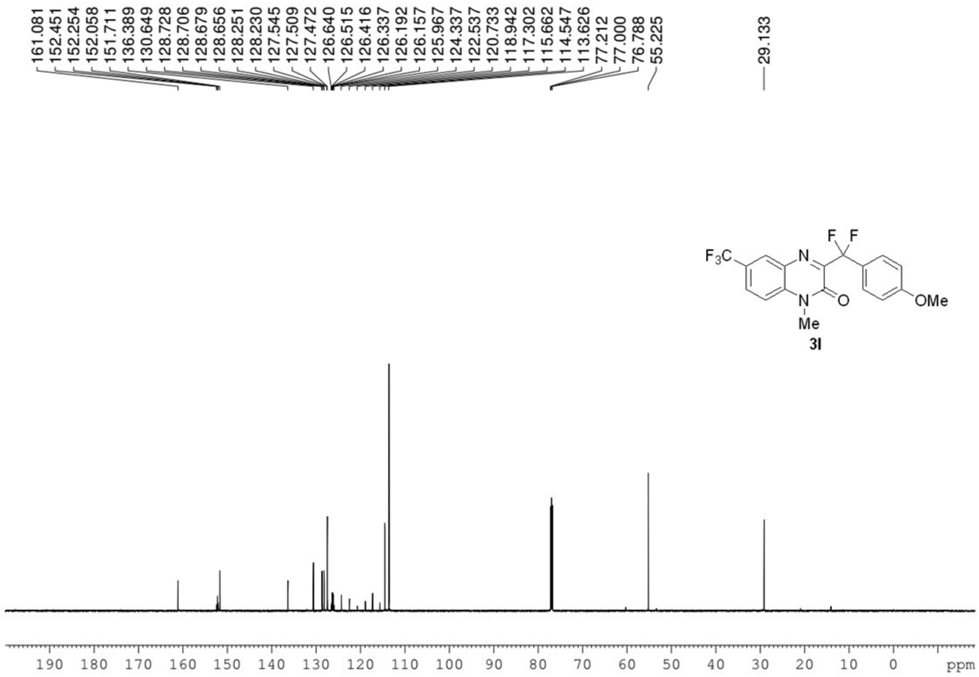
161.081
152.451
152.254
152.058
151.711
136.389
130.649
128.728
128.706
128.679
128.656
128.251
128.230
127.545
127.509
127.472
126.640
126.515
126.416
126.337
126.192
126.157
125.967
124.337
122.537
120.733
118.942
117.302
115.662
114.547
113.626
77.212
77.000
76.788
55.225
29.133
F F
F3C
N
N
O
OMe
Me
3l
190 180 170 160 150 140 130 120 110 100 90 80 70 60 50 40 30 20 10 0 ppm

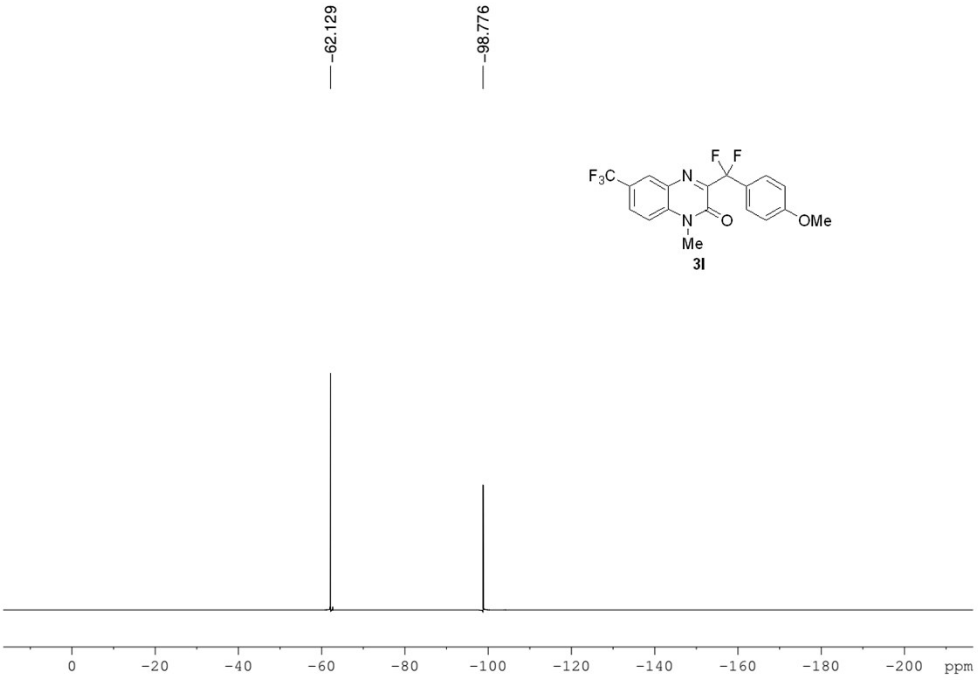
-62.129
-98.776
F F
F3C
N
N
O
OMe
Me
3l
0 -20 -40 -60 -80 -100 -120 -140 -160 -180 -200 ppm

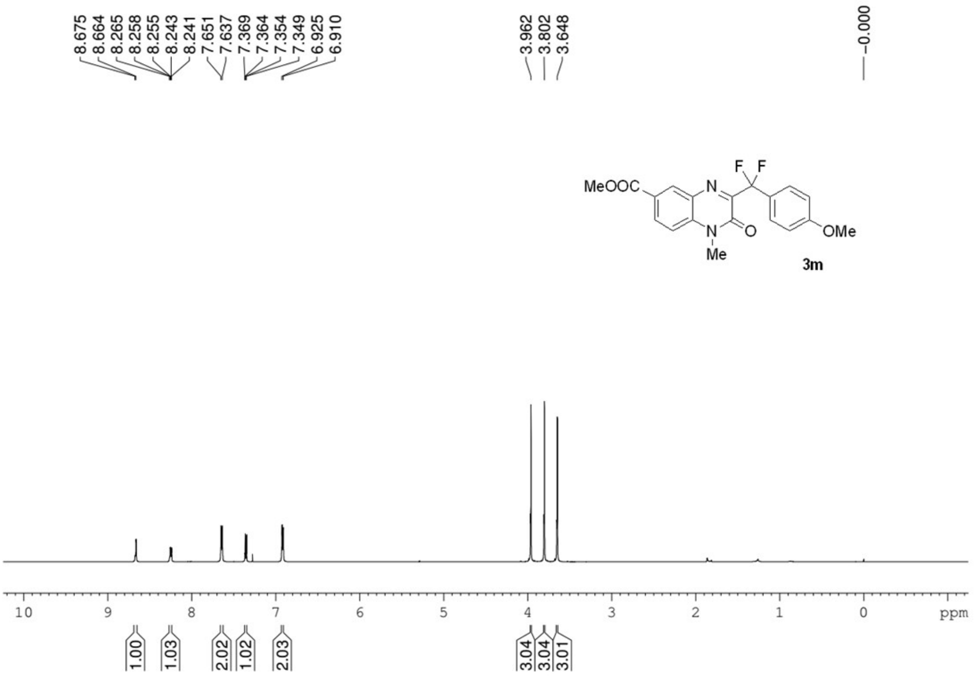
8.675
8.664
8.265
8.258
8.255
8.243
8.241
7.651
7.637
7.369
7.364
7.354
7.349
6.925
6.910
3.962
3.802
3.648
0.000
MeOOC
N
F
F
N
Me
O
OMe
3m
10
9
8
7
6
5
4
3
2
1
0
ppm
1.00
1.03
2.02
1.02
2.03
3.04
3.04
3.01

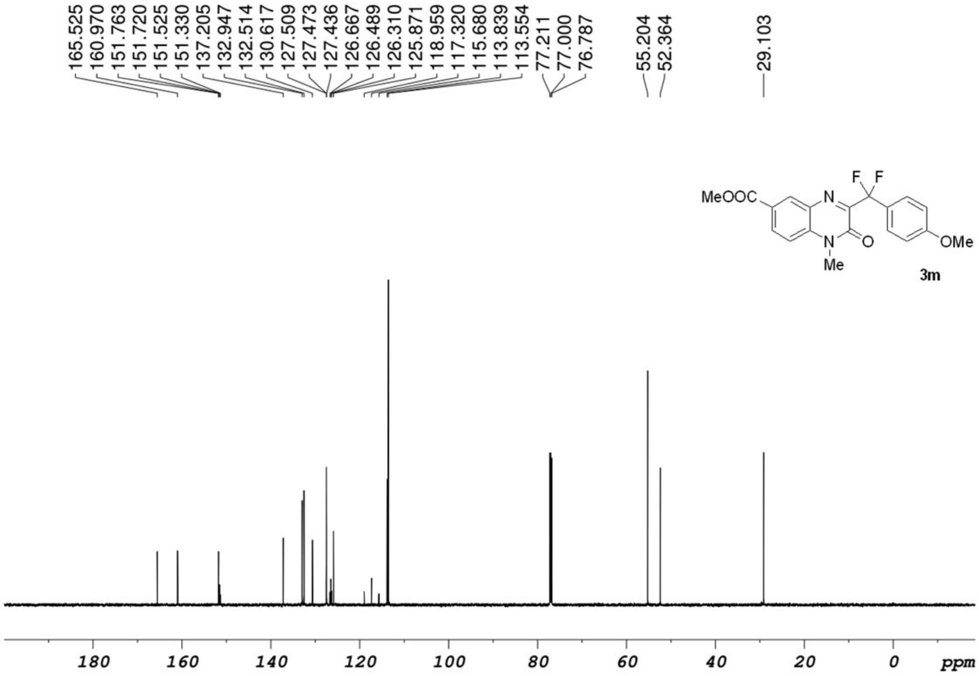
165.525
160.970
151.763
151.720
151.525
151.330
137.205
132.947
132.514
130.617
127.509
127.473
127.436
126.667
126.489
126.310
125.871
118.959
117.320
115.680
113.839
113.554
77.211
77.000
76.787
55.204
52.364
29.103
MeOOC
N
F
F
N
Me
O
OMe
3m
180
160
140
120
100
80
60
40
20
0
ppm

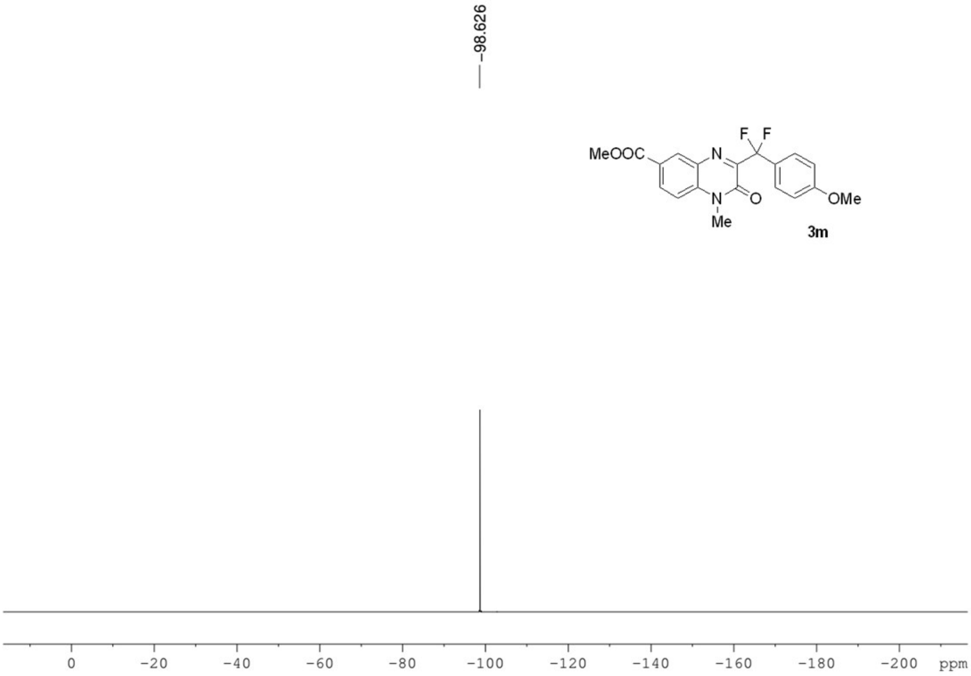
-98.626
MeOOC
N
F F
O
N
Me
OMe
3m
0 -20 -40 -60 -80 -100 -120 -140 -160 -180 -200 ppm

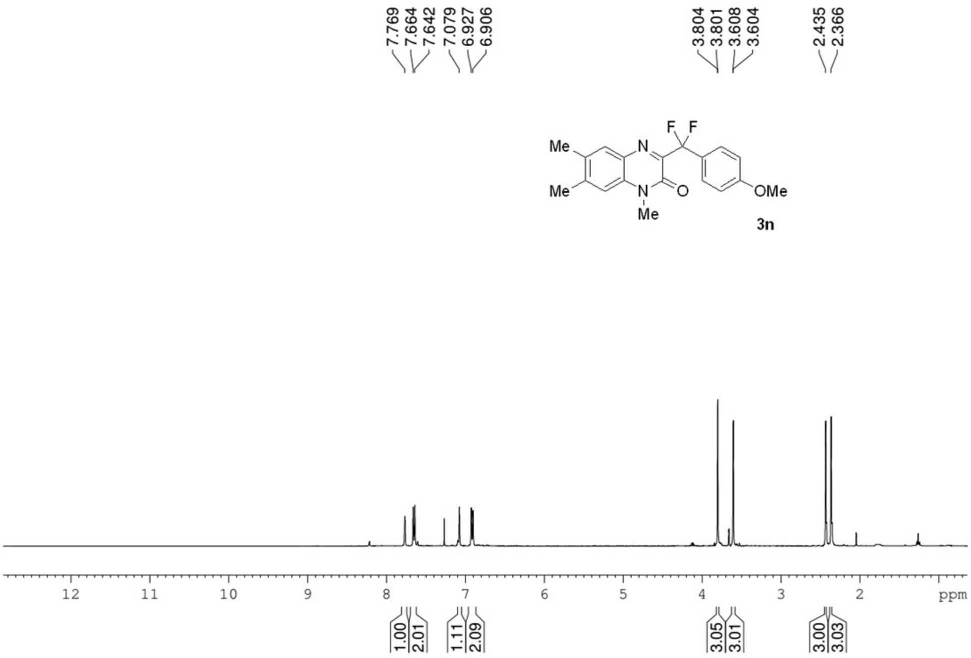
7.769
7.664
7.642
7.079
6.927
6.906
3.804
3.801
3.608
3.604
2.435
2.366
Me
Me
N
F F
O
N
Me
OMe
3n
12 11 10 9 8 7 6 5 4 3 2 ppm
1.00
2.01
1.11
2.09
3.05
3.01
3.00
3.03

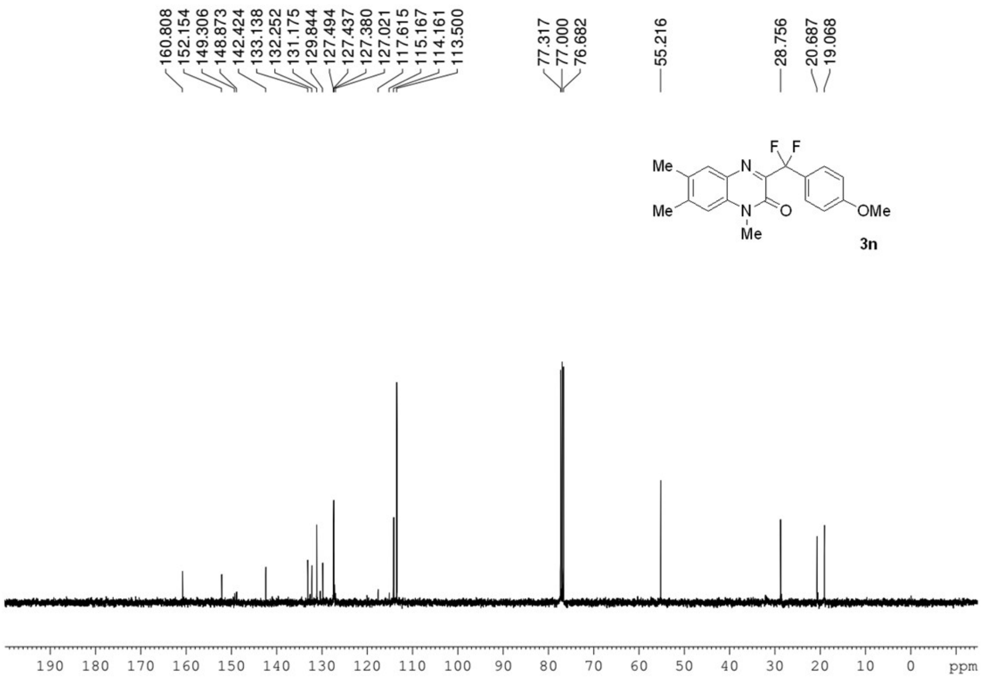
160.808
152.154
149.306
148.873
142.424
133.138
132.252
131.175
129.844
127.494
127.437
127.380
127.021
117.615
115.167
114.161
113.500
77.317
77.000
76.682
55.216
28.756
20.687
19.068
Me
Me
N
N
Me
F F
O
OMe
3n
190 180 170 160 150 140 130 120 110 100 90 80 70 60 50 40 30 20 10 0 ppm

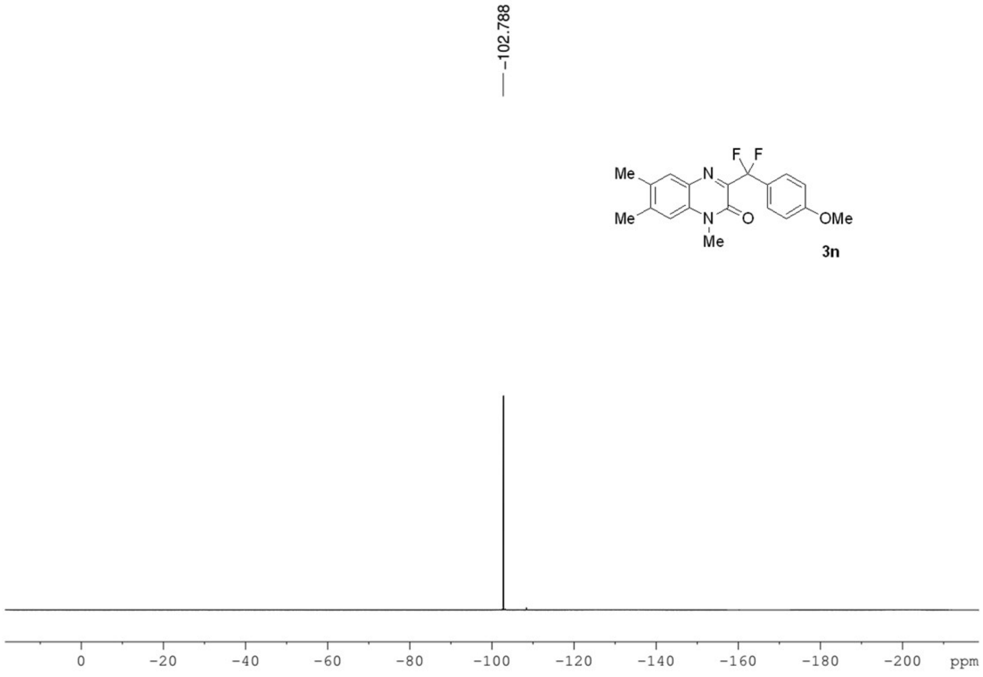
-102.788
Me
Me
N
N
Me
F F
O
OMe
3n
0 -20 -40 -60 -80 -100 -120 -140 -160 -180 -200 ppm

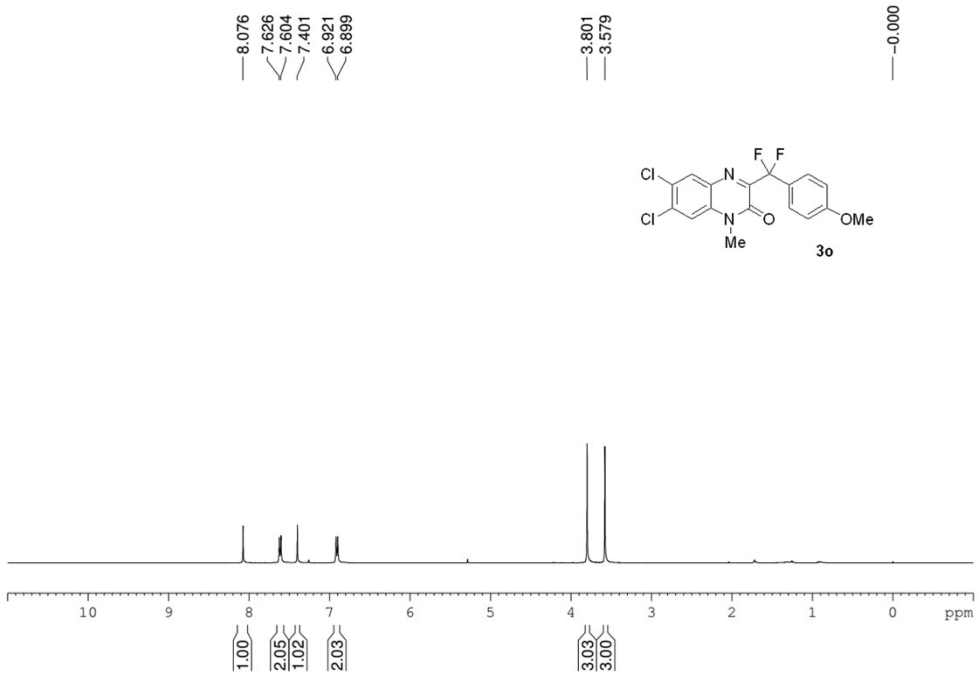
8.076
7.626
7.604
7.401
6.921
6.899
3.801
3.579
-0.000
Cl
Cl
N
N
F
F
O
Me
OMe
3o
10 9 8 7 6 5 4 3 2 1 0 ppm
1.00
2.05
1.02
2.03
3.03
3.00

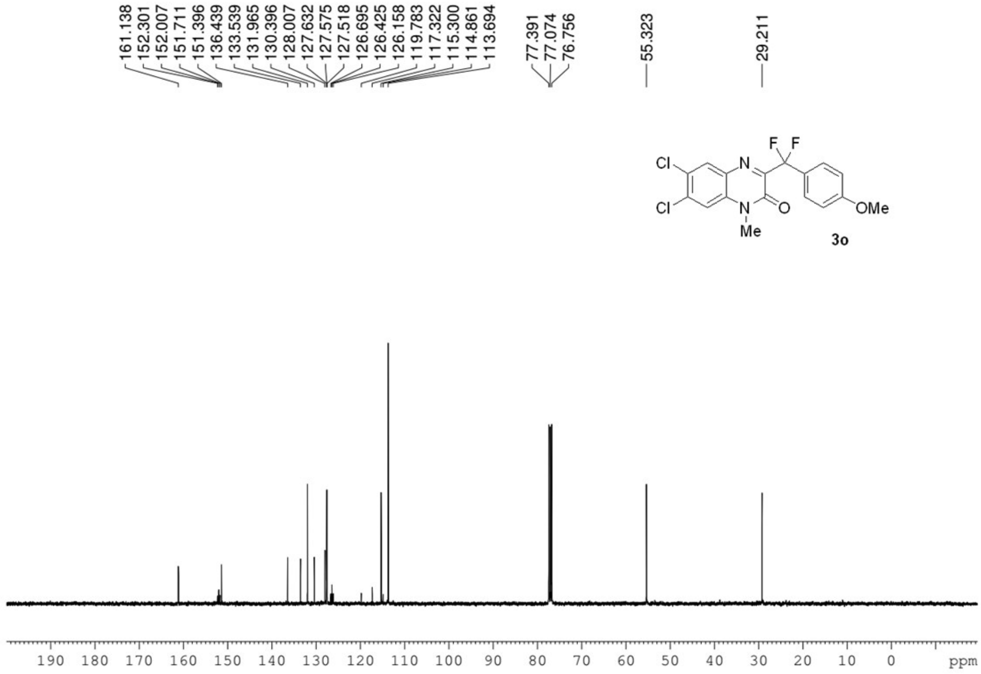
161.138
152.301
152.007
151.711
151.396
136.439
133.539
131.965
130.396
128.007
127.632
127.575
127.518
126.695
126.425
126.158
119.783
117.322
115.300
114.861
113.694
77.391
77.074
76.756
55.323
29.211
Cl
Cl
N
N
F
F
O
Me
OMe
3o
190 180 170 160 150 140 130 120 110 100 90 80 70 60 50 40 30 20 10 0 ppm

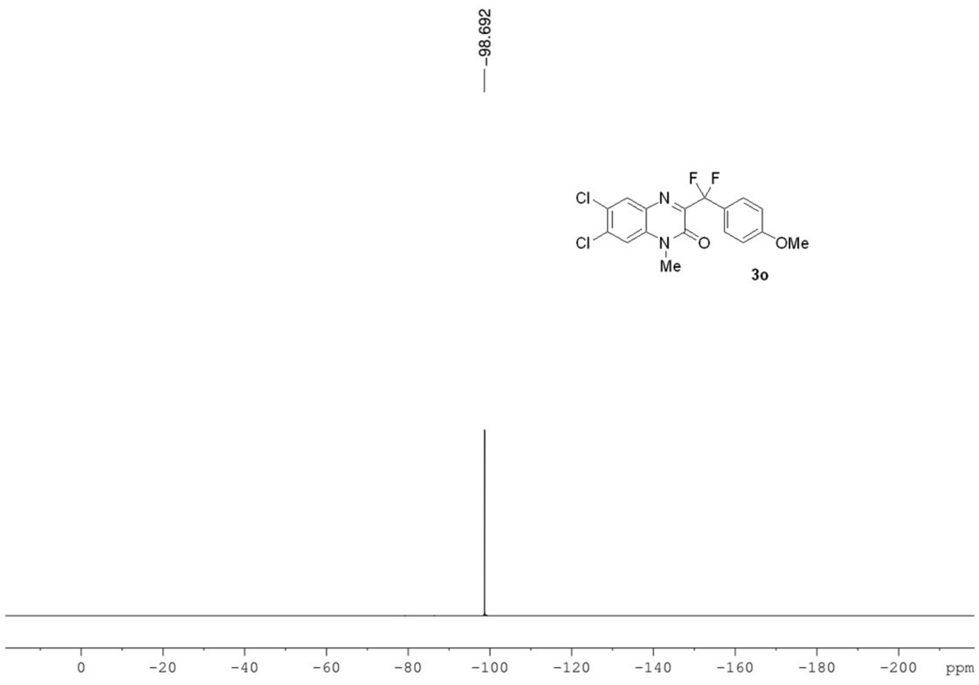

-98.692
Cl
Cl
N
F F
O
OMe
N
Me
3o
0
-20
-40
-60
-80
-100
-120
-140
-160
-180
-200
ppm

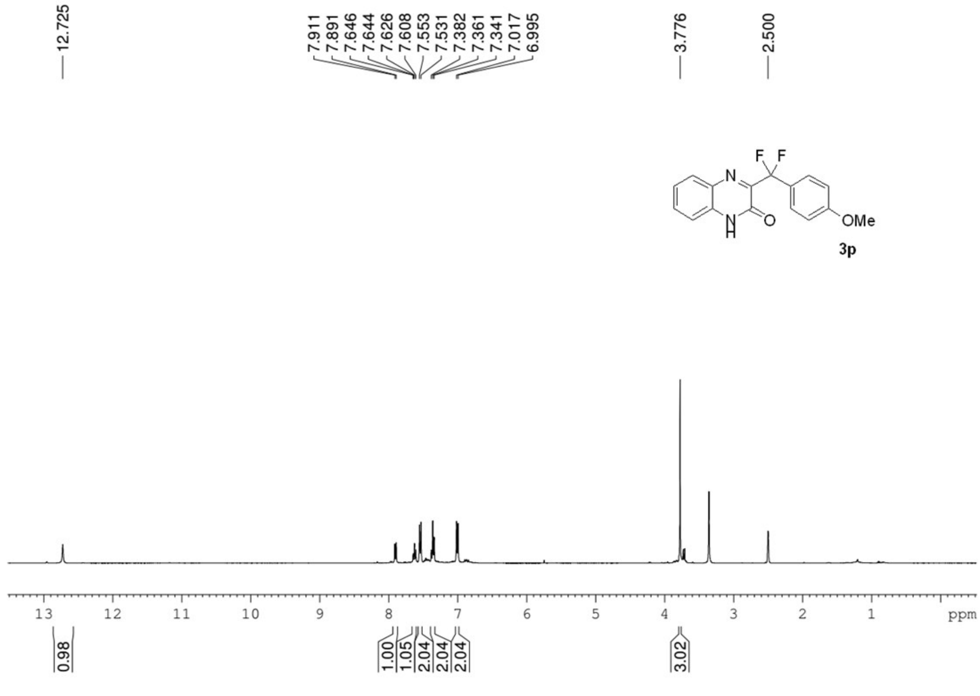

12.725
7.911
7.891
7.646
7.644
7.626
7.608
7.553
7.531
7.382
7.361
7.341
7.017
6.995
3.776
2.500
N
F F
OMe
N
H
O
3p
13
12
11
10
9
8
7
6
5
4
3
2
1
ppm
0.98
1.00
1.05
2.04
2.04
2.04
3.02

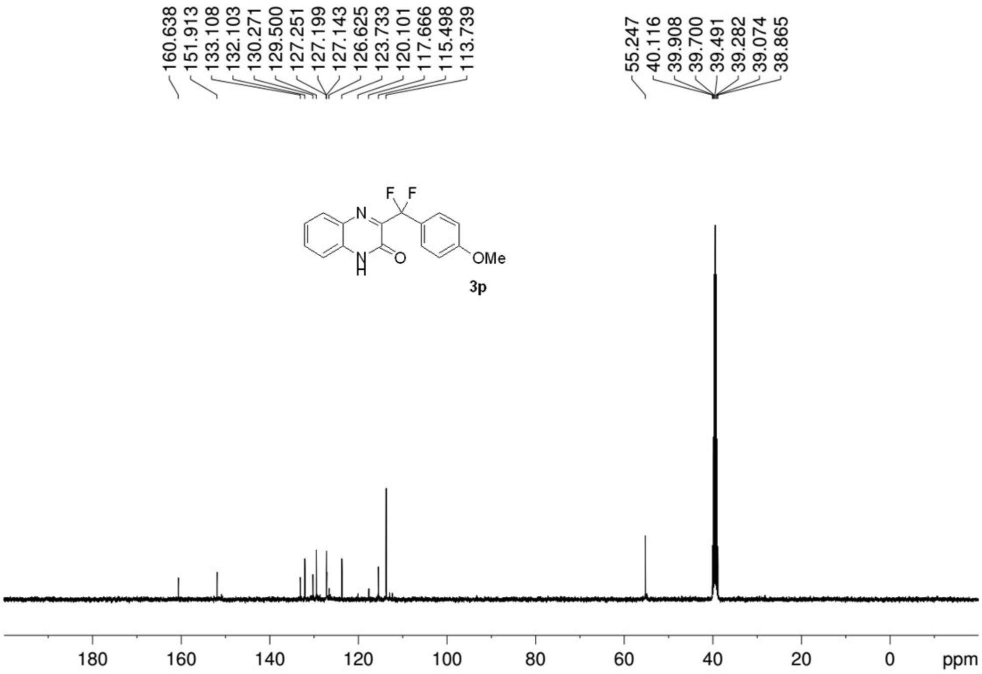
160.638
151.913
133.108
132.103
130.271
129.500
127.251
127.199
127.143
126.625
123.733
120.101
117.666
115.498
113.739
55.247
40.116
39.908
39.700
39.491
39.282
39.074
38.865
N
F F
N
H
O
OMe
3p
180
160
140
120
100
80
60
40
20
0
ppm

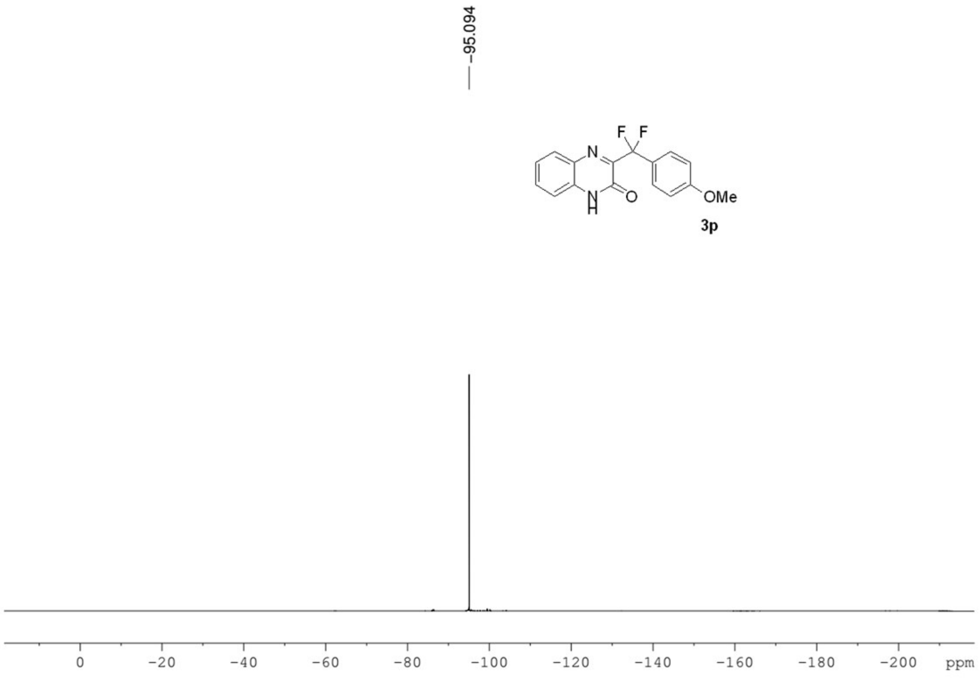
-95.094
N
F F
N
H
O
OMe
3p
0
-20
-40
-60
-80
-100
-120
-140
-160
-180
-200
ppm

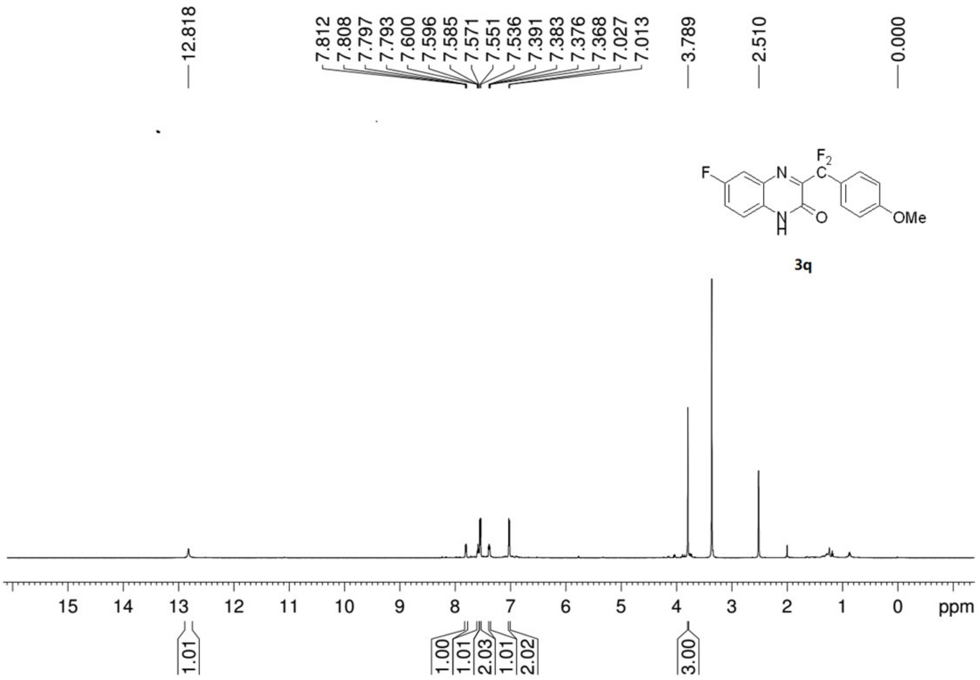
12.818
7.812 7.808 7.797 7.793 7.600 7.596 7.585 7.571 7.551 7.536 7.391 7.383 7.376 7.368 7.027 7.013
3.789
2.510
0.000
F
N
F2
C
H
O
OMe
3q
15 14 13 12 11 10 9 8 7 6 5 4 3 2 1 0 ppm
1.01
1.00 1.01 2.03 1.01 2.02
3.00

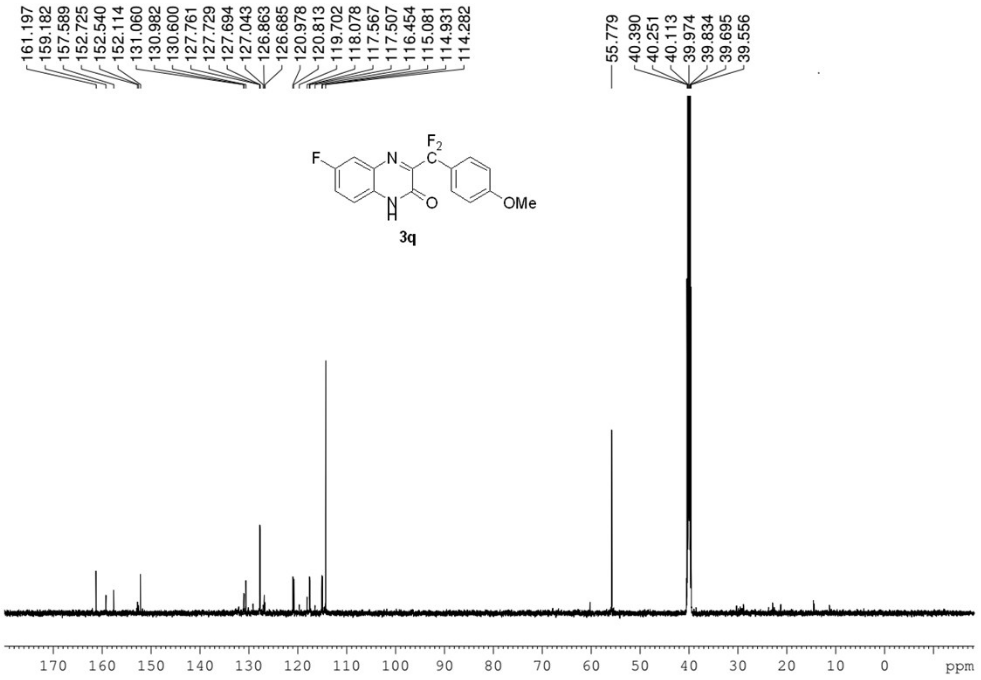
161.197 159.182 157.589 152.725 152.540 152.114 131.060 130.982 130.600 127.761 127.729 127.694 127.043 126.863 126.685 120.978 120.813 119.702 118.078 117.567 117.507 116.454 115.081 114.931 114.282
55.779
40.390 40.251 40.113 39.974 39.834 39.695 39.556
F
N
F2
C
H
O
OMe
3q
170 160 150 140 130 120 110 100 90 80 70 60 50 40 30 20 10 0 ppm

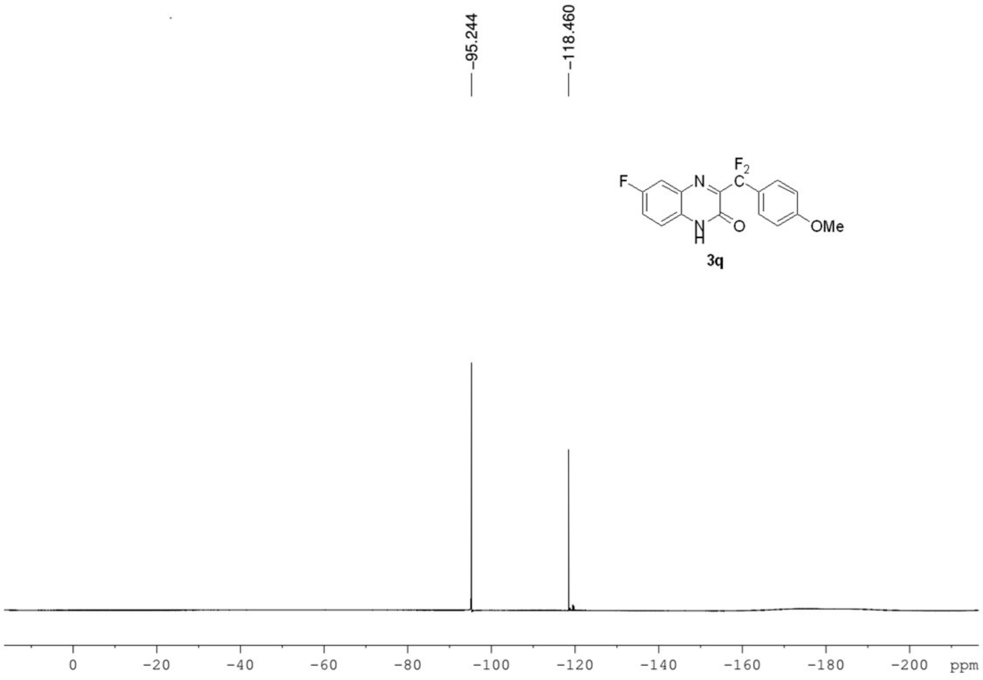

-95.244
-118.460
F
N
F2
C
N
H
O
OMe
3q
0 -20 -40 -60 -80 -100 -120 -140 -160 -180 -200 ppm

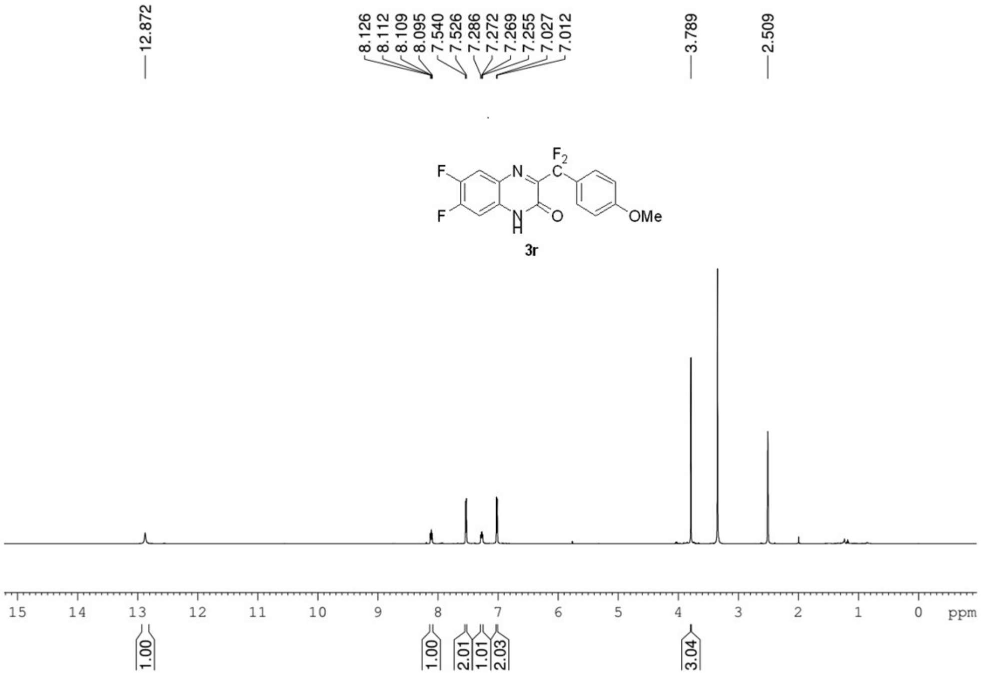

12.872
8.126
8.112
8.109
8.095
7.540
7.526
7.286
7.272
7.269
7.255
7.027
7.012
3.789
2.509
F
F
N
F2
C
N
H
O
OMe
3r
15 14 13 12 11 10 9 8 7 6 5 4 3 2 1 0 ppm
1.00
1.00
2.01
1.01
2.03
3.04

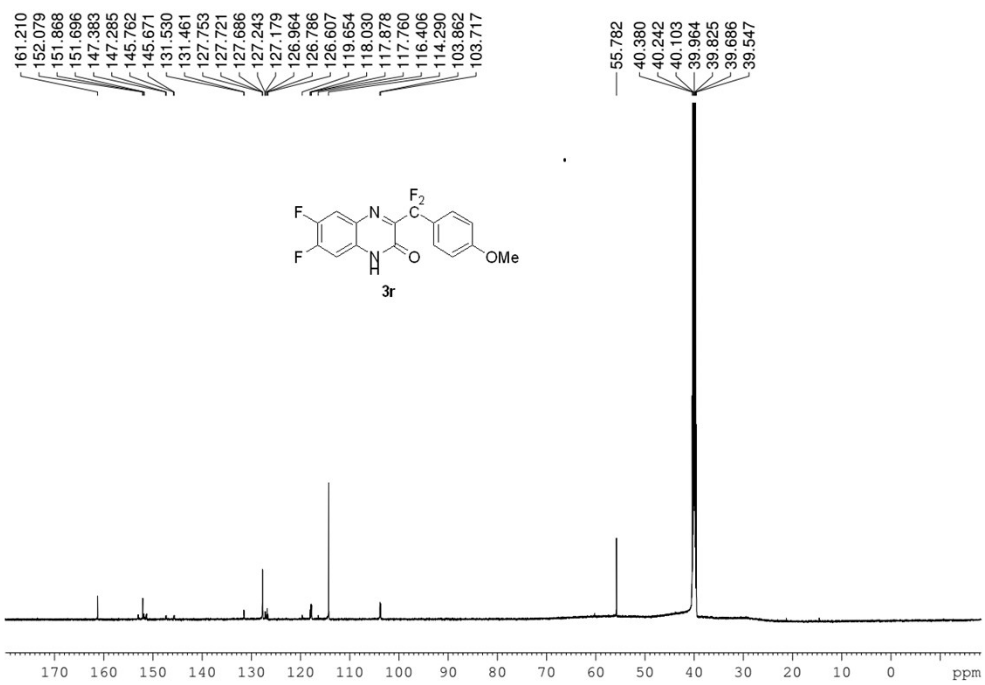
161.210
152.079
151.868
151.696
147.383
147.285
145.762
145.671
131.530
131.461
127.753
127.721
127.686
127.243
127.179
126.964
126.786
126.607
119.654
118.030
117.878
117.760
116.406
114.290
103.862
103.717
55.782
40.380
40.242
40.103
39.964
39.825
39.686
39.547
3r
170 160 150 140 130 120 110 100 90 80 70 60 50 40 30 20 10 0 ppm

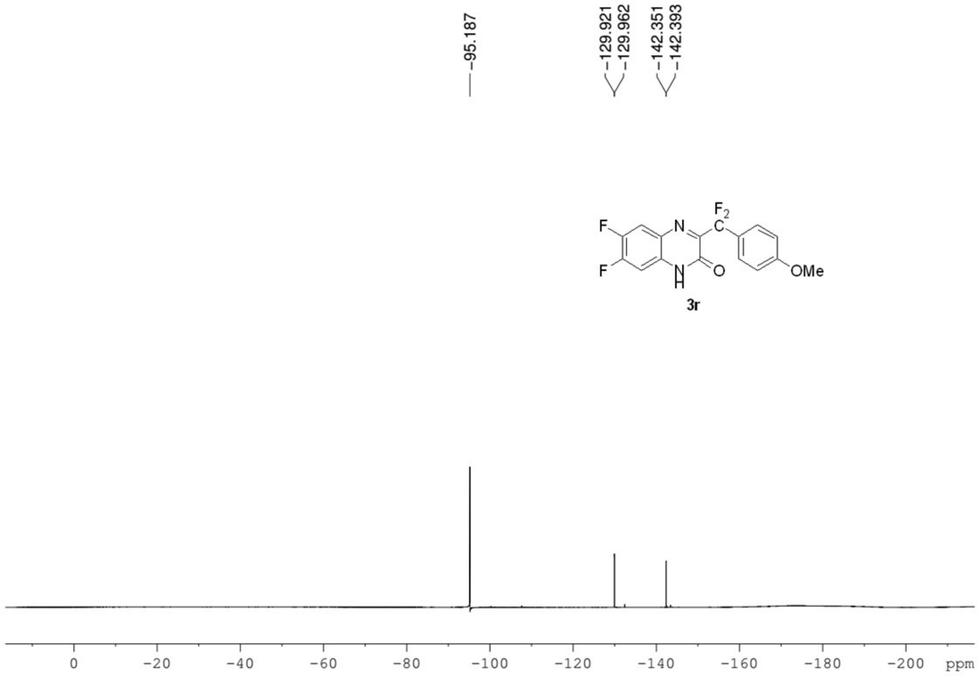
-95.187
-129.921
-129.962
-142.351
-142.393
3r
0 -20 -40 -60 -80 -100 -120 -140 -160 -180 -200 ppm

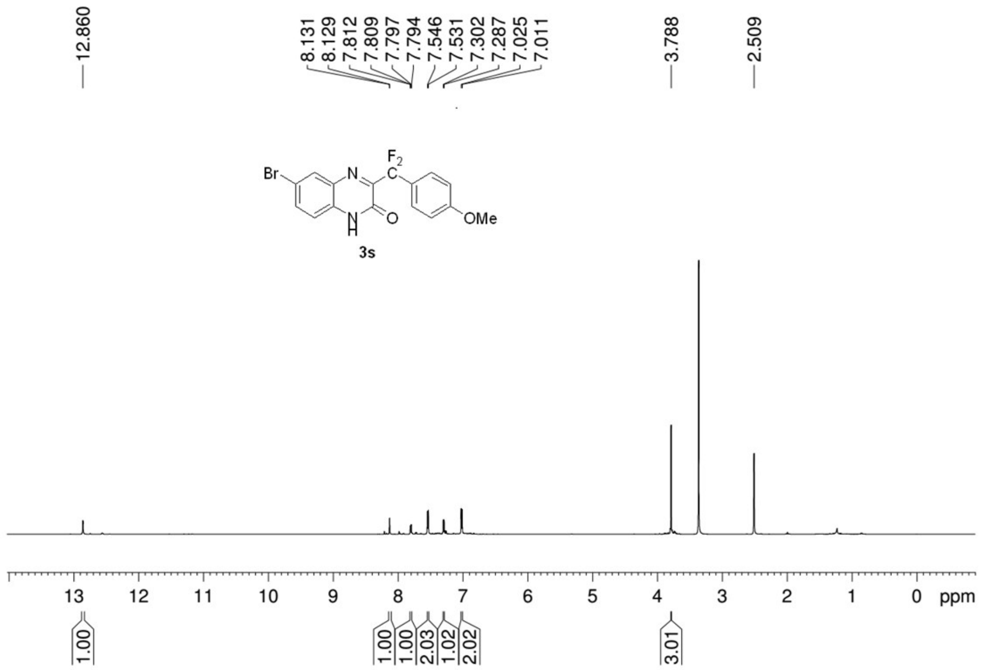
12.860
8.131
8.129
7.812
7.809
7.797
7.794
7.546
7.531
7.302
7.287
7.025
7.011
3.788
2.509
Br
N
F2
C
O
N
H
OMe
3s
13 12 11 10 9 8 7 6 5 4 3 2 1 0 ppm
1.00
1.00
1.00
2.03
1.02
2.02
3.01

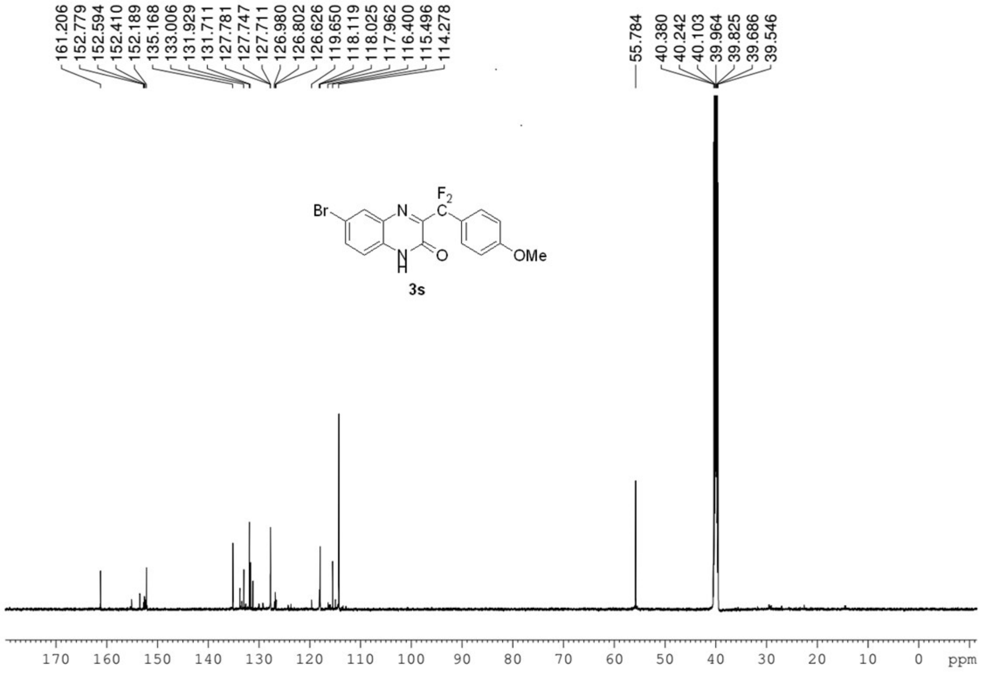
161.206
152.779
152.594
152.410
152.189
135.168
133.006
131.929
131.711
127.781
127.747
127.711
126.980
126.802
126.626
119.650
118.119
118.025
117.962
116.400
115.496
114.278
55.784
40.380
40.242
40.103
39.964
39.825
39.686
39.546
Br
N
F2
C
O
N
H
OMe
3s
170 160 150 140 130 120 110 100 90 80 70 60 50 40 30 20 10 0 ppm

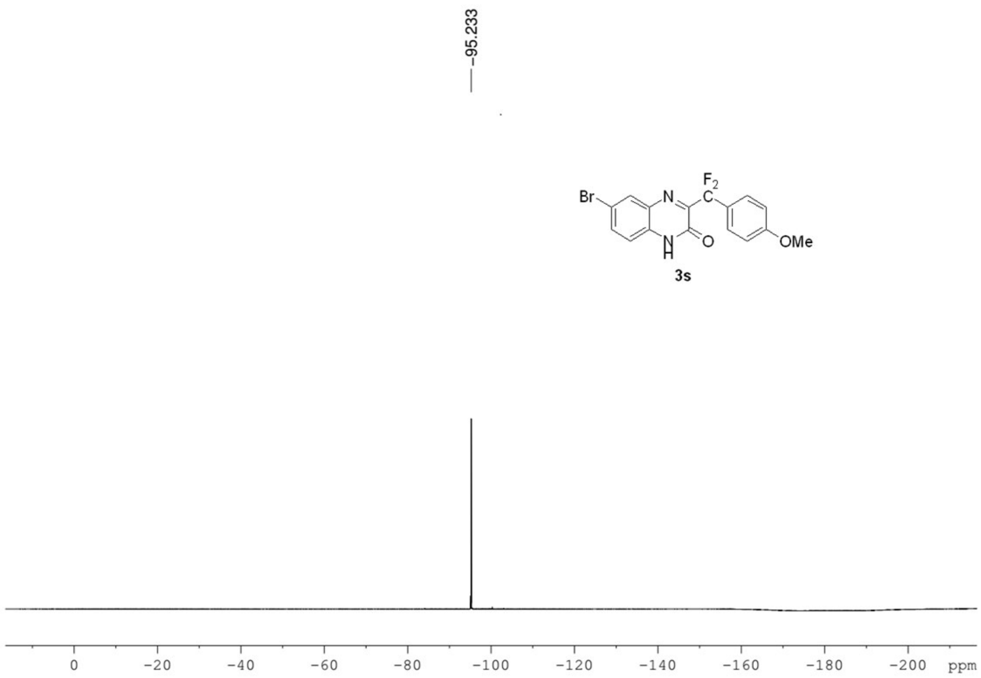
-95.233
Br
N
F2
C
N
H
O
OMe
3s
0
-20
-40
-60
-80
-100
-120
-140
-160
-180
-200
ppm

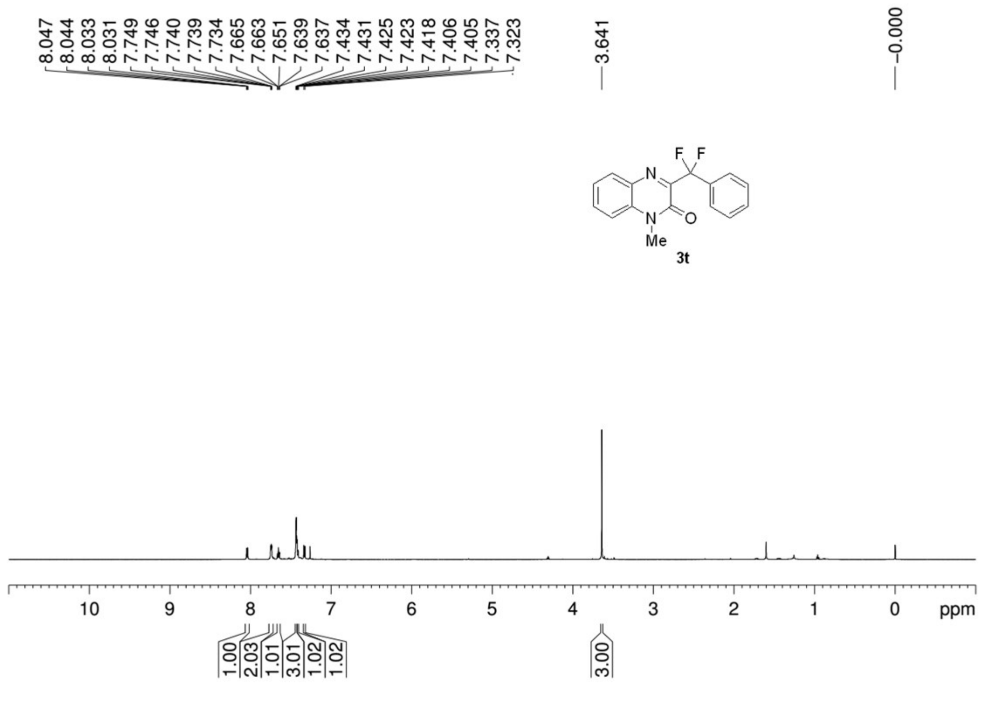
8.047
8.044
8.033
8.031
7.749
7.746
7.740
7.739
7.734
7.665
7.663
7.651
7.639
7.637
7.434
7.431
7.425
7.423
7.418
7.406
7.405
7.337
7.323
3.641
-0.000
F F
N
N
O
Me
3t
10
9
8
7
6
5
4
3
2
1
0
ppm
1.00
2.03
1.01
3.01
1.02
1.02
3.00

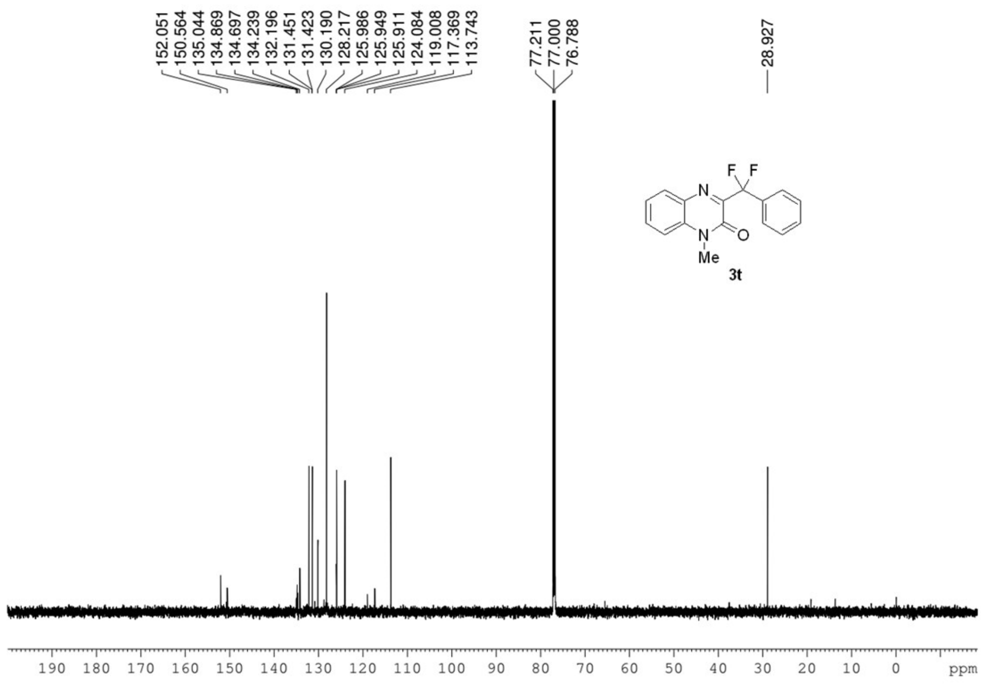
152.051
150.564
135.044
134.869
134.697
134.239
132.196
131.451
131.423
130.190
128.217
125.986
125.949
125.911
124.084
119.008
117.369
113.743
77.211
77.000
76.788
28.927
F F
N
N
Me
O
3t
ppm

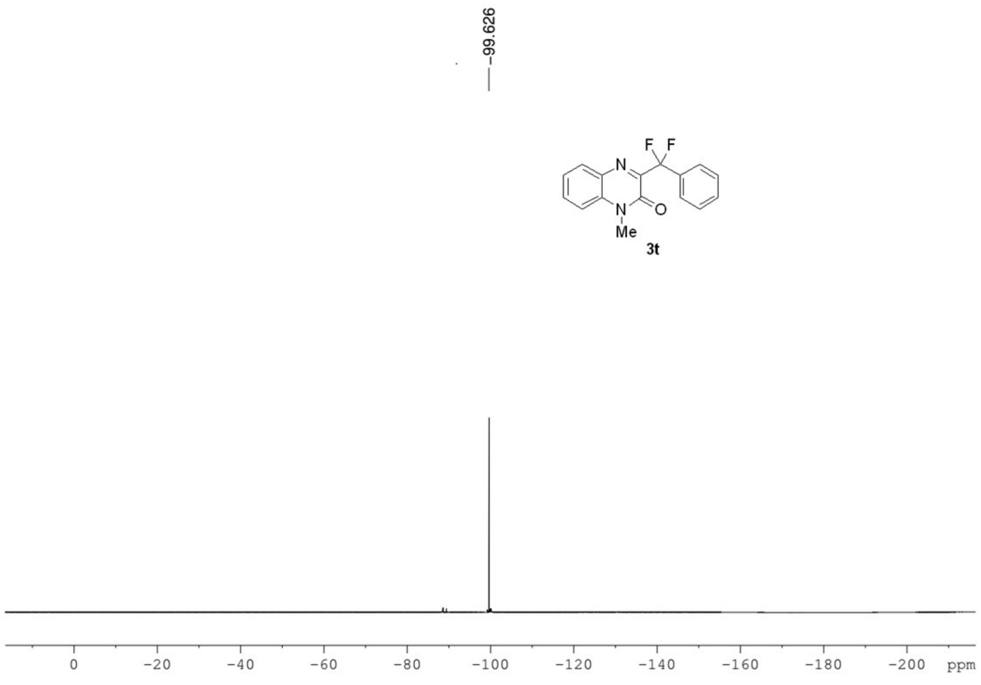
-99.626
F F
N
N
Me
O
3t
ppm

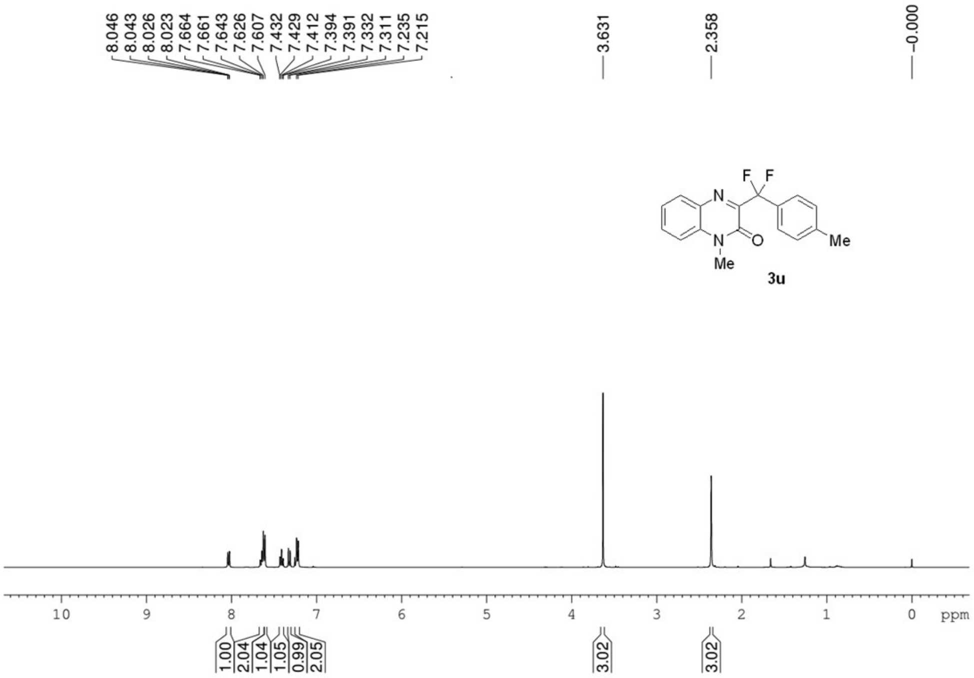
8.046
8.043
8.026
8.023
7.664
7.661
7.643
7.626
7.607
7.432
7.429
7.412
7.394
7.391
7.332
7.311
7.235
7.215
3.631
2.358
-0.000
F F
N
N
O
Me
Me
3u
10 9 8 7 6 5 4 3 2 1 0 ppm
1.00
2.04
1.04
1.05
0.99
2.05
3.02
3.02

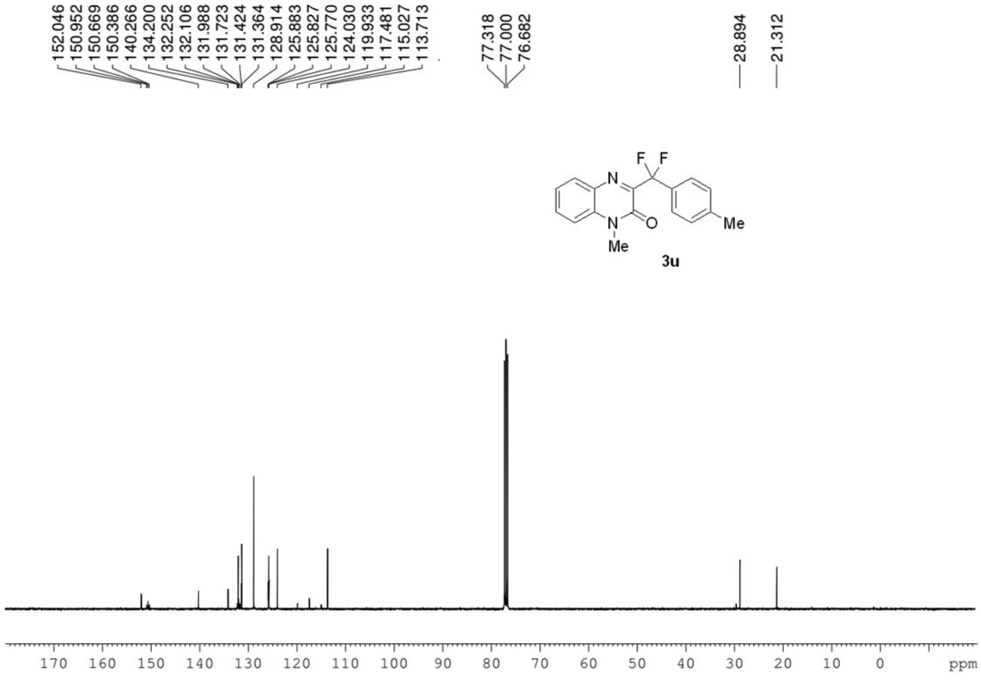
152.046
150.952
150.669
150.386
140.266
134.200
132.252
132.106
131.988
131.723
131.424
131.364
128.914
125.883
125.827
125.770
124.030
119.933
117.481
115.027
113.713
77.318
77.000
76.682
28.894
21.312
F F
N
N
O
Me
Me
3u
170 160 150 140 130 120 110 100 90 80 70 60 50 40 30 20 10 0 ppm

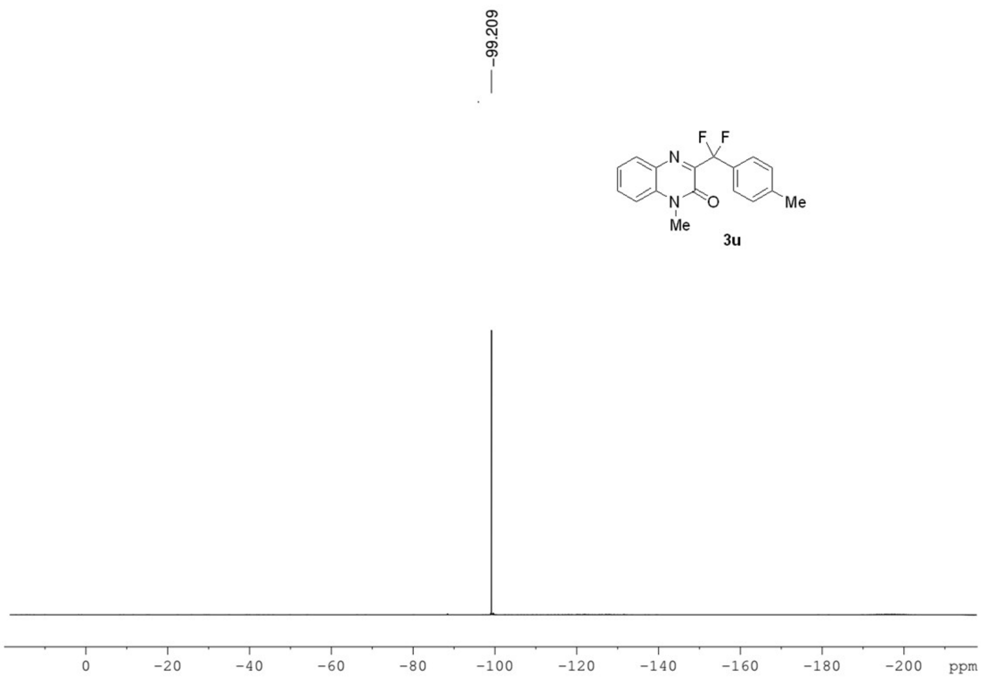
-99.209
F F
N
N
O
Me
Me
0 -20 -40 -60 -80 -100 -120 -140 -160 -180 -200 ppm

3u

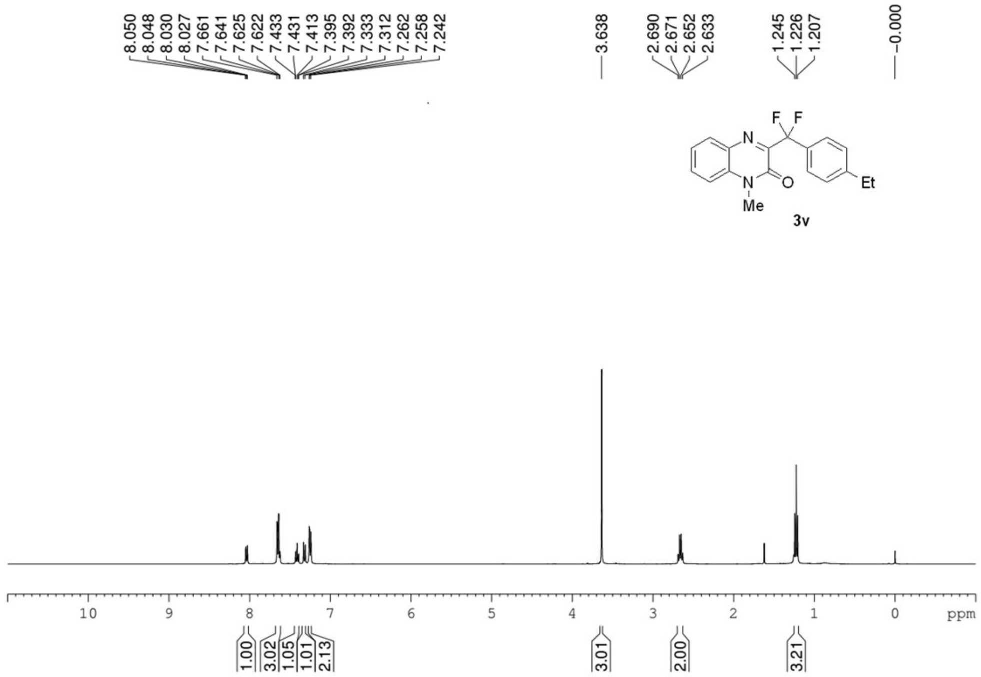
8.050
8.048
8.030
8.027
7.661
7.641
7.625
7.622
7.433
7.431
7.413
7.395
7.392
7.333
7.312
7.262
7.258
7.242
3.638
2.690
2.671
2.652
2.633
1.245
1.226
1.207
-0.000
F F
N
N
O
Me
Et
10 9 8 7 6 5 4 3 2 1 0 ppm
1.00
3.02
1.05
1.01
2.13
3.01
2.00
3.21

3v

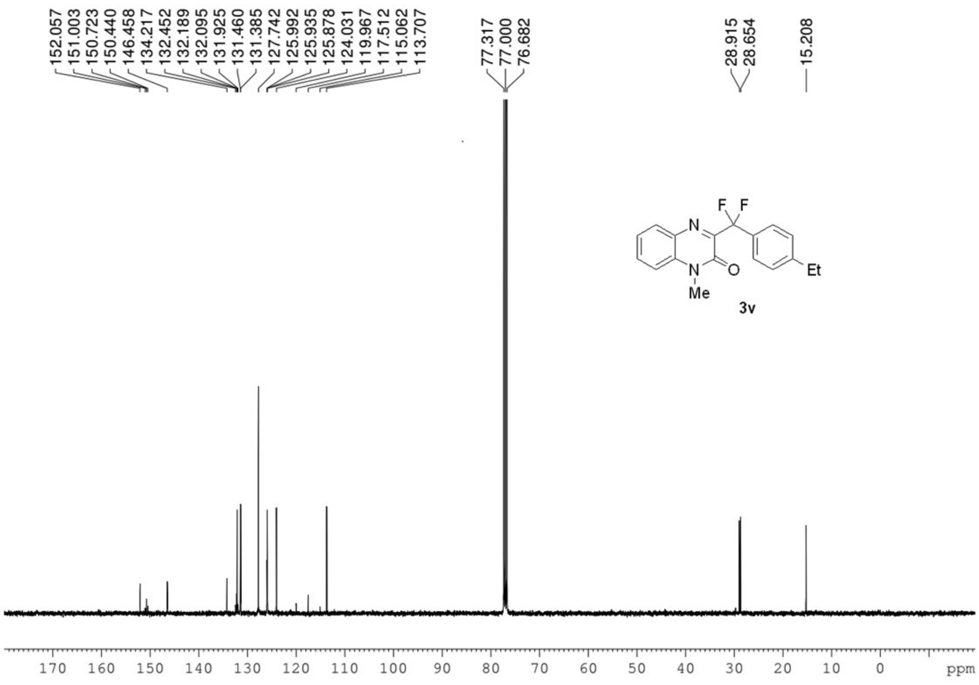
152.057
151.003
150.723
150.440
146.458
134.217
132.452
132.189
132.095
131.925
131.460
131.385
127.742
125.992
125.935
125.878
124.031
119.967
117.512
115.062
113.707
77.317
77.000
76.682
28.915
28.654
15.208
F F
N
N
Me
O
Et
3v
170 160 150 140 130 120 110 100 90 80 70 60 50 40 30 20 10 0 ppm

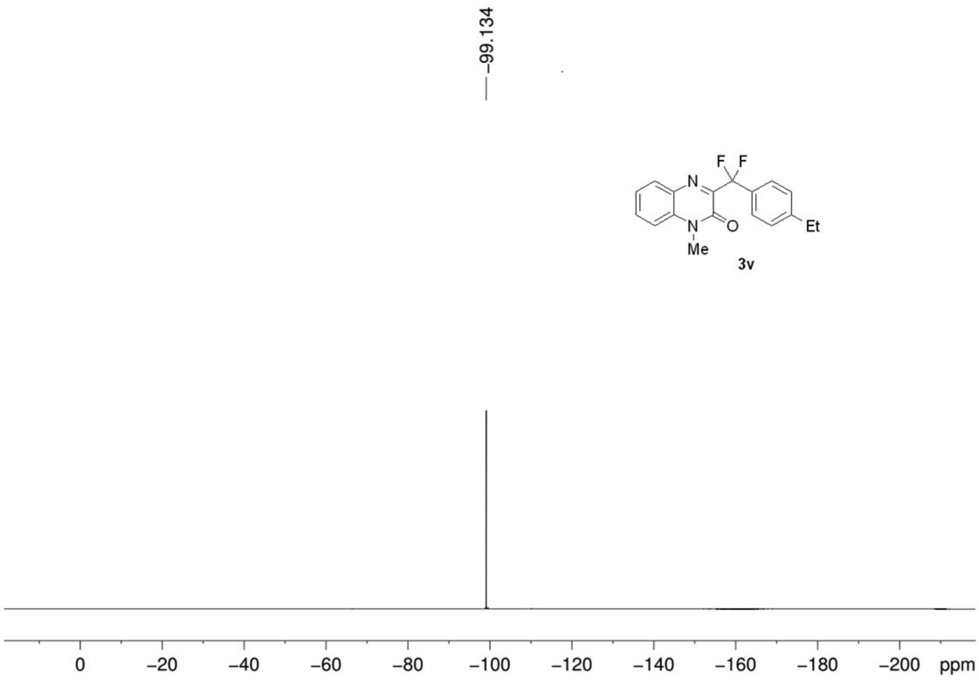
−99.134
F F
N
N
Me
O
Et
3v
0 −20 −40 −60 −80 −100 −120 −140 −160 −180 −200 ppm

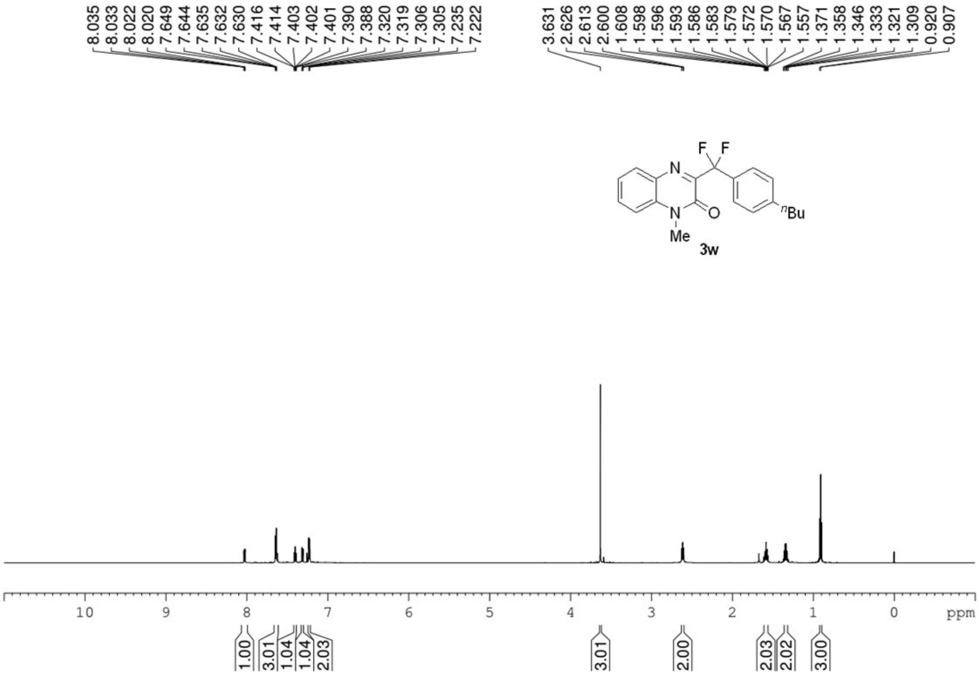
8.035 8.033 8.022 8.020 7.649 7.644 7.635 7.632 7.630 7.416 7.414 7.403 7.402 7.401 7.390 7.388 7.320 7.319 7.306 7.305 7.235 7.222
3.631 2.626 2.613 2.600 1.608 1.598 1.596 1.593 1.586 1.583 1.579 1.572 1.570 1.567 1.557 1.371 1.358 1.346 1.333 1.321 1.309 0.920 0.907
F F
N
N
O
Me
^{n}Bu
3w
10 9 8 7 6 5 4 3 2 1 0 ppm
1.00 3.01 1.04 1.04 2.03
3.01
2.00
2.03 2.02
3.00

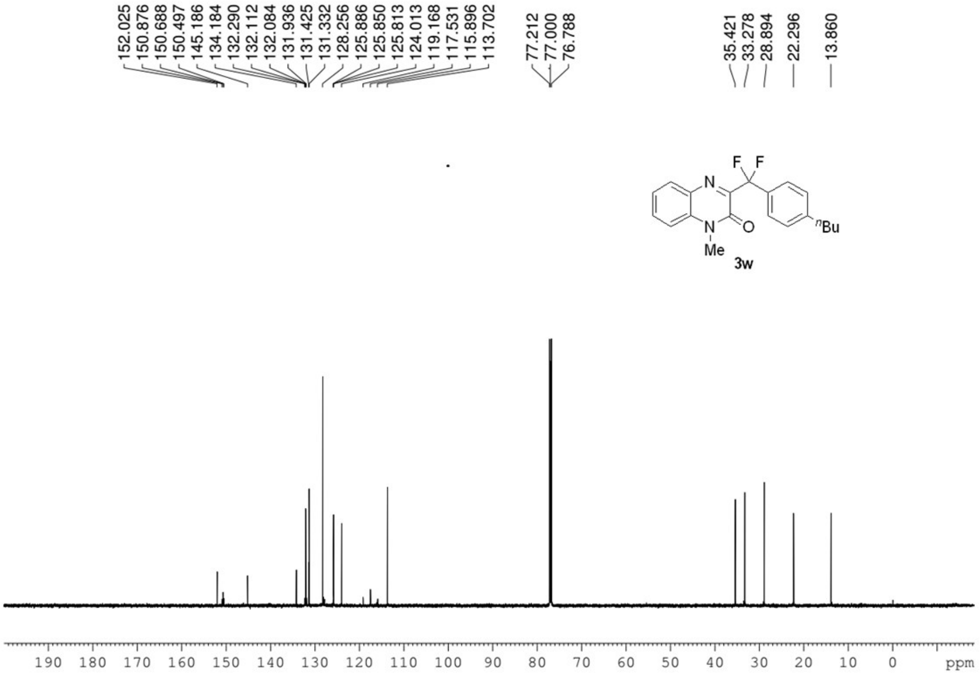
152.025 150.876 150.688 150.497 145.186 134.184 132.290 132.112 132.084 131.936 131.425 131.332 128.256 125.886 125.850 125.813 124.013 119.168 117.531 115.896 113.702
77.212 77.000 76.788
35.421 33.278 28.894 22.296 13.860
F F
N
N
O
Me
^{n}Bu
3w
190 180 170 160 150 140 130 120 110 100 90 80 70 60 50 40 30 20 10 0 ppm

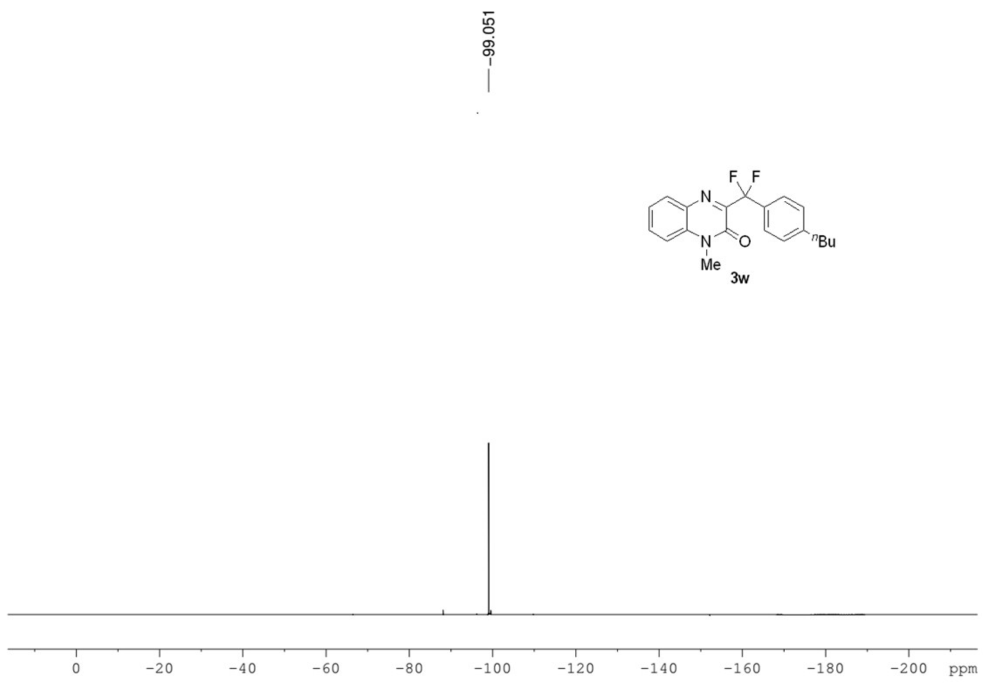

-99.051
F F
N
N
O
Me
^{n}Bu
3w
0 -20 -40 -60 -80 -100 -120 -140 -160 -180 -200 ppm

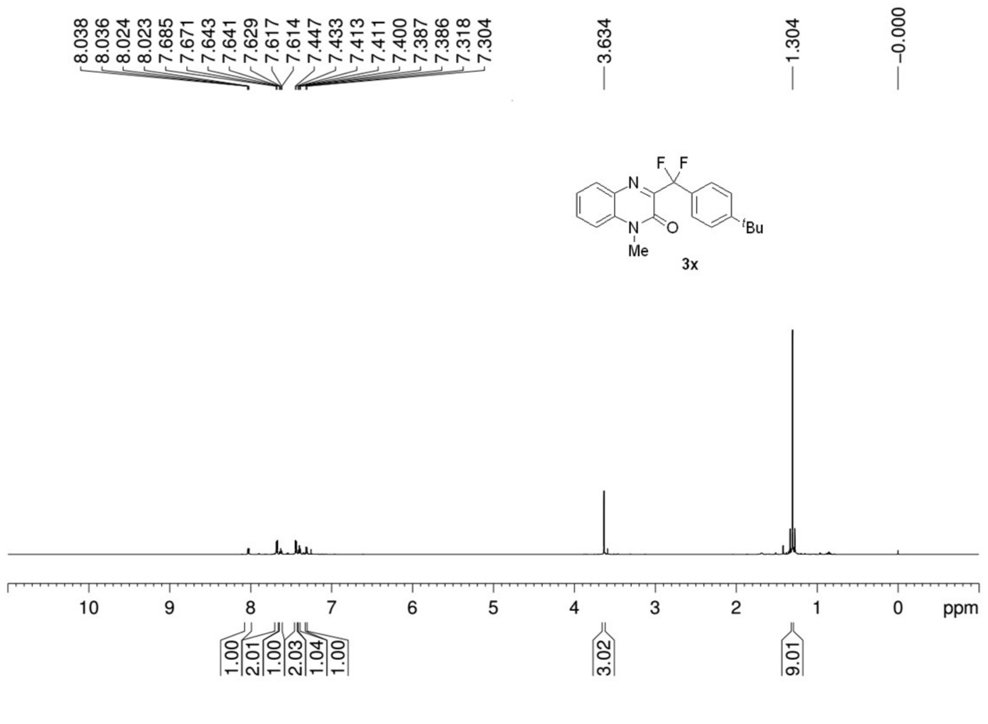

8.038 8.036 8.024 8.023 7.685 7.671 7.643 7.641 7.629 7.617 7.614 7.447 7.433 7.413 7.411 7.400 7.387 7.386 7.318 7.304
3.634
1.304
-0.000
F F
N
N
O
Me
^{t}Bu
3x
10 9 8 7 6 5 4 3 2 1 0 ppm
1.00 2.01 1.00 2.03 1.04 1.00
3.02
9.01

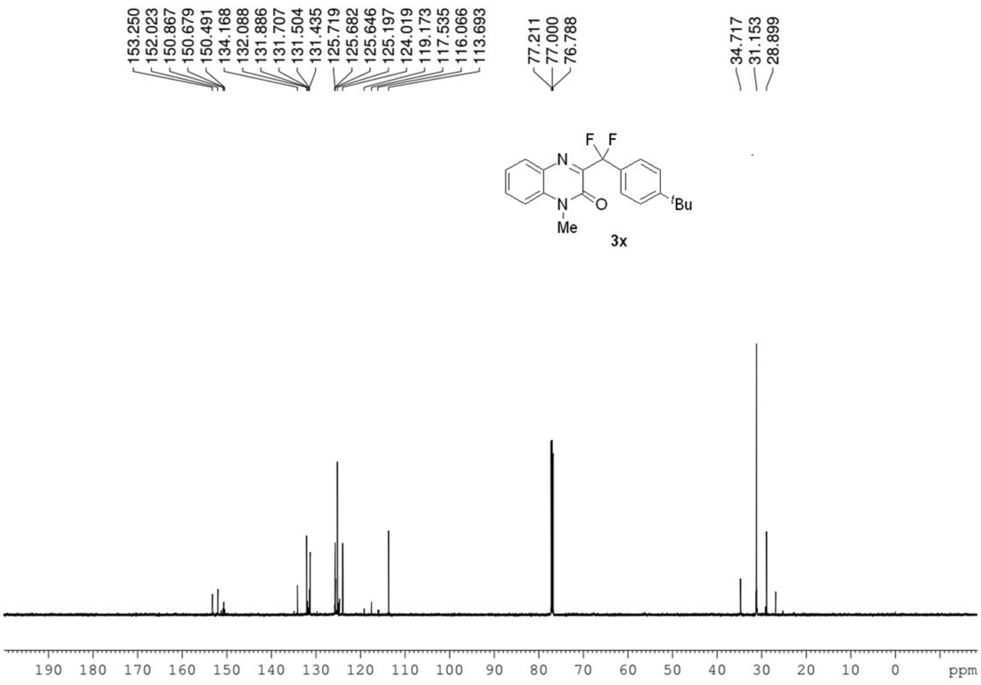
153.250
152.023
150.867
150.679
150.491
134.168
132.088
131.886
131.707
131.504
131.435
125.719
125.682
125.646
125.197
124.019
119.173
117.535
116.066
113.693
77.211
77.000
76.788
34.717
31.153
28.899
F F
N
N
O
Me
tBu
3x
190 180 170 160 150 140 130 120 110 100 90 80 70 60 50 40 30 20 10 0 ppm

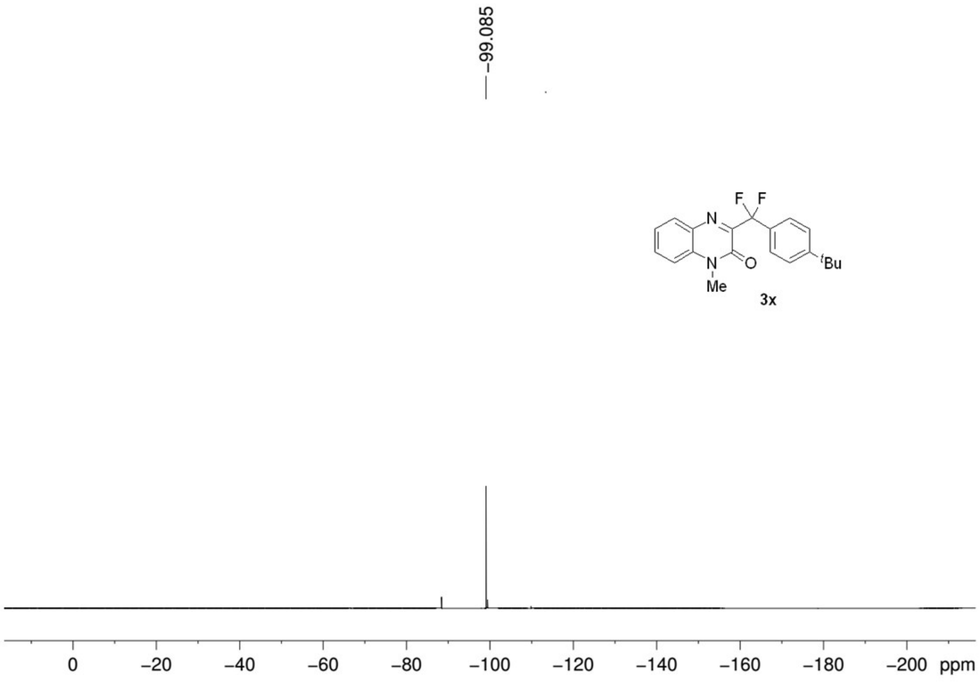
−99.085
F F
N
N
O
Me
tBu
3x
0 −20 −40 −60 −80 −100 −120 −140 −160 −180 −200 ppm

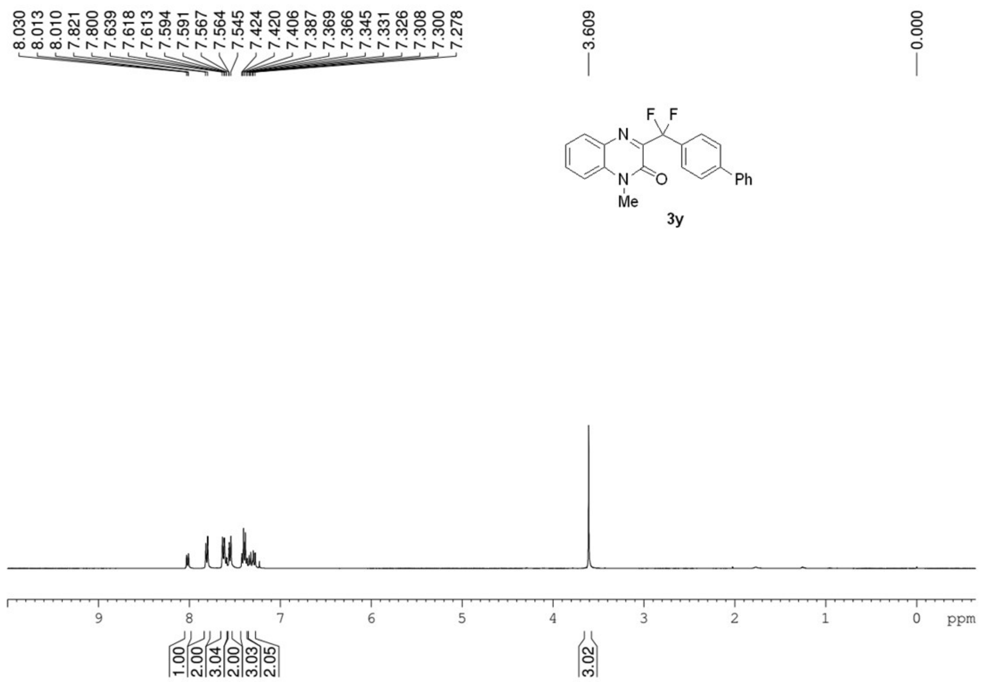
8.030
8.013
8.010
7.821
7.800
7.639
7.618
7.613
7.594
7.591
7.567
7.564
7.545
7.424
7.420
7.406
7.387
7.369
7.366
7.345
7.331
7.326
7.308
7.300
7.278
3.609
0.000
F F
N
N
O
Me
Ph
3y
9 8 7 6 5 4 3 2 1 0 ppm
1.00
2.00
3.04
2.00
3.03
2.05
3.02

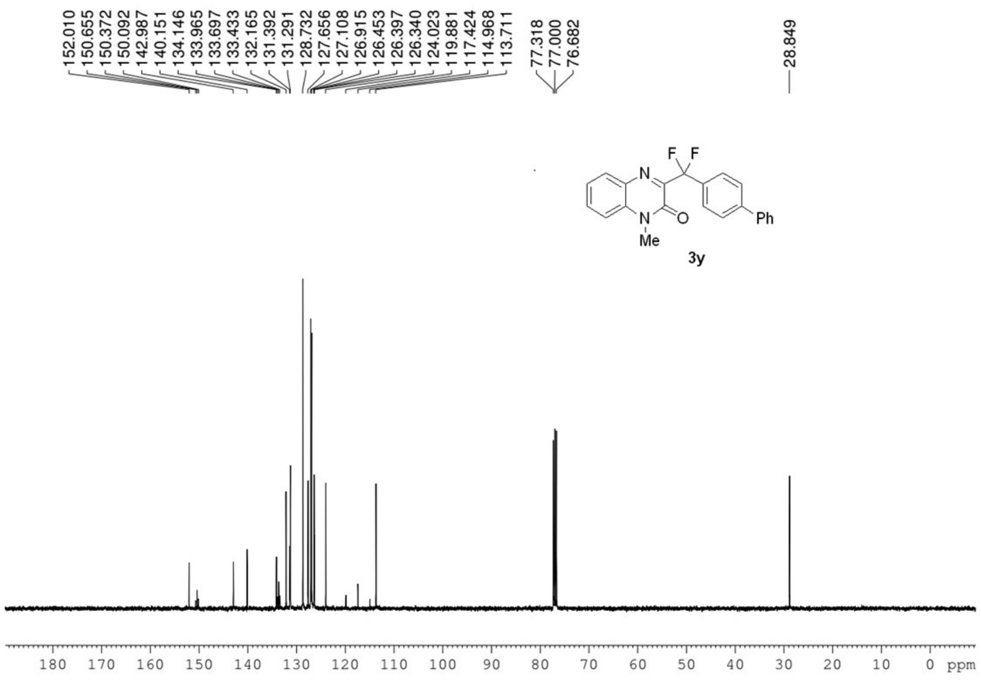
152.010
150.655
150.372
150.092
142.987
140.151
134.146
133.965
133.697
133.433
132.165
131.392
131.291
128.732
127.656
127.108
126.915
126.453
126.397
126.340
124.023
119.881
117.424
114.968
113.711
77.318
77.000
76.682
28.849
F F
N
N
O
Me
Ph
3y
180 170 160 150 140 130 120 110 100 90 80 70 60 50 40 30 20 10 0 ppm

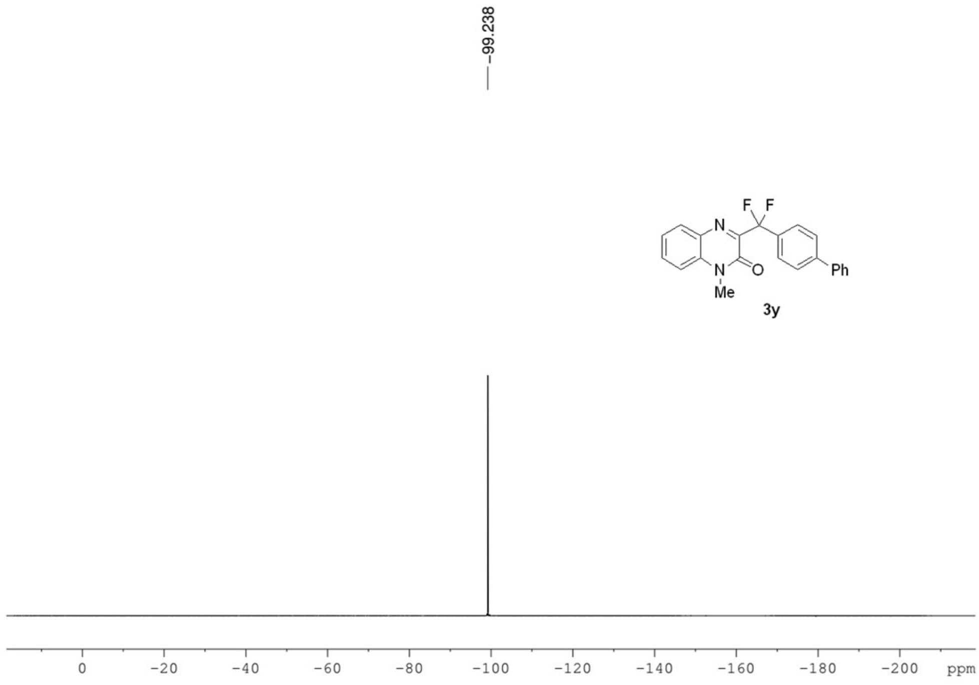

−99.238
F F
N
N
O
Me
Ph
3y
0 −20 −40 −60 −80 −100 −120 −140 −160 −180 −200 ppm

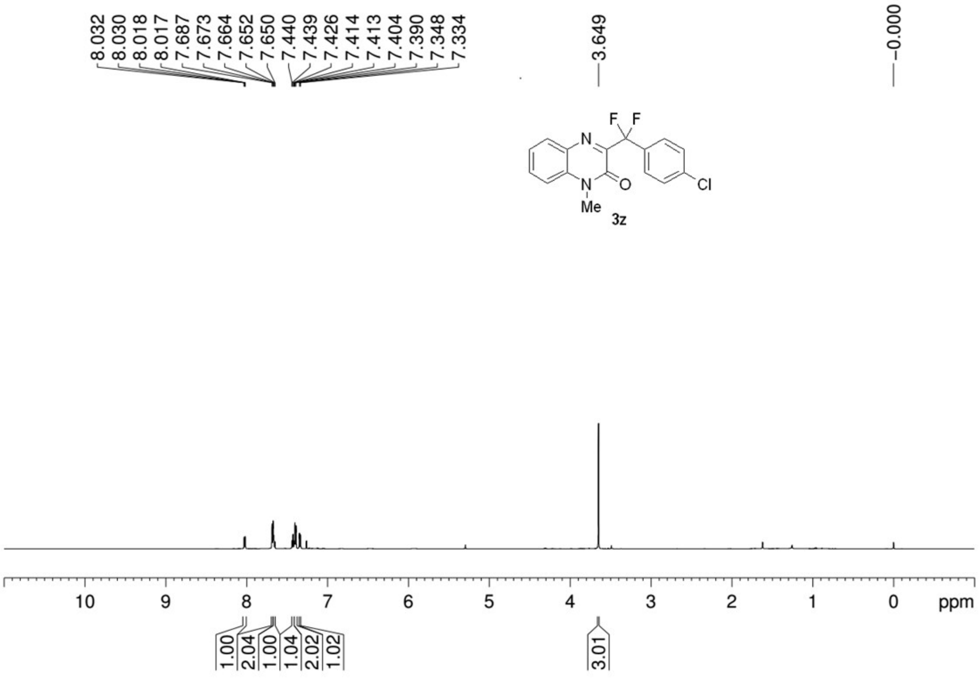

8.032 8.030 8.018 8.017 7.687 7.673 7.664 7.652 7.650 7.440 7.439 7.426 7.414 7.413 7.404 7.390 7.348 7.334
3.649
0.000
F F
N
N
O
Me
Cl
3z
10 9 8 7 6 5 4 3 2 1 0 ppm
1.00 2.04 1.00 1.04 2.02 1.02
3.01

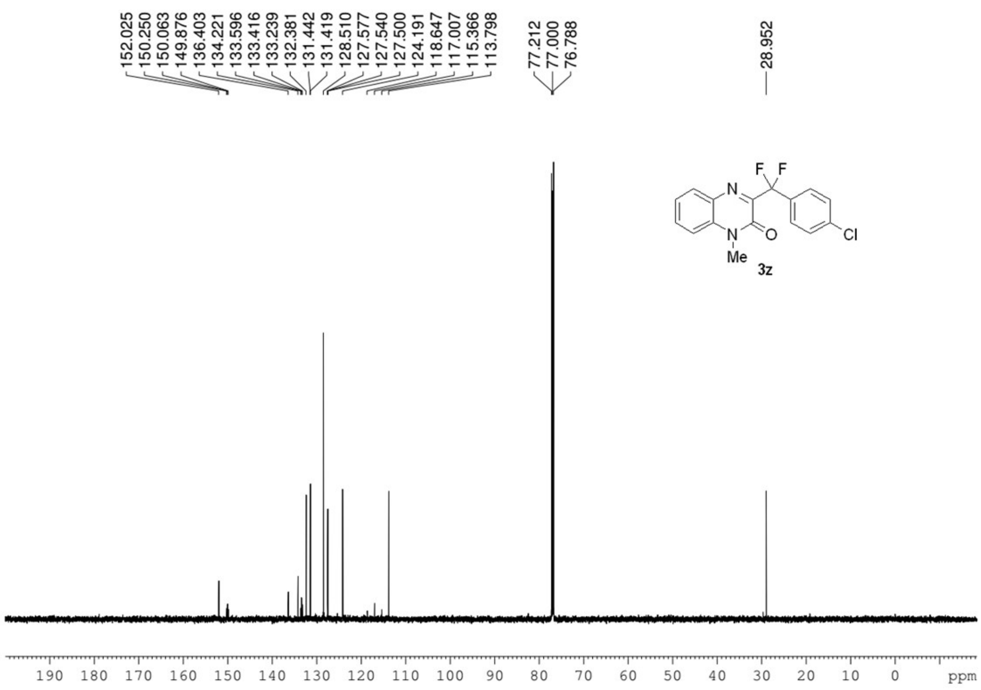
152.025
150.250
150.063
149.876
136.403
134.221
133.596
133.416
133.239
132.381
131.442
131.419
128.510
127.577
127.540
127.500
124.191
118.647
117.007
115.366
113.798
77.212
77.000
76.788
28.952
F
F
N
O
N
Me
Cl
3z
190 180 170 160 150 140 130 120 110 100 90 80 70 60 50 40 30 20 10 0 ppm

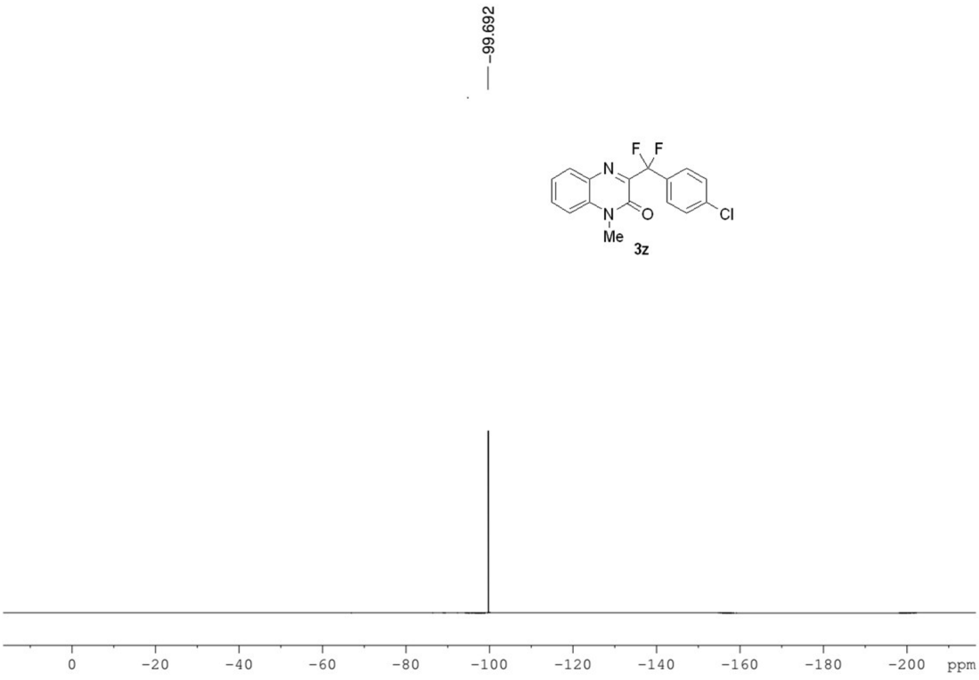
-99.692
F
F
N
O
N
Me
Cl
3z
0 -20 -40 -60 -80 -100 -120 -140 -160 -180 -200 ppm

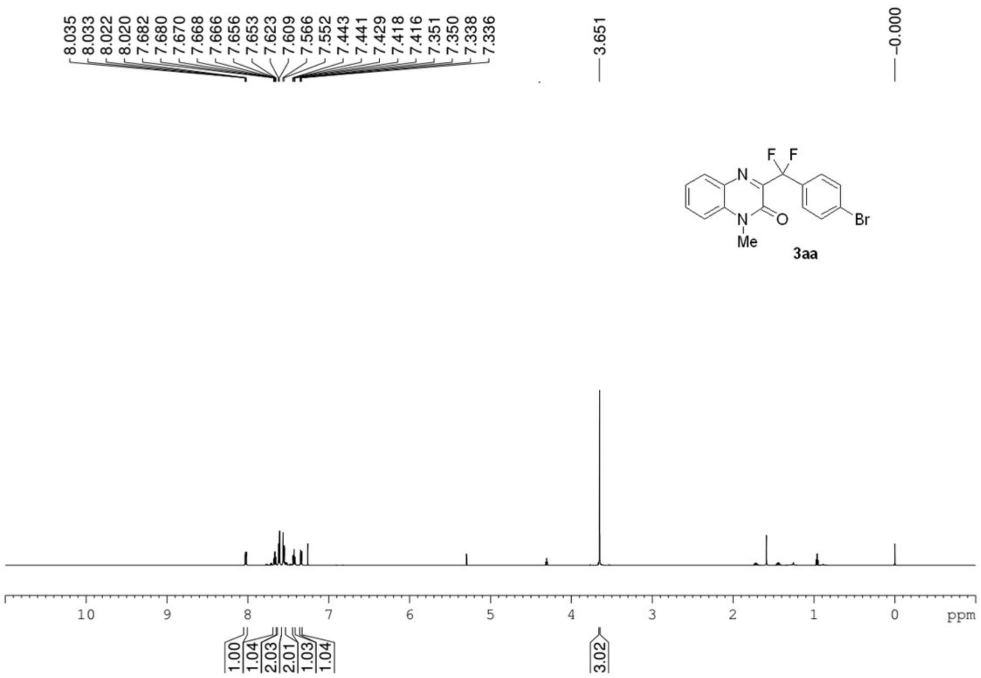
8.035
8.033
8.022
8.020
7.682
7.680
7.670
7.668
7.666
7.656
7.653
7.623
7.609
7.566
7.552
7.443
7.441
7.429
7.418
7.416
7.351
7.350
7.338
7.336
3.651
-0.000
N
F F
N
O
Br
Me
3aa
10 9 8 7 6 5 4 3 2 1 0 ppm
1.00
1.04
2.03
2.01
1.03
1.04
3.02

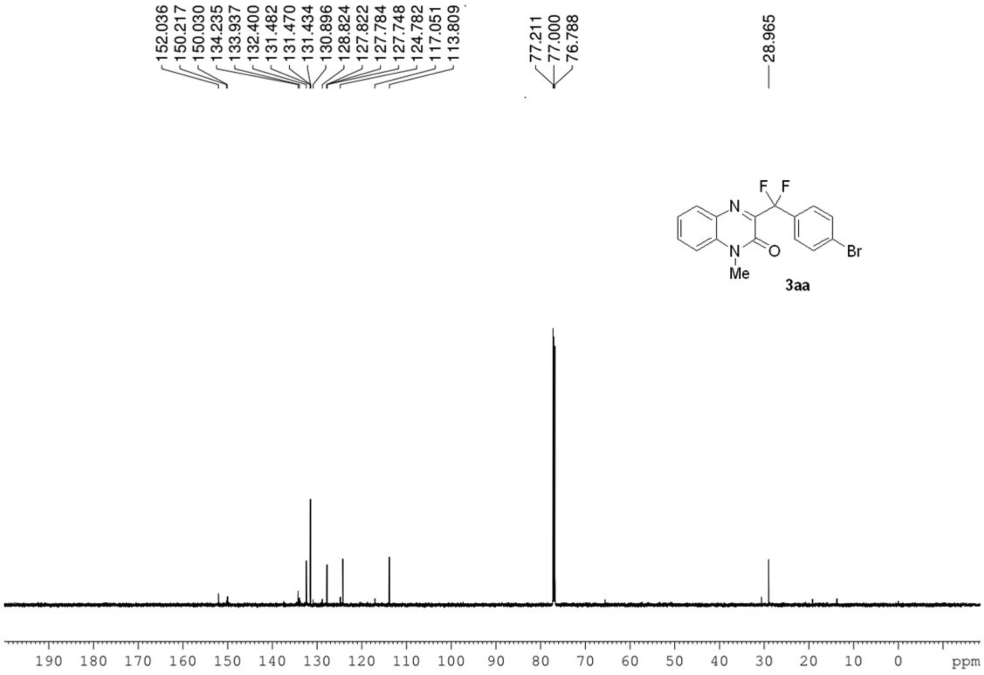
152.036
150.217
150.030
134.235
133.937
132.400
131.482
131.470
131.434
130.896
128.824
127.822
127.784
127.748
124.782
117.051
113.809
77.211
77.000
76.788
28.965
N
F F
N
O
Br
Me
3aa
190 180 170 160 150 140 130 120 110 100 90 80 70 60 50 40 30 20 10 0 ppm

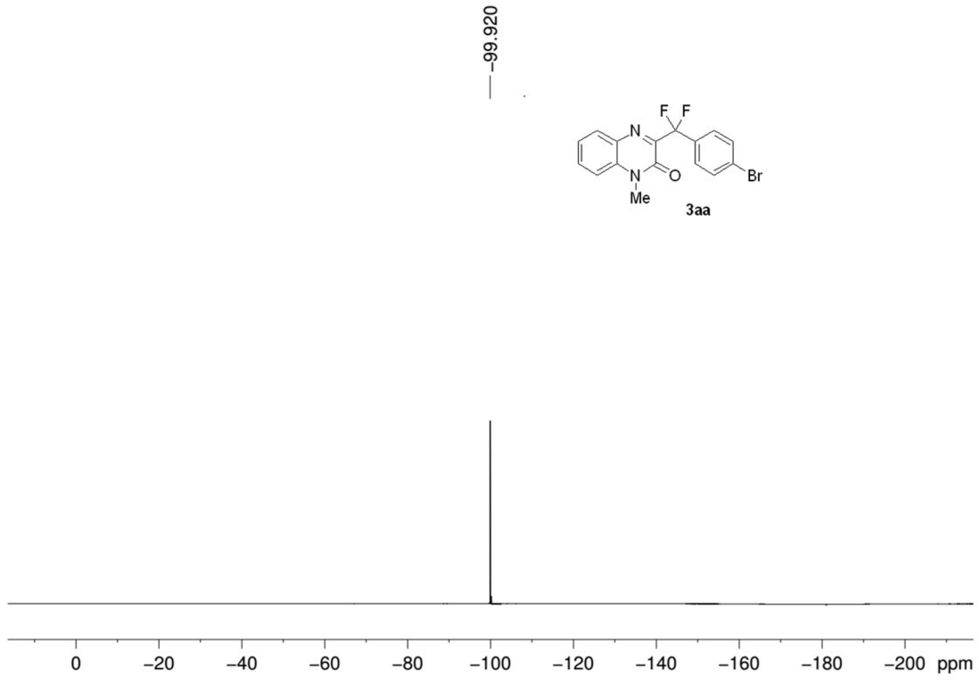
-99.920
F F
N
N
O
Me
Br
3aa
0
-20
-40
-60
-80
-100
-120
-140
-160
-180
-200 ppm

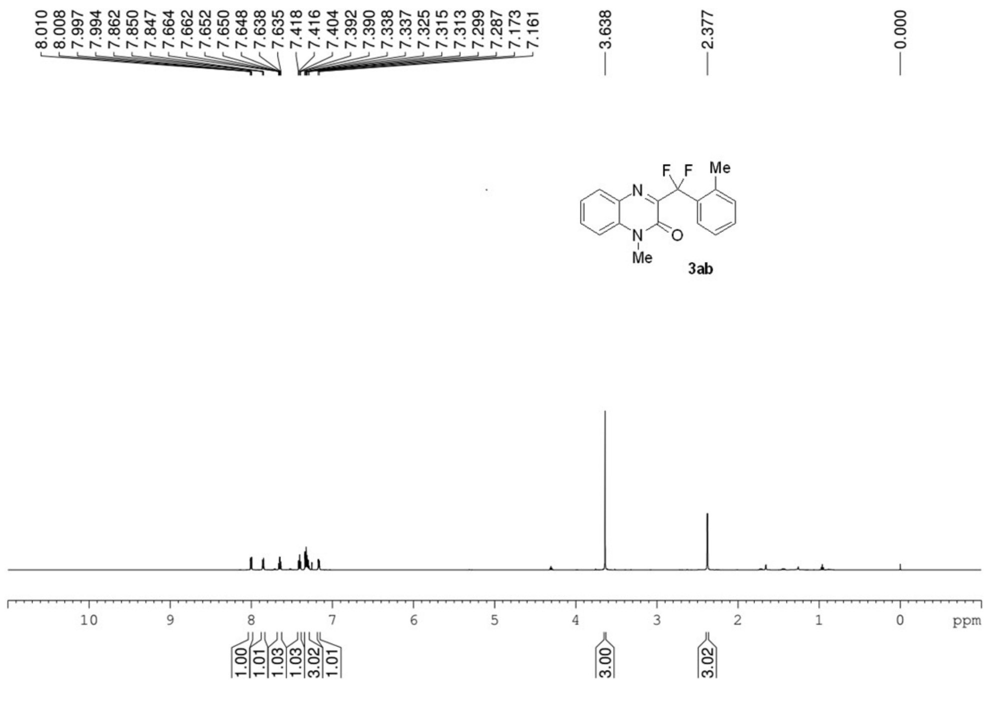
8.010
8.008
7.997
7.994
7.862
7.850
7.847
7.664
7.662
7.652
7.650
7.648
7.638
7.635
7.418
7.416
7.404
7.392
7.390
7.338
7.337
7.325
7.315
7.313
7.299
7.287
7.173
7.161
3.638
2.377
0.000
F F
Me
N
N
O
Me
3ab
10
9
8
7
6
5
4
3
2
1
0
ppm
1.00
1.01
1.03
1.03
3.02
1.01
3.00
3.02

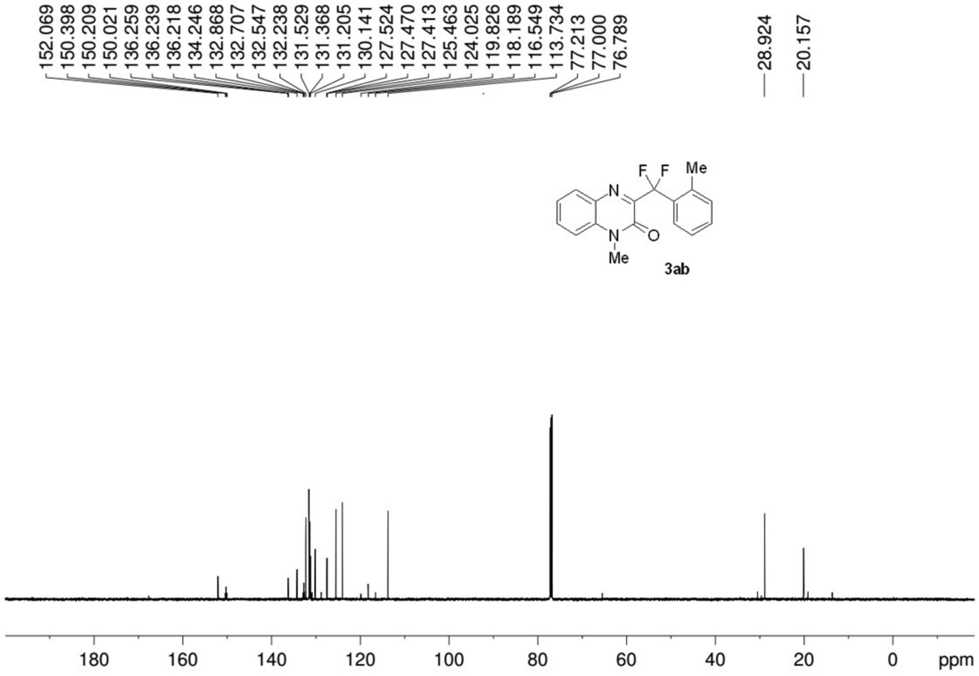
152.069
150.398
150.209
150.021
136.259
136.239
136.218
134.246
132.868
132.707
132.547
132.238
131.529
131.368
131.205
130.141
127.524
127.470
127.413
125.463
124.025
119.826
118.189
116.549
113.734
77.213
77.000
76.789
28.924
20.157
F F Me
N
N
O
Me
3ab
180 160 140 120 100 80 60 40 20 0 ppm

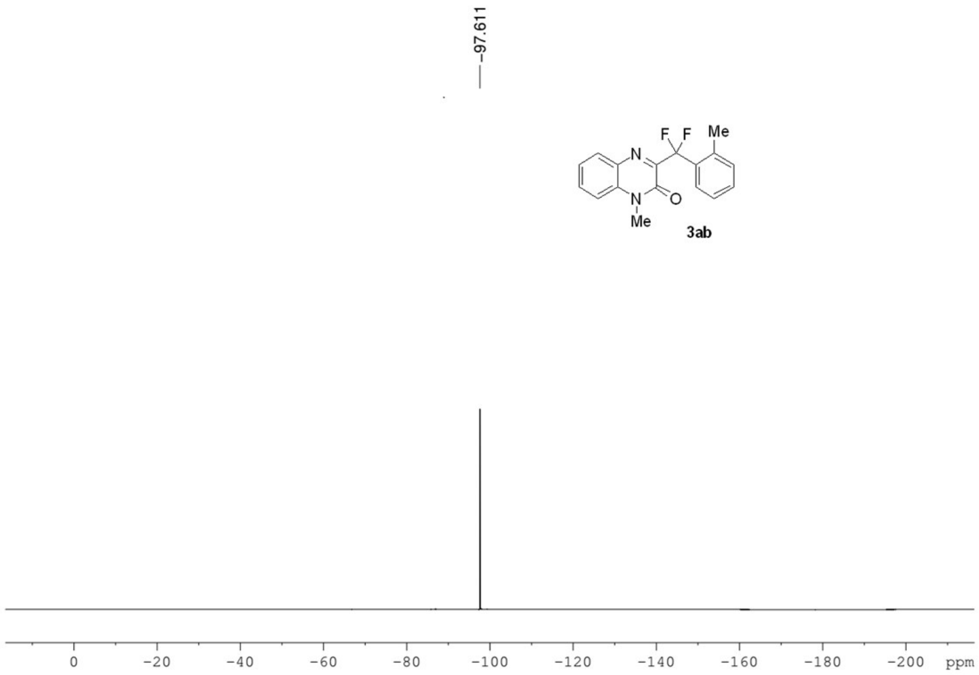
-97.611
F F Me
N
N
O
Me
3ab
0 -20 -40 -60 -80 -100 -120 -140 -160 -180 -200 ppm

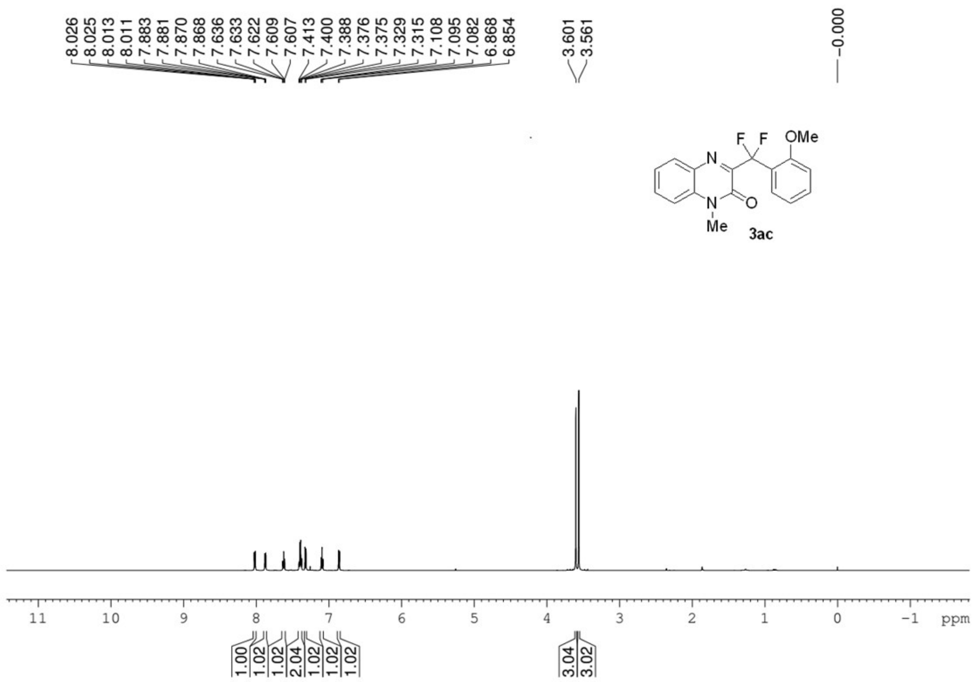
8.026
8.025
8.013
8.011
7.883
7.881
7.870
7.868
7.636
7.633
7.622
7.609
7.607
7.413
7.400
7.388
7.376
7.375
7.329
7.315
7.108
7.095
7.082
6.868
6.854
3.601
3.561
-0.000
F F OMe
N
O
N
Me
3ac
11 10 9 8 7 6 5 4 3 2 1 0 -1 ppm
1.00 1.02 1.02 2.04 1.02 1.02 1.02
3.04 3.02

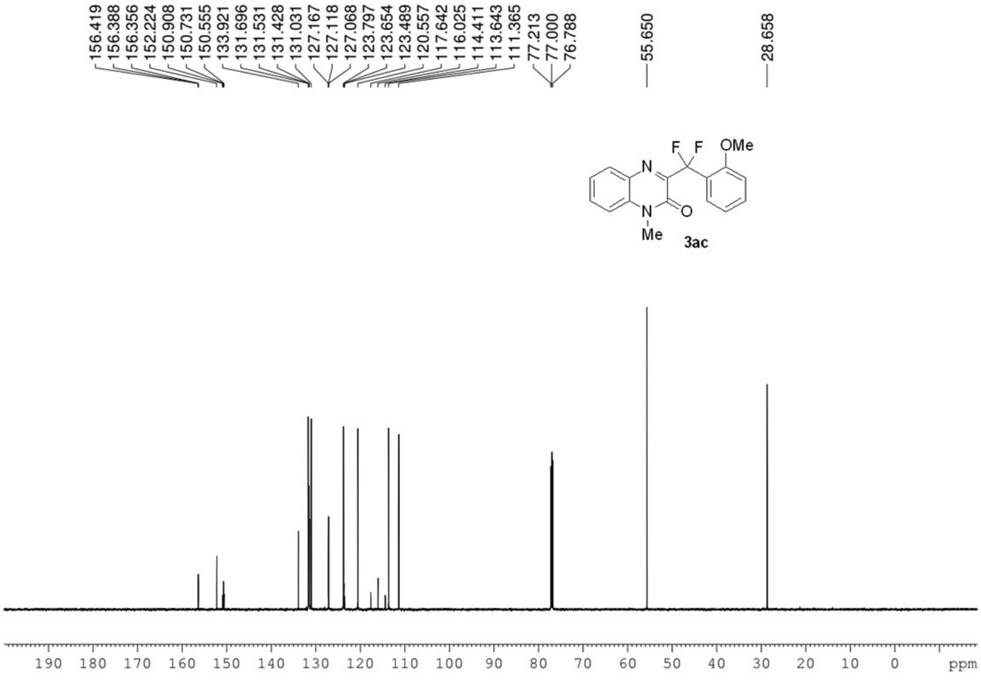
156.419
156.388
156.356
152.224
150.908
150.731
150.555
133.921
131.696
131.531
131.428
131.031
127.167
127.118
127.068
123.797
123.654
123.489
120.557
117.642
116.025
114.411
113.643
111.365
77.213
77.000
76.788
55.650
28.658
F F OMe
N
O
N
Me
3ac
190 180 170 160 150 140 130 120 110 100 90 80 70 60 50 40 30 20 10 0 ppm

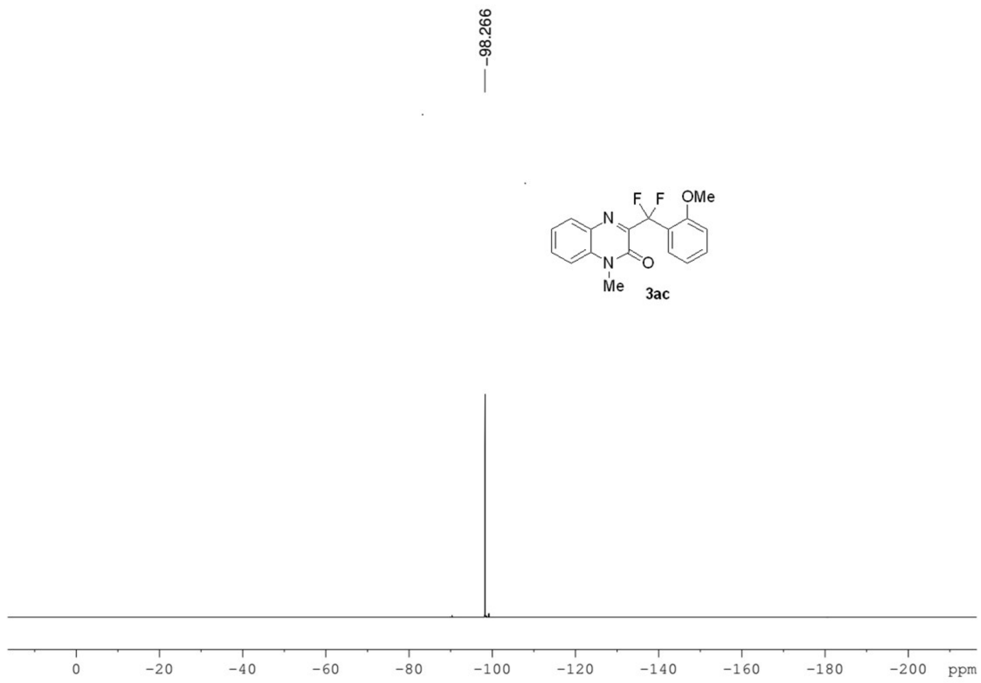
-98.266
F F OMe
N
N
O
Me
3ac
0 -20 -40 -60 -80 -100 -120 -140 -160 -180 -200 ppm

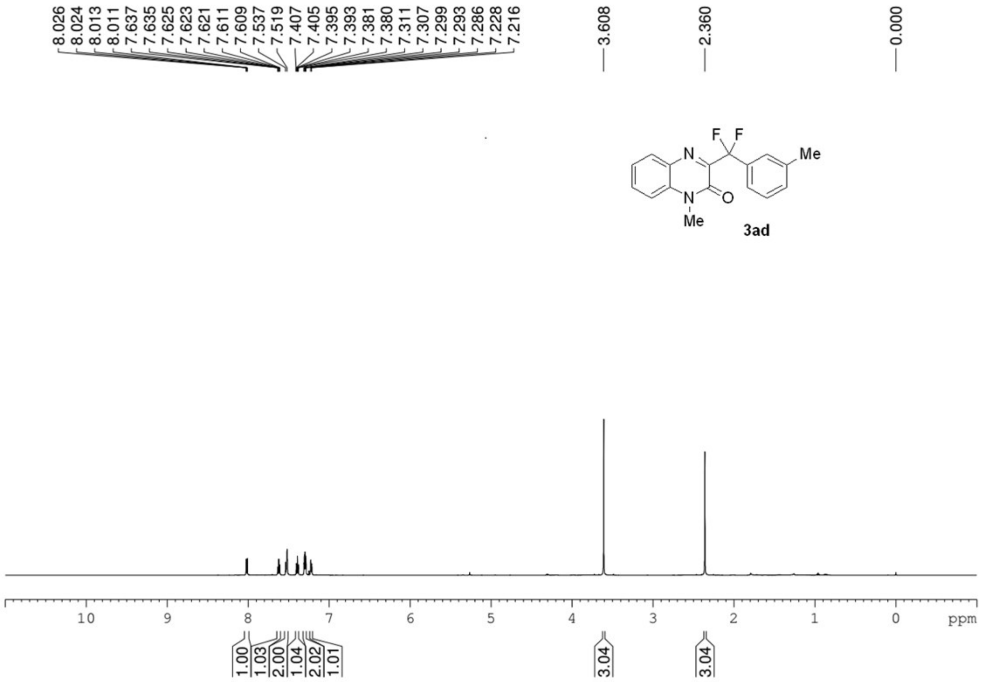
8.026
8.024
8.013
8.011
7.637
7.635
7.625
7.623
7.621
7.611
7.609
7.537
7.519
7.407
7.405
7.395
7.393
7.381
7.380
7.311
7.307
7.299
7.293
7.286
7.228
7.216
3.608
2.360
0.000
F F
Me
N
N
O
Me
3ad
10 9 8 7 6 5 4 3 2 1 0 ppm
1.00
1.03
2.00
1.04
2.02
1.01
3.04
3.04

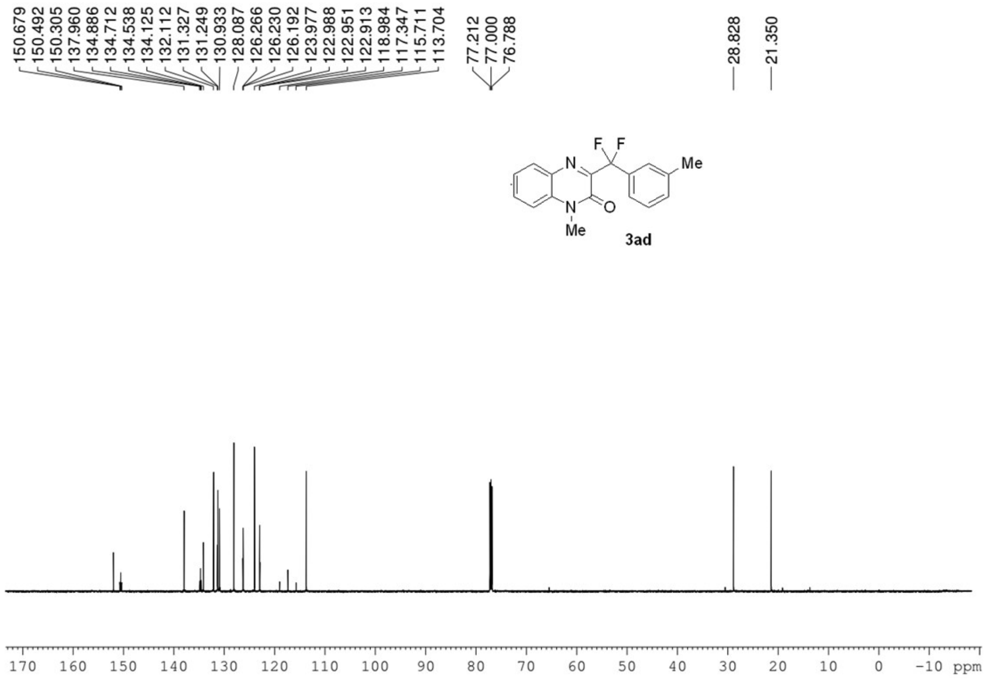
150.679
150.492
150.305
137.960
134.886
134.712
134.538
134.125
132.112
131.327
131.249
130.933
128.087
126.266
126.230
126.192
123.977
122.988
122.951
122.913
118.984
117.347
115.711
113.704
77.212
77.000
76.788
28.828
21.350
F F
N
Me
O
N
Me
3ad
170 160 150 140 130 120 110 100 90 80 70 60 50 40 30 20 10 0 −10 ppm

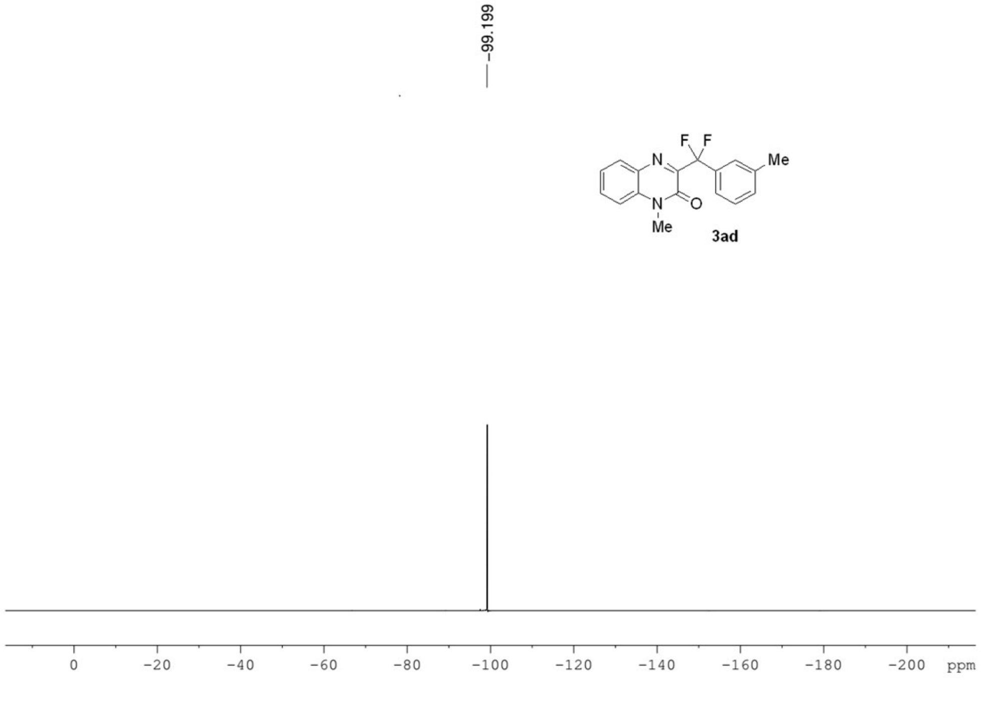
−99.199
F F
N
Me
O
N
Me
3ad
0 −20 −40 −60 −80 −100 −120 −140 −160 −180 −200 ppm

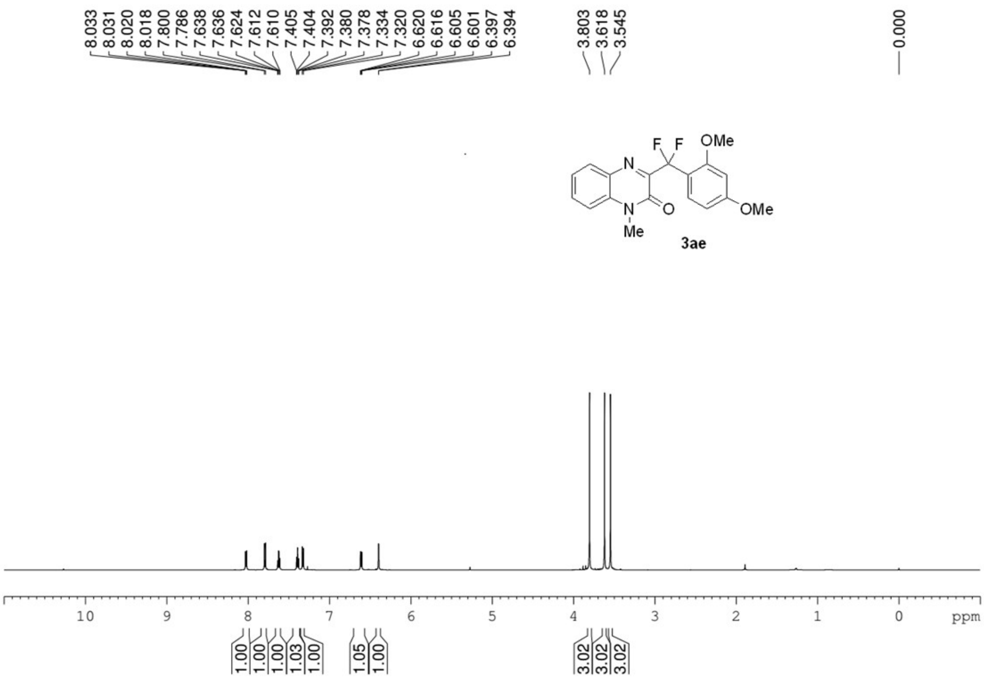
8.033
8.031
8.020
8.018
7.800
7.786
7.638
7.636
7.624
7.612
7.610
7.405
7.404
7.392
7.380
7.378
7.334
7.320
6.620
6.616
6.605
6.601
6.397
6.394
3.803
3.618
3.545
0.000
F
F
OMe
N
N
O
Me
OMe
3ae
10
9
8
7
6
5
4
3
2
1
0
ppm
1.00
1.00
1.00
1.03
1.00
1.05
1.00
3.02
3.02
3.02

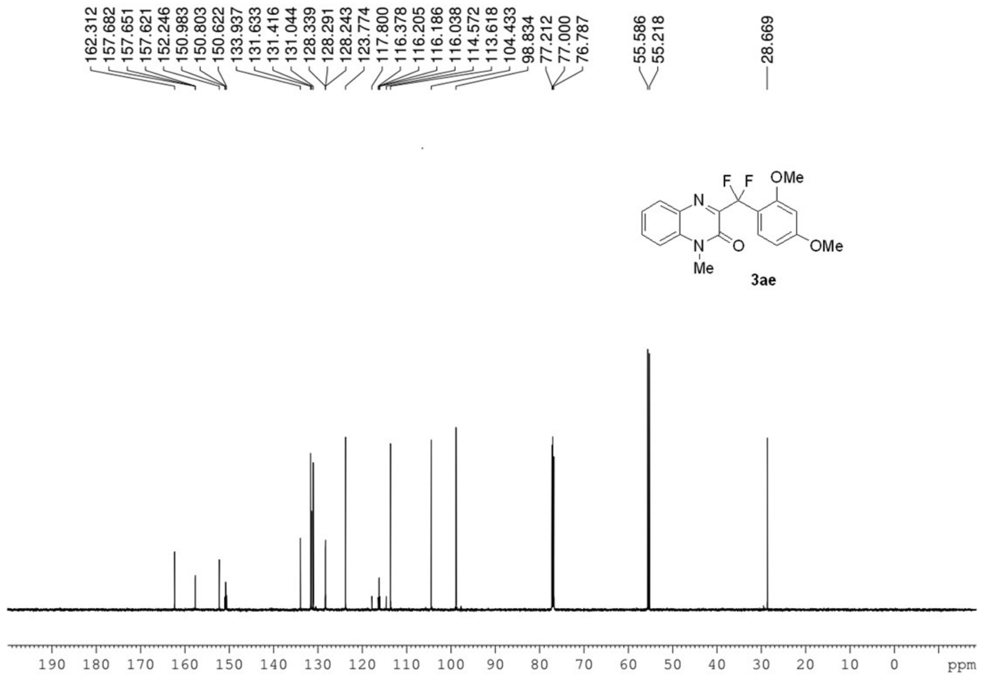
162.312
157.682
157.651
157.621
152.246
150.983
150.803
150.622
133.937
131.633
131.416
131.044
128.339
128.291
128.243
123.774
117.800
116.378
116.205
116.186
116.038
114.572
113.618
104.433
98.834
77.212
77.000
76.787
55.586
55.218
28.669
F
F
OMe
N
N
O
Me
OMe
3ae
190
180
170
160
150
140
130
120
110
100
90
80
70
60
50
40
30
20
10
0
ppm

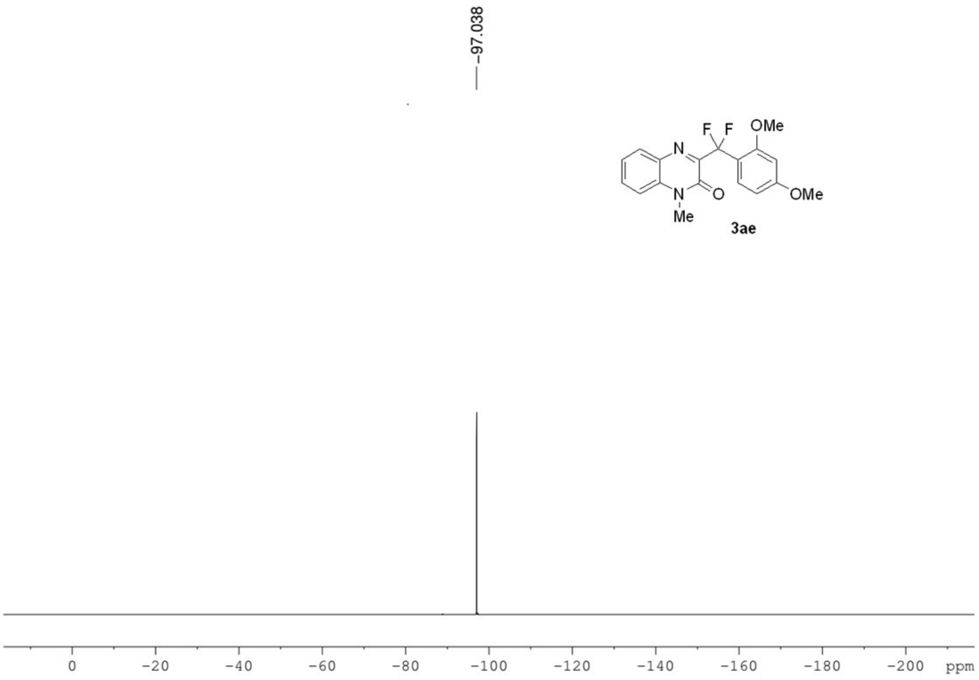

−97.038
F F
OMe
N
N
Me
O
OMe
3ae
0 −20 −40 −60 −80 −100 −120 −140 −160 −180 −200 ppm

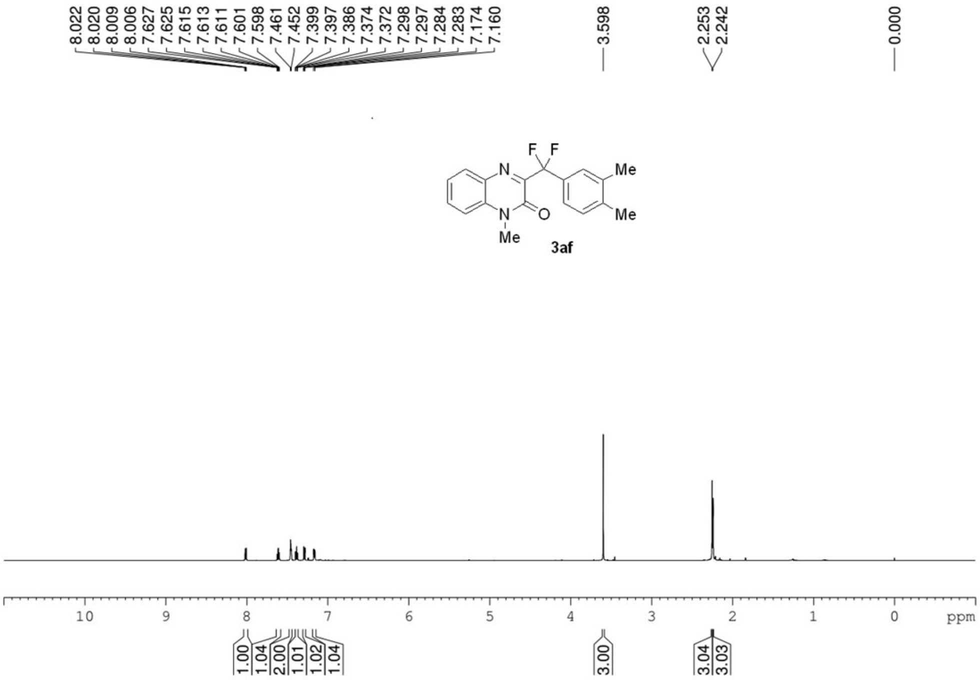

8.022 8.020 8.009 8.006 7.627 7.625 7.615 7.613 7.611 7.601 7.598 7.461 7.452 7.399 7.397 7.386 7.374 7.372 7.298 7.297 7.284 7.283 7.174 7.160
3.598
2.253 2.242
0.000
F F
Me
N
N
Me
O
Me
3af
10 9 8 7 6 5 4 3 2 1 0 ppm
1.00 1.04 2.00 1.01 1.02 1.04
3.00
3.04 3.03

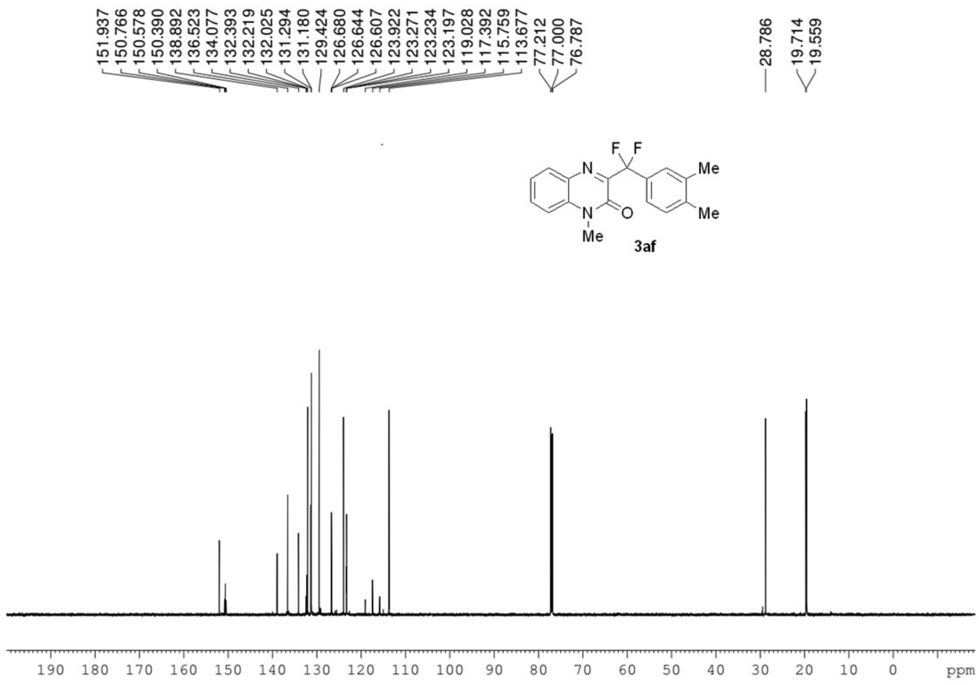
151.937
150.766
150.578
150.390
138.892
136.523
134.077
132.393
132.219
132.025
131.294
131.180
129.424
126.680
126.644
126.607
123.922
123.271
123.234
123.197
119.028
117.392
115.759
113.677
77.212
77.000
76.787
28.786
19.714
19.559
3af
190 180 170 160 150 140 130 120 110 100 90 80 70 60 50 40 30 20 10 0 ppm

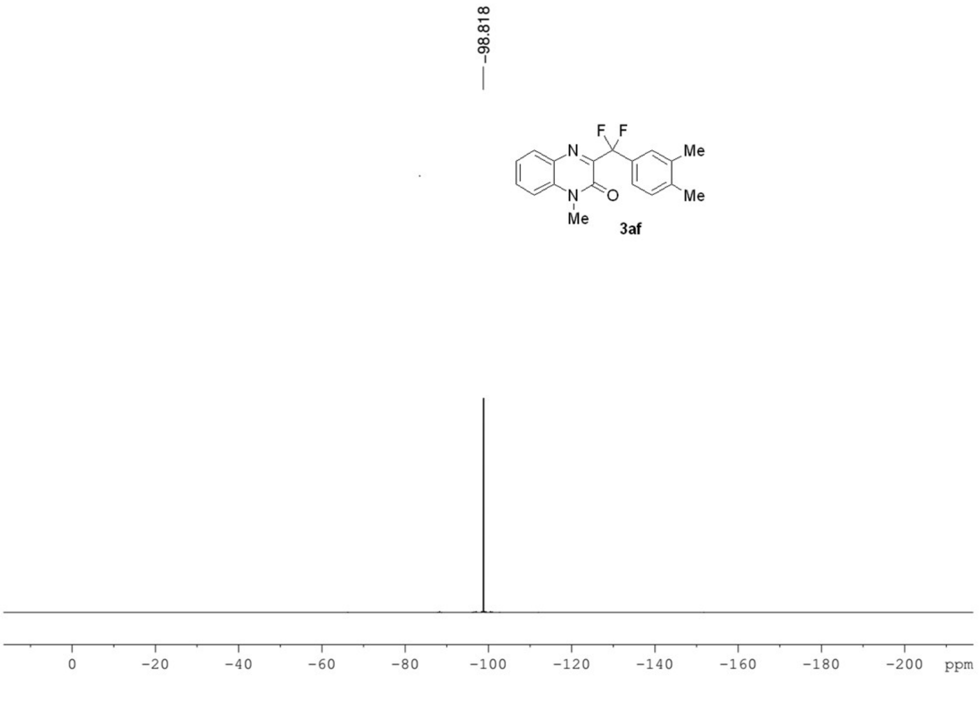
-98.818
3af
0 -20 -40 -60 -80 -100 -120 -140 -160 -180 -200 ppm

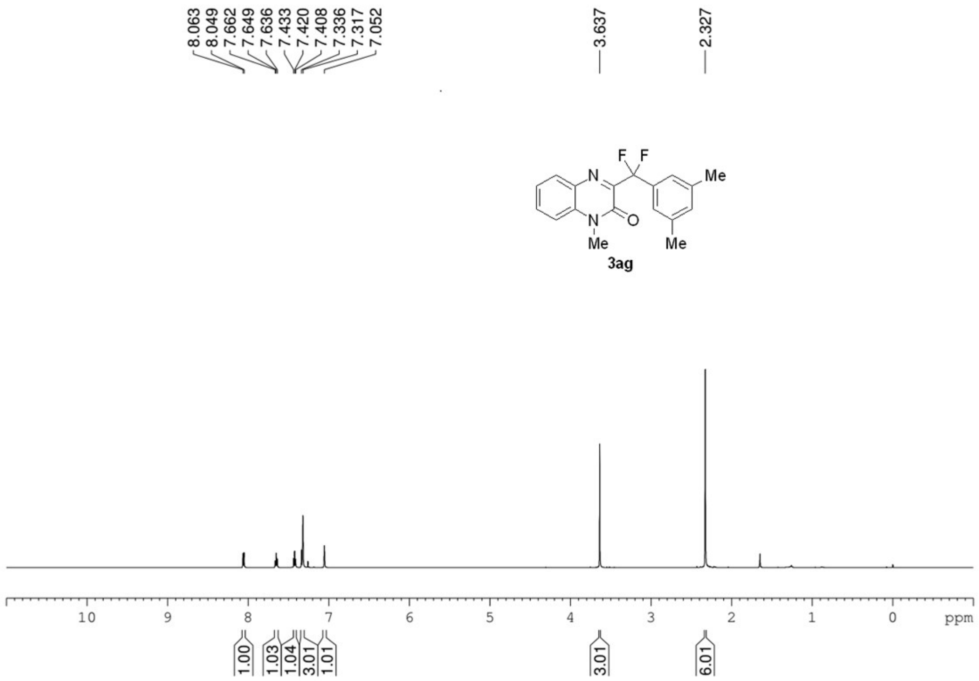
8.063
8.049
7.662
7.649
7.636
7.433
7.420
7.408
7.336
7.317
7.052
3.637
2.327
3ag
10 9 8 7 6 5 4 3 2 1 0 ppm
1.00
1.03
1.04
3.01
1.01
3.01
6.01

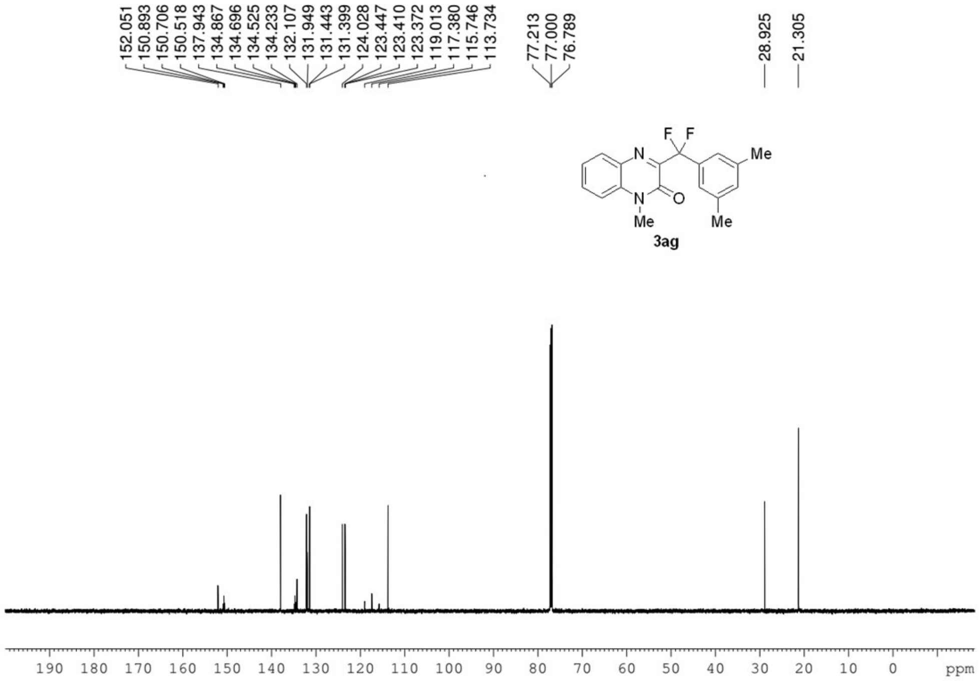
152.051
150.893
150.706
150.518
137.943
134.867
134.696
134.525
134.233
132.107
131.949
131.443
131.399
124.028
123.447
123.410
123.372
119.013
117.380
115.746
113.734
77.213
77.000
76.789
28.925
21.305
3ag
190 180 170 160 150 140 130 120 110 100 90 80 70 60 50 40 30 20 10 0 ppm

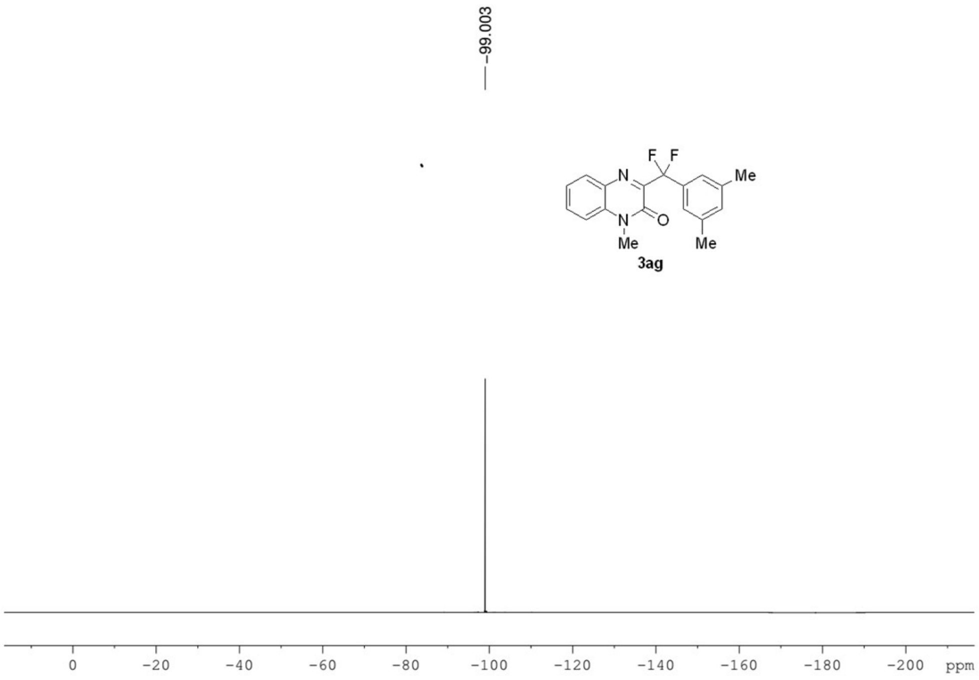

3ag

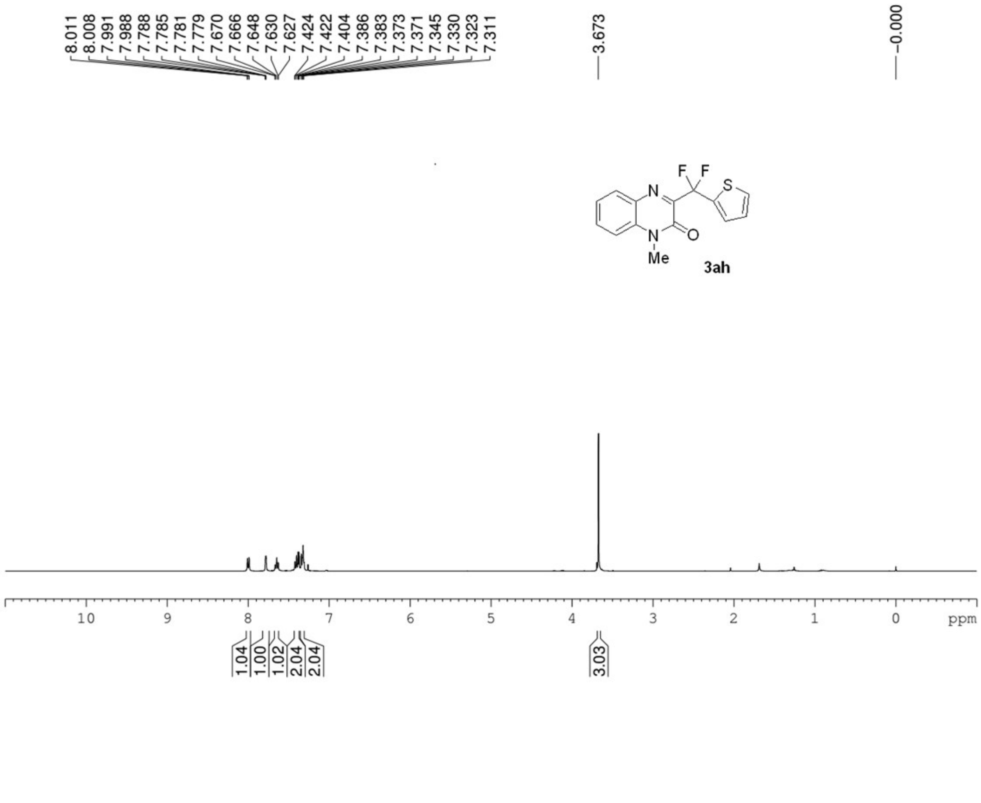

3ah

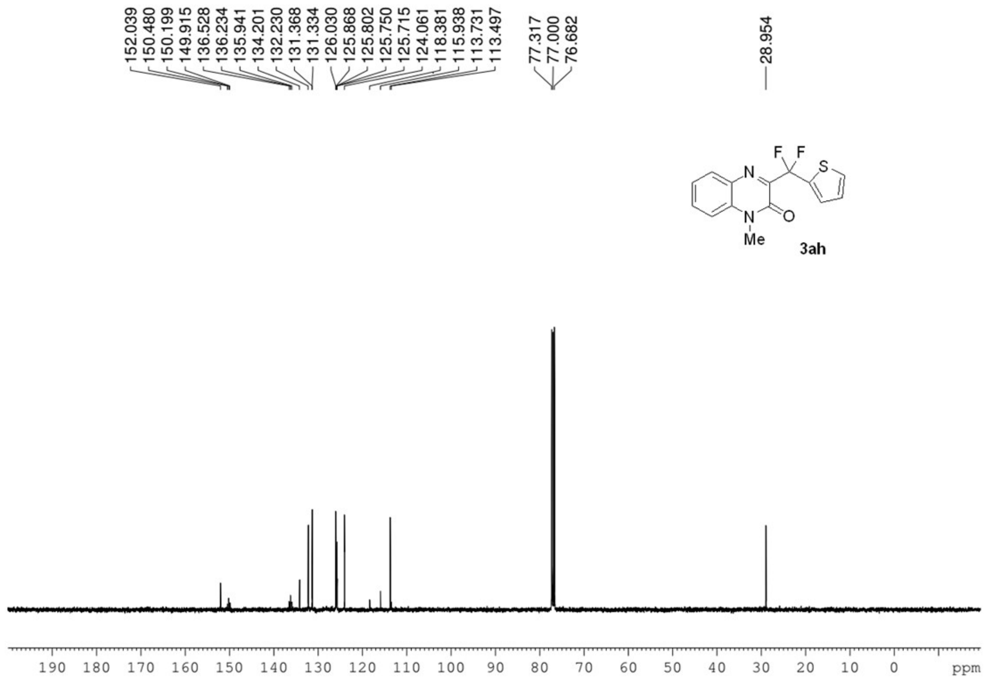
152.039
150.480
150.199
149.915
136.528
136.234
135.941
134.201
132.230
131.368
131.334
126.030
125.868
125.802
125.750
125.715
124.061
118.381
115.938
113.731
113.497
77.317
77.000
76.682
28.954
F F
N
S
O
N
Me
3ah
190 180 170 160 150 140 130 120 110 100 90 80 70 60 50 40 30 20 10 0 ppm

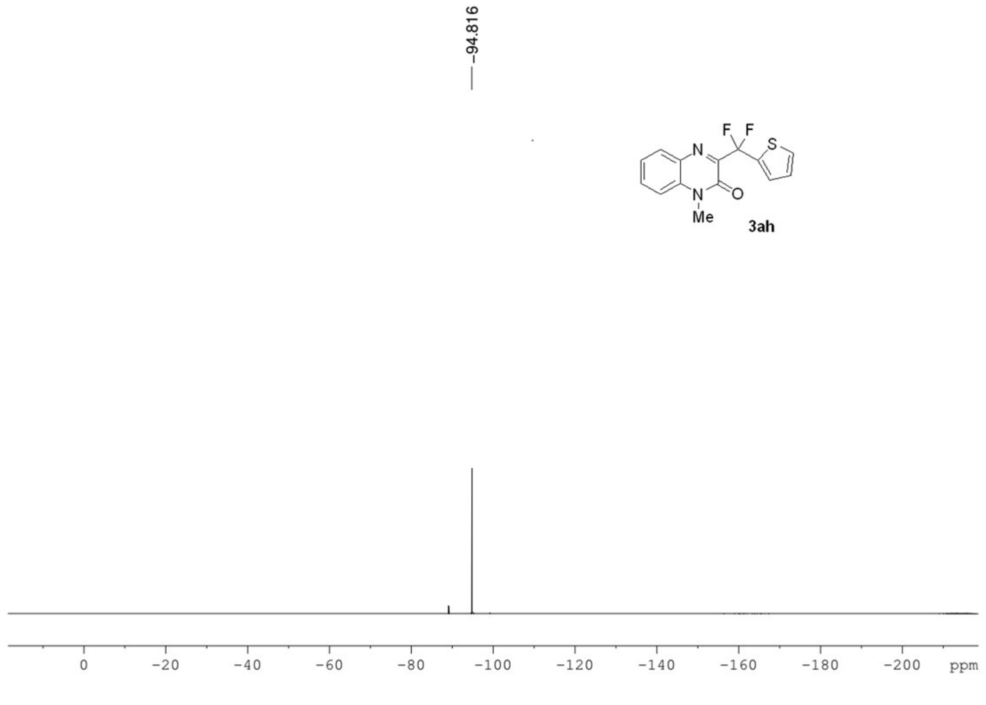
-94.816
F F
N
S
O
N
Me
3ah
0 -20 -40 -60 -80 -100 -120 -140 -160 -180 -200 ppm

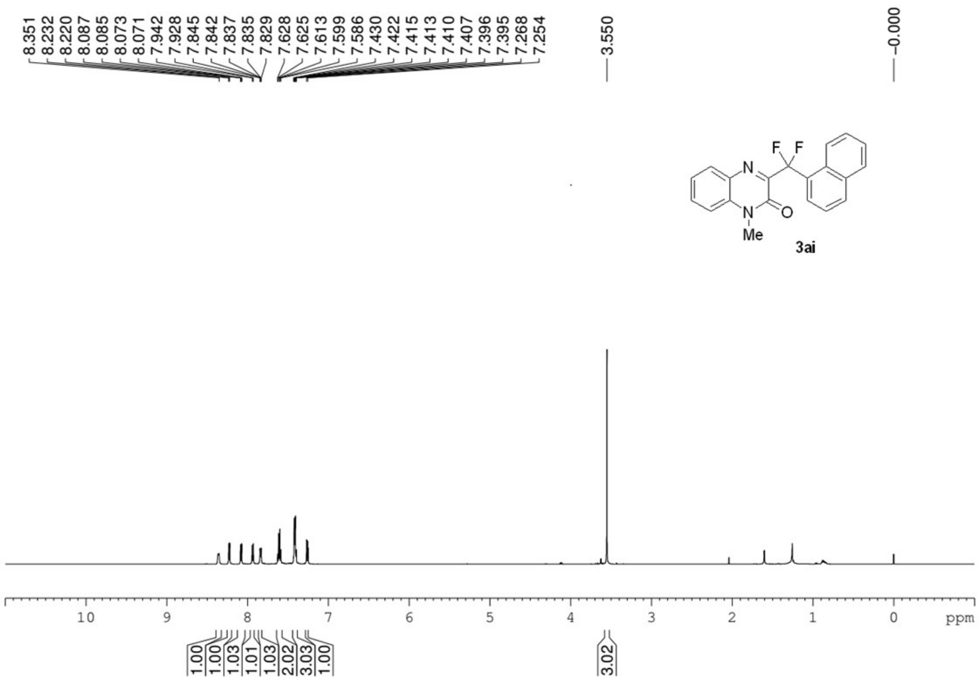
8.351
8.232
8.220
8.087
8.085
8.073
8.071
7.942
7.928
7.845
7.842
7.837
7.835
7.829
7.628
7.625
7.613
7.599
7.586
7.430
7.422
7.415
7.413
7.410
7.407
7.396
7.395
7.268
7.254
3.550
-0.000
F F
N
O
N
Me
3ai
1.00
1.00
1.03
1.01
1.03
2.02
3.03
1.00
3.02
10 9 8 7 6 5 4 3 2 1 0 ppm

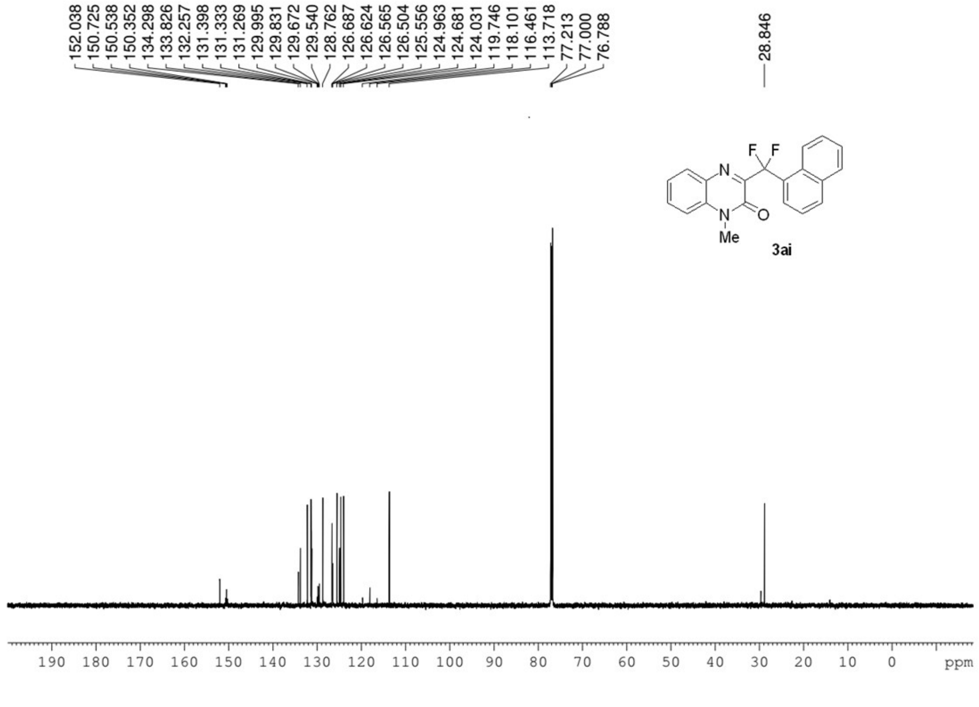
152.038
150.725
150.538
150.352
134.298
133.826
132.257
131.398
131.333
131.269
129.995
129.831
129.672
129.540
128.762
126.687
126.624
126.565
126.504
125.556
124.963
124.681
124.031
119.746
118.101
116.461
113.718
77.213
77.000
76.788
28.846
F F
N
O
N
Me
3ai
190 180 170 160 150 140 130 120 110 100 90 80 70 60 50 40 30 20 10 0 ppm

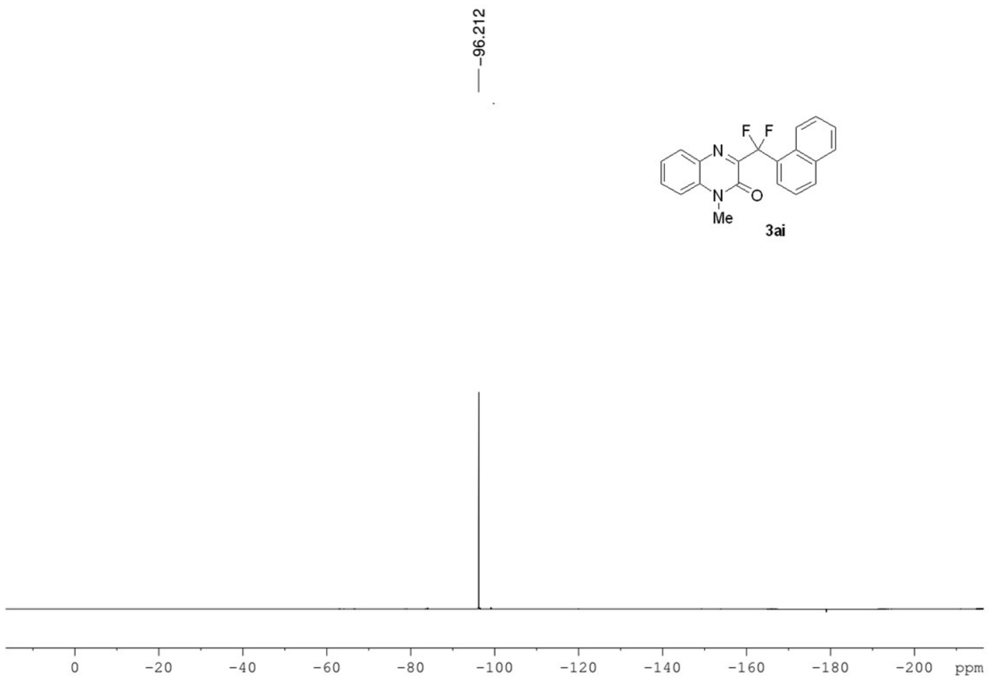

-96.212
F
F
N
N
O
Me
3ai
0
-20
-40
-60
-80
-100
-120
-140
-160
-180
-200
ppm

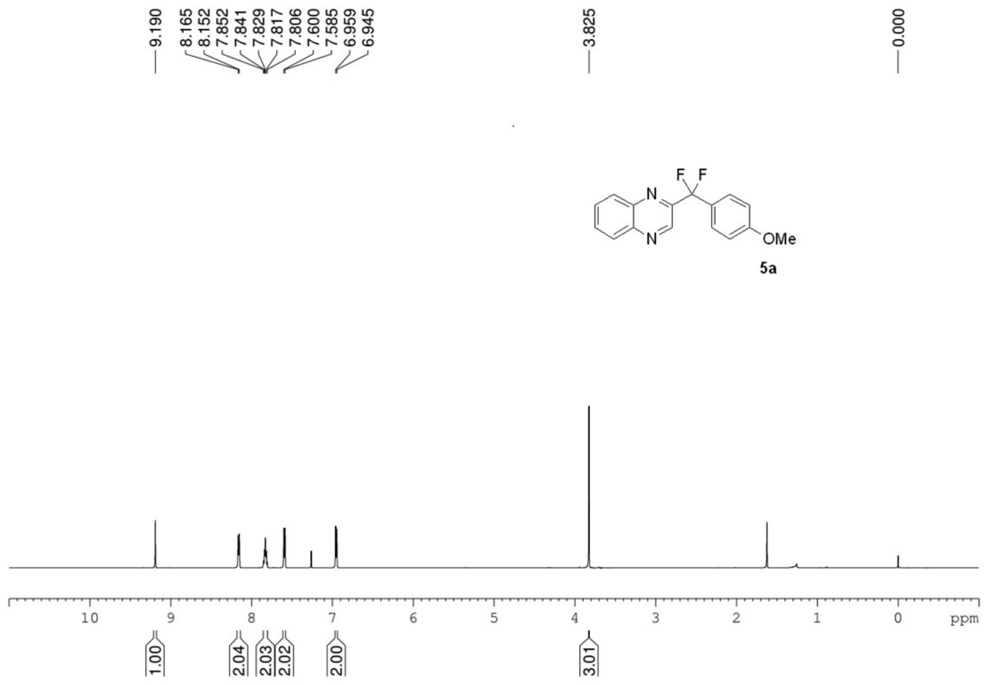

9.190
8.165
8.152
7.852
7.841
7.829
7.817
7.806
7.600
7.585
6.959
6.945
3.825
0.000
F
F
N
N
OMe
5a
10
9
8
7
6
5
4
3
2
1
0
ppm
1.00
2.04
2.03
2.02
2.00
3.01

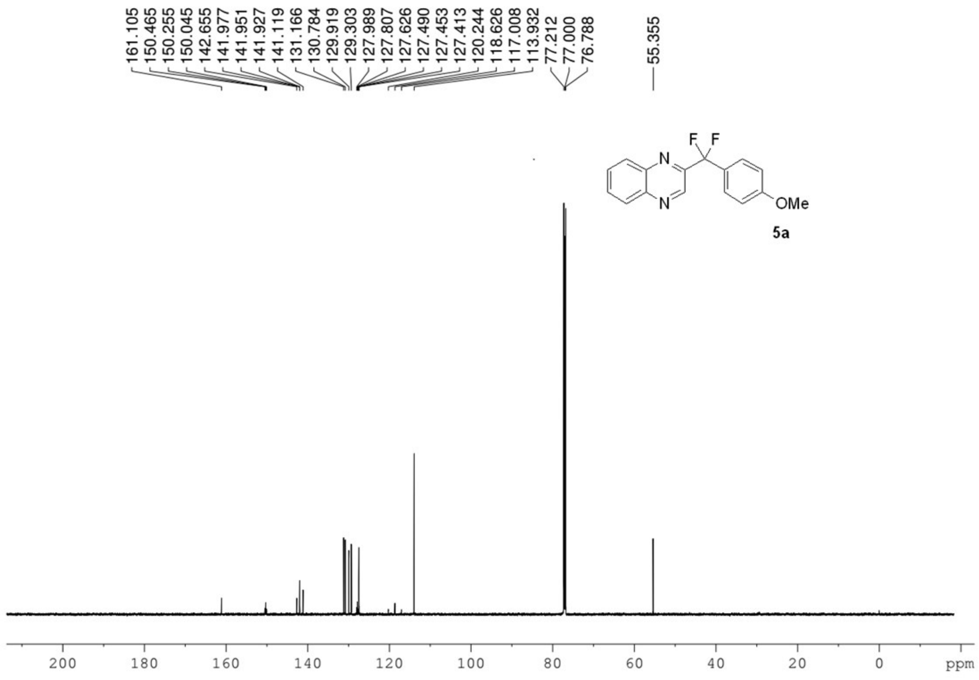
161.105
150.465
150.255
150.045
142.655
141.977
141.951
141.927
141.119
131.166
130.784
129.919
129.303
127.989
127.807
127.626
127.490
127.453
127.413
120.244
118.626
117.008
113.932
77.212
77.000
76.788
55.355
F F
N
N
OMe
5a
200 180 160 140 120 100 80 60 40 20 0 ppm

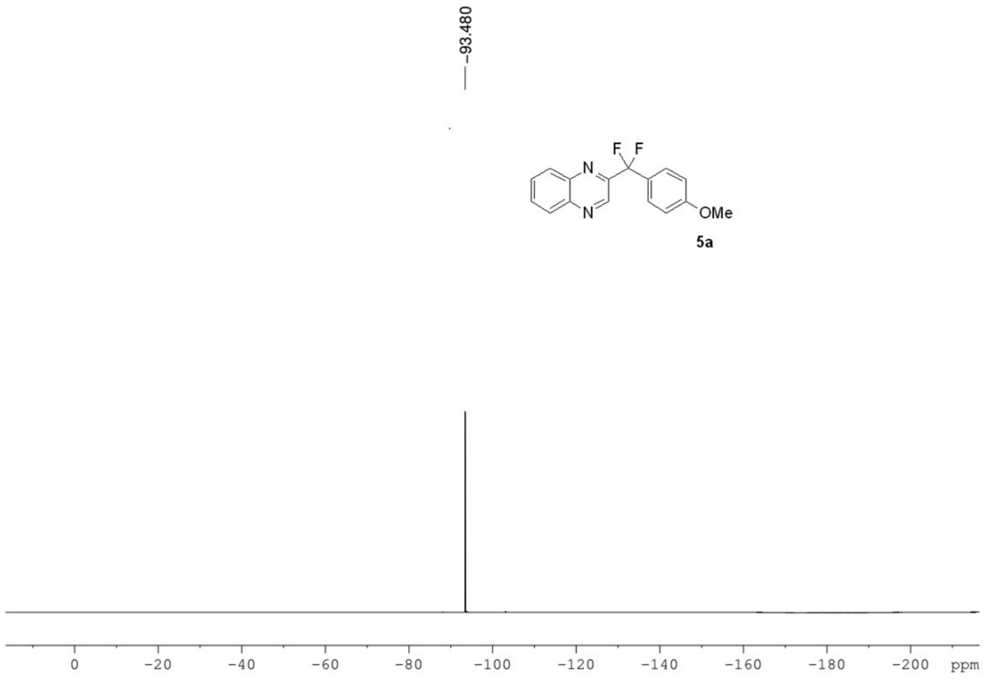
-93.480
F F
N
N
OMe
5a
0 -20 -40 -60 -80 -100 -120 -140 -160 -180 -200 ppm
